# Supplementary material for: Country of birth, educational level and other predictors of seeking care due to decreased fetal movements: an observational study in Sweden using data from a cluster-randomised controlled trial
Source: BMJ Open. 2021 Jun 25;11(6):e050621. doi: 10.1136/bmjopen-2021-050621 (PMC8237734; doi:10.1136/bmjopen-2021-050621)
Supplement: Supplementary data [file bmjopen-2021-050621supp001.pdf]

Supplementary Table 1. Percentage of women who sought care due to decreased fetal movements in Mindfetalness (*n*=19 639) versus routine care (*n*=20 226), divided into characteristics and a calculation of absolute differences between the groups.

**Characteristics**	**Mindfetalness** *n*=1287	**Percentage sought care**	**Routine care** *n*=772	**Percentage sought care**	**Absolute difference**
**Age**					
≤24 years	122/1483	8.2%	65/1322	4.9%	3.3%
25–34 years	859/12 715	6.8%	502/12 783	3.9%	2.9%
≥35 years	306/5441	5.6%	205/6111	3.4%	2.2%
**Country of birth**					
Sweden	1011/13 029	7.8%	584/13 456	4.3%	3.5%
Europe (except Sweden)	82/2026	4.0%	55/1891	2.9%	1.1%
Africa	28/1063	2.6%	23/1438	1.6%	1.0%
Asia	134/2989	4.5%	90/2898	3.1%	1.4%
North America	9/166	5.4%	7/154	4.5%	0.9%
South America	19/328	5.8%	12/325	3.7%	2.1%
Other	4/38	10.5%	1/64	1.6%	8.9%
**Education level¶**					
Shorter than 9 years	4/203	2.0%	7/359	1.9%	0.1%
Elementary school	48/916	5.2%	21/887	2.4%	2.8%
High school	339/5192	6.5%	207/5071	4.1%	2.4%
University	829/11 916	7.0%	461/12 088	3.8%	3.2%
**Parity §**					
Primipara	686/8544	8.0%	441/8927	4.9%	3.1%
Multipara	600/11 012	5.4%	330/11 242	2.9%	2.5%
**Assisted reproduction§**	79/1078	7.3%	50/1166	4.3%	3.0%
**Previous stillbirth**	9/116	7.8%	0/104	0%	7.8%
**Tobacco user at registration at the maternity clinic§**	56/737	7.6%	23/598	3.8%	3.8%
**Body Mass Index∫**					
<18.5 kg/m²	38/568	6.7%	16/507	3.2%	3.5%
18.5–24.9 kg/m²	771/11 828	6.5%	457/12 024	3.8%	2.7%
25.0–29.9 kg/m²	285/4336	6.6%	179/4739	3.8%	2.8%
30.0–34.9 kg/m²	107/1488	7.2%	60/1568	3.8%	3.4%
≥35.0 kg/m²	38/533	7.1%	25/530	4.7%	2.4%
**Maternal diseases***					
Psychiatric care	225/2468	9.1%	129/2630	4.9%	4.2%
Medication or psychologic treatment for mental illness	82/1160	7.1%	47/935	5.0%	2.1%

¶ Data are missing for 3,344 women (1,412 in Mindfetalness group and 1,932 in Routine care group)
§ Data are missing for 140 women (83 in Mindfetalness group and 57 in Routine care group)
∫ Data are missing for 1,744 women (886 in Mindfetalness group and 858 in Routine care group)
*Selected common diseases that can be associated with search behavior
