# Supplementary material for: Country of birth, educational level and other predictors of seeking care due to decreased fetal movements: an observational study in Sweden using data from a cluster-randomised controlled trial
Source: BMJ Open. 2021 Jun 25;11(6):e050621. doi: 10.1136/bmjopen-2021-050621 (PMC8237734; doi:10.1136/bmjopen-2021-050621)
Supplement: Supplementary data [file bmjopen-2021-050621supp002.pdf]

Supplementary Table 2. Obstetric outcome from gestation week 32+0 among women with singleton pregnancy who have contacted healthcare due to decreased fetal movements; 1287 registered at a maternity clinic randomized to Mindfetalness, and 772 registered at a maternity clinic randomized to routine care.

**Outcome**	**Mindfetalness** ***n* (%)**	**Routine care** ***n* (%)**	**Rate Ratio (95% CI)**	***p*-value**
Apgar Score <10 at 5 minutes†	147 (11.4)	88 (11.4)	1.00 (0.79–1.29)	0.97
Apgar Score <7 at 5 minutes†	15 (1.2)	10 (1.3)	0.90 (0.41–2.06)	0.80
Apgar Score < 4 at 5 minutes†	3 (0.2)	2 (0.3)	0.90 (0.15–6.84)	0.91
Birthweight <10th centile*‡	136 (10.6)	77 (10.0)	1.06 (0.82–1.39)	0.67
Birthweight <2SD**‡	41 (3.2)	22 (2.8)	1.12 (0.68–1.89)	0.67
Admitted to NICU	81 (6.3)	62 (8.0)	0.78 (0.57–1.08)	0.14
Death within 27 days after birth	0 (0.0)	0 (0.0)	NA	NA
Preterm delivery (<37+0)	34 (2.6)	15 (1.9)	1.36 (0.76–2.55)	0.31
Birth gestation >41+6	87 (6.8)	60 (7.8)	0.87 (0.64–1.20)	0.39
Spontaneous start of labor	854 (66.4)	486 (63.0)	1.05 (0.99–1.13)	0.12
Induction of labor	329 (25.6)	221 (28.6)	0.89 (0.77–1.03)	0.13
Cesarean section (total)	251 (19.5)	154 (19.9)	0.98 (0.82–1.17)	0.81
Pre–labor	106 (8.2)	65 (8.4)	0.98 (0.73–1.32)	0.88
In labor	145 (11.3)	89 (11.5)	0.98 (0.76–1.26)	0.86

Stillborn routine care *n*=1 (0.1%), Mindfetalness *n*=0 (0.0)
†Data are missing for 3 women (3 in Mindfetalness group and 0 in Routine care group)
‡Data are missing for 2 women (1 in Mindfetalness group and 1 in Routine care group)
*for the gestational age (International definition of Small for Gestational Age)
**from the national reference mean (Swedish definition of Small for Gestational Age)
NICU: Neonatal intensive care unit
