# Supplementary material for: Country of birth, educational level and other predictors of seeking care due to decreased fetal movements: an observational study in Sweden using data from a cluster-randomised controlled trial
Source: BMJ Open. 2021 Jun 25;11(6):e050621. doi: 10.1136/bmjopen-2021-050621 (PMC8237734; doi:10.1136/bmjopen-2021-050621)
Supplement: Supplementary data [file bmjopen-2021-050621supp003.pdf]

Mindfetalness – a method for focusing upon fetal movements

The first perception of fetal movements is sometimes described as a gentle tickling. As the pregnancy proceeds and the fetus develops the movements become more distinct. Just as new-born babies vary, there are differences between fetuses. Some fetuses are very active during their time in the uterus whilst others are calm, but all fetuses move up until birth. Each unborn baby has its own pattern of movements and at the end of the pregnancy the pattern can be recognised. The fetus moves between periods of wakefulness with much movement and calmer periods of rest. Movement frequency usually peaks around week 32 of pregnancy and remains for the most part at that level until delivery. The movements increase in strength as the fetus grows but at the end of the pregnancy can be experienced differently to the movements felt earlier.

That the fetus moves is a good sign and many pregnant women describe how they notice their unborn baby's movements every day. One systematic way of observing the movements is the method of *Mindfetalness* that can be used to get to know the movement pattern. An appropriate time to begin with *Mindfetalness* is in gestational week 28.

How to put Mindfetalness into practice

Mindfetalness is practiced ideally on a daily basis. **Choose a time of day that suits you best but also wait until you feel that your unborn baby is having a period of wakefulness.** Lie down or sit comfortably when you engage in Mindfetalness. If you lie down, then preferably on your left side. The movements are felt more distinctly when you lie down, and the blood flow is at its best in the uterus on the left side, which is good for the fetus. Concentrate on your unborn baby's movements for approximately 15 minutes. You will feel yourself if you need a longer or shorter time to perceive the movements. For some women it is enough just to get an idea of how the movements feel, others prefer to write notes about what they experience. At the end of this information there is space in which you can write down how you experience the movements. If you have a smartphone or computer you can write your impressions at www.mindfetalness.com

During Mindfetalness you focus upon:
The intensity of the movements
The way in which the baby moves
How much the baby moves

The questions to be answered are:
Can the movements be felt distinctly?
Are the movements of the same intensity as usual?
Does the fetus move as much as usual?

Fetal movements at the end of the pregnancy

The unborn baby's movements can be divided into two main groups: large movements and small movements. The large movements are felt distinctly; this can be when the fetus kicks or stretches out its body. The small movements that the fetus makes, but which are not felt, are gripping movements with fingers and toes as well as breathing movements. In approximately weeks 25 to 30 the movements begin to become organized and the unborn baby has periods of wakefulness interspersed with periods of rest lasting approximately 40 minutes up to an hour.

The movements change as the pregnancy proceeds and can feel different due to the fact that the space the fetus has at its disposal becomes smaller, although this does not affect the frequency of the movements. Women at full term pregnancy often describe how the movements feel powerful, pushing, stretching, large, from side to side, slow and light.

If you focus upon the fetal movements for a while every day when the fetus has a period of wakefulness, you can gain a good understanding of your unborn baby's movement pattern. It is important the observation occurs when the fetus is awake (the fetus is less active during a rest period). There can be wide variations from fetus to fetus regarding the frequency and intensity of the movements.

Women who have tried *Mindfetalness* describe how they felt calm, present and focussed while using the method. They also describe the period as a communication with their unborn baby and that they experienced a powerful bonding with their baby. Only you can decide whether the method suits you.

Summary

The movements become organised during pregnancy weeks 25 to 30 and the fetus has periods of wakefulness interspersed with periods of rest, approximately 40 minutes up to an hour. Most fetuses have, at the end of the pregnancy, a daily rhythm and are active in the evening.

In pregnancy week 32 a plateau phase is reported regarding the frequency of movements but there is nothing to indicate that the movements decrease at the end of pregnancy.

There can be a wide variation between fetuses in the frequency and intensity of their movements.

Get to know your unborn baby's movement pattern during pregnancy. Trust your intuition.

If you are concerned that the fetus is moving less or that the movements are weaker, you should contact health care.

What does the fetus do in the uterus?
As the fetus grows, the movements become more distinct and successively regular. Small movements are not felt, e.g. when the fetus sucks its thumb or flexes its toes. Kicks, and when the child stretches out, can usually be felt clearly and many also feel when the child hiccups (small rhythmic jerks) during the latter part of pregnancy.

During the final months of the pregnancy the movements are distinct and powerful, but may be experienced as being of a different character compared with when the fetus had a larger space at its disposal. Some women describe how the unborn baby stretches out, as if the fetus is trying to stretch as the space begins to be tight. Others describe the movements as large, that they involve the unborn baby's whole body and can be described as slow. The larger the fetus the more distinct the movement.

Is it true that the fetus moves less towards the end of pregnancy?
No, this is not true. Fetal movements increase up until pregnancy week 32, thereafter and up until delivery, the frequency of movements generally remains the same. It is important to remember that the fetus should continue to be active throughout the pregnancy.

Does the fetus move the whole time?
The fetus does not move the whole time. All unborn babies are calm and sleep for short periods. There can be wide variations from fetus to fetus regarding the frequency and intensity of the movements.

Can it be more difficult for some women to feel the movements?
It is probable that it is easier to feel the movements if the woman lies on her left side and concentrates on them. Some women describe how, in spite of doing so, they have great difficulty in feeling their unborn baby move. Extreme overweight, amongst other things, can make it more difficult. If one is much stressed it may be more difficult to feel the movements.

What should I do if I feel that the movements become fewer towards the end of the pregnancy?
If the movements decrease in intensity or frequency, and deviate from the fetus' normal way of moving, it can be a sign that the child is not doing so well in the uterus. Most pregnant women who experience fewer and weaker movements give birth to a healthy child, but there is an increased risk that the fetus is not fit. If you experience that the movements have become fewer and weaker, and you feel that there is a difference compared with earlier in the pregnancy it should *not* be interpreted as something normal until the child has been examined.

Diary of my unborn baby's activities

Pregnancy week	How my unborn baby moved
28 +	
Pregnancy week 29 +	How my unborn baby moved
Pregnancy week 30 +	How my unborn baby moved
Pregnancy week 31 +	How my unborn baby moved

Diary of my unborn baby's activities

Pregnancy week	How my unborn baby moved
32 +	
Pregnancy week 33 +	How my unborn baby moved
Pregnancy week 34 +	How my unborn baby moved
Pregnancy week 35 +	How my unborn baby moved

Radestad I, *et al. BMJ Open* 2021; 11:e050621. doi: 10.1136/bmjopen-2021-050621

Diary of my unborn baby's activities

Week of pregnancy	How my unborn baby moved
Week of pregnancy 36 +	How my unborn baby moved
Week of pregnancy 37 +	How my unborn baby moved
Week of pregnancy 38 +	How my unborn baby moved
Week of pregnancy 39 +	How my unborn baby moved

Diary of my unborn baby's activities

Week of pregnancy 40 +	How my unborn baby moved
Week of pregnancy 41 +	How my unborn baby moved
Week of pregnancy 42 +	How my unborn baby moved

Radestad I, *et al. BMJ Open* 2021; 11:e050621. doi: 10.1136/bmjopen-2021-050621
