# Supplementary material for: Country of birth, educational level and other predictors of seeking care due to decreased fetal movements: an observational study in Sweden using data from a cluster-randomised controlled trial
Source: BMJ Open. 2021 Jun 25;11(6):e050621. doi: 10.1136/bmjopen-2021-050621 (PMC8237734; doi:10.1136/bmjopen-2021-050621)
Supplement: Author's manuscript [file bmjopen-2021-050621.draft_revisions.pdf]

BMJ Open is committed to open peer review. As part of this commitment we make the peer review history of every article we publish publicly available.

When an article is published we post the peer reviewers' comments and the authors' responses online. We also post the versions of the paper that were used during peer review. These are the versions that the peer review comments apply to.

The versions of the paper that follow are the versions that were submitted during the peer review process. They are not the versions of record or the final published versions. They should not be cited or distributed as the published version of this manuscript.

BMJ Open is an open access journal and the full, final, typeset and author-corrected version of record of the manuscript is available on our site with no access controls, subscription charges or pay-per-view fees (http://bmjopen.bmj.com).

If you have any questions on BMJ Open's open peer review process please email info.bmjopen@bmj.com

BMJ Open

Country of birth, educational level and other predictors of seeking care due to decreased fetal movements – a population-based study

Journal:	*BMJ Open*
Manuscript ID	bmjopen-2021-050621
Article Type:	Original research
Date Submitted by the Author:	25-Feb-2021
Complete List of Authors:	Radestad, Ingela; Sophiahemmet University<br>Pettersson, Karin; Karolinska Institute, Department of Clinical Science, Intervention and Technology<br>Lindgren, Helena; Karolinska Institute, Women's and Children's Health<br>Skokic, Viktor; University of Gothenburg Sahlgrenska Academy, Oncology<br>Akselsson, Anna; Sophiahemmet University, Health Promoting Science
Keywords:	Fetal medicine < OBSTETRICS, Maternal medicine < OBSTETRICS, Prenatal diagnosis < OBSTETRICS

SCHOLARONE™
Manuscripts

I, the Submitting Author has the right to grant and does grant on behalf of all authors of the Work (as defined in the below author licence), an exclusive licence and/or a non-exclusive licence for contributions from authors who are: i) UK Crown employees; ii) where BMJ has agreed a CC-BY licence shall apply, and/or iii) in accordance with the terms applicable for US Federal Government officers or employees acting as part of their official duties; on a worldwide, perpetual, irrevocable, royalty-free basis to BMJ Publishing Group Ltd ("BMJ") its licensees and where the relevant Journal is co-owned by BMJ to the co-owners of the Journal, to publish the Work in this journal and any other BMJ products and to exploit all rights, as set out in our licence.

The Submitting Author accepts and understands that any supply made under these terms is made by BMJ to the Submitting Author unless you are acting as an employee on behalf of your employer or a postgraduate student of an affiliated institution which is paying any applicable article publishing charge ("APC") for Open Access articles. Where the Submitting Author wishes to make the Work available on an Open Access basis (and intends to pay the relevant APC), the terms of reuse of such Open Access shall be governed by a Creative Commons licence – details of these licences and which Creative Commons licence will apply to this Work are set out in our licence referred to above.

Other than as permitted in any relevant BMJ Author's Self Archiving Policies, I confirm this Work has not been accepted for publication elsewhere, is not being considered for publication elsewhere and does not duplicate material already published. I confirm all authors consent to publication of this Work and authorise the granting of this licence.

For peer review only

Country of birth, educational level and other predictors of seeking care due to decreased fetal movements – a population-based study

Ingela Rådestad, RNM, Professor, Sophiahemmet University, Stockholm, Sweden.
E-mail: ingela.radestad@shh.se

Karin Pettersson, MD, Associate Professor, Department of Clinical Science, Intervention and Technology, Karolinska Institutet, Stockholm, Sweden. E-mail: karin.ma.pettersson@sll.se

Helena Lindgren, Associate Professor, Department of Women's and children's health, Karolinska Institutet, Stockholm, Sweden. E-mail: Helena.lindgren@ki.se

Viktor Skokic, Statistician, Institute of Clinical Sciences, Sahlgrenska Academy, University of Gothenburg, Gothenburg, Sweden. E-mail: viktor.skokic@gu.se

Anna Akselsson*, RNM, PhD, Department of Health Promoting Science, Sophiahemmet University, Karolinska Institutet, Department of Women's and children's health, Stockholm, Sweden. E-mail: anna.akselsson@shh.se

For peer review only

*Corresponding author
Anna Akselsson, RNM, PhD
Sophiahemmet University
PB 5605, S-114 86, Stockholm, Sweden.
E-mail: anna.akselsson@shh.se
Phone: +46 8 406 20 79

For peer review only - http://bmjopen.bmj.com/site/about/guidelines.xhtml

Abstract

Design Cluster-randomized controlled trial.

Setting 67 maternity clinics and six obstetrical clinics in Sweden.

Participants All pregnant women with a singleton pregnancy who contacted the obstetrical clinic due to decreased fetal movements from 32 weeks' gestation (n=2059 out of 39 865 included in the trial).

Interventions Routine care or Mindfetalness- a self-assessment method for the women to use daily when the baby is awake to become familiar with the unborn baby's fetal movement pattern; focus on movements character, strength and frequency (without counting each movement).

Outcome measures Predictors for contacting healthcare due to decreased fetal movements and whether the Mindfetalness method affects women's care-seeking behavior.

Results Overall, 5.2% of women contacted healthcare due to decreased fetal movements, out of 1287 women (62.5%) registered at a maternity clinic randomized to Mindfetalness and 772 women (37.5%) randomized to routine care. Contact due to decreased fetal movements decreased with increased age ($p<0.001$). Women born outside Sweden sought care due to decreased fetal movements to a lower extent compared to Swedish-born women ($p<0.001$). A lower percentage of women with low educational levels contacted healthcare due to decreased fetal movements compared to women with university-level education ($p<0.001$). Introducing Mindfetalness in maternity care increased the number of women seeking care due to decreased fetal movements overall.

Conclusion Women with country of origin outside Sweden and low educational level sought care for decreased fetal movements to a lesser extent compared to women born in Sweden and those with university degrees. Future research could explore whether pregnancy outcomes can be improved by motivating women in these groups to contact healthcare if they feel a decreased strength or frequency of fetal movements.

Trial registration www.clinicaltrials.gov (NCT02865759).

For peer review only - http://bmjopen.bmj.com/site/about/guidelines.xhtml

Strengths and limitations of this study

- This is the first study exploring the association between country of birth and contact due to decreased fetal movements.

- The new information about predictors for contacting healthcare due to decreased fetal movements can be valuable for preventing stillbirth.

- Zero lost to follow up, large sample size and population-based register is a methodological strength.

- Misclassified subjects might have affected result of the predictors and it is probably an underestimation of the number of women seeking care.

- Possible confounders, not being considered in the study, could have had an impact of the result.

For peer review only

Introduction

A woman's interpretation of signals from the fetus and her ability to take action will determine whether she contacts healthcare when she has concerns for her unborn baby's health. Her response to altered fetal movement patterns can be decisive for the pregnancy outcomes. Among women who have sought care due to decreased fetal movements, reports indicate that one quarter will later experience adverse baby outcomes, such as low Apgar score, small for gestational age, or stillbirth [1-3]. Further, up to fifty percent of women who experience stillbirth report a gradual decrease in fetal movements several days before the baby died [4,5]. Thus, the majority waited more than 24 hours before contacting healthcare without the perception of any movements [6,7]. Contact with healthcare due to decreased fetal movements is common and most of the women return home after normal findings are confirmed. The prevalence of women contacting healthcare with concerns of fetal movements varies internationally; from six to 34 percent [8-11].

Women with normal pregnancies can distinguish between many different types of fetal movements and most feel strong movements [12]. Maternal perception of fetal movements and becoming familiar with the movement pattern is important in reducing stillbirth, as reported in research from New Zealand [13]. The character and diurnal rhythm of movements seem to be essential factors in preventing stillbirth. Maternally perceived increased strength of fetal movements is associated with reduced stillbirth rate (aOR 0.21, 95% CI 0.12–0.36) and quiet or

For peer review only - http://bmjopen.bmj.com/site/about/guidelines.xhtml

light movements in the evening were associated with higher risk (aOR 3.82, 95% CI 1.57–9.31, $p < 0.0001$).

Raising maternal awareness of fetal movements by using the Mindfetalness method may be a way forward for women to become familiar with the unborn baby's movement pattern [14]. In a cluster randomized controlled trial, 67 maternity clinics were allocated to either Mindfetalness or routine care [15,16]. Midwives in the Mindfetalness group were instructed to distribute oral and written information about fetal movements and about Mindfetalness. With the method, the women are instructed to lie down for 15 minutes a day from 28 weeks' gestation, when the baby is awake and to focus on the character, strength and frequency of the unborn baby's movements, without counting each movement. Information regarding the method was available in nine different languages and a website was open for anyone to access. The trial showed several benefits of introducing Mindfetalness into maternity care [15]. In the Mindfetalness group, more women started their labor spontaneously, followed by a reduction in labor inductions and cesarean sections. Further, less babies were born small for gestational age and needed neonatal care.

In this study, we investigate predictors of seeking care for decreased fetal movements and whether care-seeking behavior was influenced by a method (Mindfetalness) aimed at increasing pregnant women's knowledge of their unborn baby's fetal movement patterns. [14]. We further investigate pregnancy outcomes for women seeking care for decreased fetal movements but where, at the time of the examination, no interventions such as induction of labor or caesarean section were made.

Methods

In a cluster-randomized controlled trial, 67 maternity clinics in Stockholm, Sweden, were randomized to either intervention with Mindfetalness or routine care, as reported previously [15,16]. The randomization resulted in 33 maternity clinics in the Mindfetalness group and 34 in routine care group. After the randomization, one clinic randomized to Mindfetalness declined participation. Starting in October 2016, midwives, working at maternity clinics randomized to the intervention, distributed a leaflet to pregnant women, including information about fetal movements and the self-assessment method, Mindfetalness. The last leaflet was distributed on 31 January 2018, and the women were followed-up until birth in population-based registers.

For peer review only - http://bmjopen.bmj.com/site/about/guidelines.xhtml

Midwives in the routine care group followed standard care, which includes providing oral information about fetal movements at 24 weeks' gestation.

The Mindfetalness leaflet (appendix) included information about fetal movements and when to contact healthcare; "If you are concerned that the fetus is moving less or that the movements are weaker, you should contact healthcare." Further, the leaflet included an invitation to practice Mindfetalness daily from 28 weeks' gestation. The women are instructed to lie down on the left side 15 minutes a day, when the baby is awake, and focus on the intensity, character and frequency of the movements, without counting each movement. The information was available in nine different languages and the website www.mindfetalness.com was open for anyone to access.

Pregnant women with a singleton pregnancy who contacted healthcare due to decreased fetal movements from 32 weeks' gestation constitute the population of this study. We applied intention-to-treat design; extended information about the randomization process can be found in previous publications [15,16]. The Mindfetalness group included 19 639 women and the routine care group, 20 226 women. The women who contacted healthcare due to decreased fetal movements from 32 weeks' gestation were generated from the ICD-code AM041 (examination due to decreased fetal movements) [17]. The code is a diagnosis for pregnant women who have contacted healthcare due to decreased fetal movements, where no interventions, such as labor induction or cesarean section, were made, at the time the woman sought care.

This study was approved by the Regional Ethics committee in Stockholm, Sweden (Dnr 2015/2105–31/1), 13 January 2016.

Statistical analysis

We identified the background factors of the women who contacted healthcare due to decreased fetal movements by calculating percentages. The included variables were age, country of origin, education level, parity, assisted reproduction, previous stillbirth, tobacco use at registration in maternity clinic, body mass index, and maternal diseases. To calculate predictors for contacting healthcare, we investigated which factors were statistically significantly associated with contact due to decreased fetal movements. As a metric for association, we calculated relative risks and 95% confidence intervals using log-binomial regression. In the predictor calculation, we adjusted for the possible confounding factor, parity. For outcome measures, we calculated percentage and

For peer review only - http://bmjopen.bmj.com/site/about/guidelines.xhtml

compared the Mindfetalness group with the routine care group using log-binomial regression. We considered a *p*-value of 0.05 to be statistically significant. We used the statistical program R (version 3.2.4).

Results

Among all women in this study, 5.2% (*n*=2059) contacted healthcare due to decreased fetal movements, measured from gestational week 32 until birth. As shown in Figure 1, 62.5% (*n*=1287) were registered at a maternity clinic randomized to Mindfetalness, and 37.5% (*n*=772) to routine care. The background data for the women are displayed in Table 1.

Table 1. Characteristics among women with a singleton pregnancy from 32 weeks' gestation contacting healthcare versus not contacting healthcare due to decreased fetal movements.

**Characteristics**	**Contacted healthcare due to decreased fetal movements (*n*=2059)** *n* (%)	**Did not contact healthcare due to decreased fetal movements (*n*=37 806)** *n* (%)	**Relative risk (CI)**	***p*-value**
**Age***				
≤24 years	187 (9.1)	2628 (7.0)	1.31 (1.13–1.51)	<0.001
25–34 years	1361 (66.1)	24 137 (63.8)	1.04 (1.00–1.07)	0.04
≥35 years	511 (24.8)	11 041 (29.2)	0.85 (0.79–0.92)	<0.001
**Country of origin**				
Sweden	1595 (77.5)	24 890 (65.8)	1.18 (1.15–1.21)	<0.001
Europe (except Sweden)	137 (6.7)	3780 (10.0)	0.67 (0.56–0.79)	<0.001
Africa	51 (2.5)	2450 (6.5)	0.38 (0.29–0.50)	<0.001
Asia	224 (10.9)	5663 (15.0)	0.73 (0.64–0.82)	<0.001
North America	16 (0.8)	304 (0.8)	0.97 (0.59–1.60)	1.00
South America	31 (1.5)	622 (1.6)	0.92 (0.64–1.31)	0.72
Others	5 (0.2)	97 (0.3)	0.95 (0.39–2.33)	1.00
**Education level¶**				
Shorter than 9 years	11 (0.5)	551 (1.5)	0.36 (0.20–0.66)	<0.001
Elementary school	69 (3.4)	1734 (4.6)	0.72 (0.57–0.91)	0.006
High school	546 (26.5)	9717 (25.7)	1.02 (0.95–1.10)	0.64
University	1290 (62.7)	22 714 (60.1)	1.03 (1.00–1.06)	0.09
**Parity §**				
Primipara	1127 (54.7)	16 344 (43.2)	1.26 (1.21–1.32)	<0.001
Multipara	930 (45.2)	21 324 (56.4)	0.80 (0.76–0.84)	<0.001
**Assisted reproduction§**	129 (6.3)	2033 (5.4)	1.17 (0.98–1.38)	0.09
**Previous stillbirth**	9 (0.4)	211 (0.6)	0.78 (0.40–1.52)	0.64
**Tobacco user at registration in the maternity clinic (including snuff) §**	79 (3.8)	1256 (3.3)	1.16 (0.92–1.44)	0.21
**Body Mass Index***∫**				
<18.5 kg/m²	54 (2.6)	1021 (2.7)	0.97 (0.74–1.27)	0.89
18.5–24.9 kg/m²	1228 (59.6)	22 624 (59.8)	0.99 (0.96–1.03)	0.70
25.0–29.9 kg/m²	464 (22.5)	8611 (22.8)	0.99 (0.91–1.07)	0.74

For peer review only - http://bmjopen.bmj.com/site/about/guidelines.xhtml

30.0–34.9 kg/m²	167 (8.1)	2889 (7.6)	1.06 (0.91–1.23)	0.47
≥35.0 kg/m²	63 (3.1)	1000 (2.6)	1.15 (0.90–1.48)	0.26
**Maternal diseases**				
Diabetes mellitus	1 (0.0)	63 (0.2)	0.29 (0.04–2.10)	0.26
Coronary heart disease	25 (1.2)	600 (1.6)	0.77 (0.51–1.14)	0.20
Thrombosis	13 (0.6)	283 (0.7)	0.84 (0.49–1.47)	0.69
Systemic lupus erythematosus (SLE)	1 (0.0)	58 (0.2)	0.32 (0.04–2.28)	0.37
Psychiatric care	354 (17.2)	4744 (12.5)	1.37 (1.24–1.51)	<0.001
Endocrine disease	156 (7.6)	2633 (7.0)	1.09 (0.93–1.27)	0.29
Epilepsy	10 (0.5)	167 (0.4)	1.10 (0.58–2.08)	0.73
Chronic hypertension	9 (0.4)	184 (0.5)	0.90 (0.46–1.75)	0.87
Other disease	204 (9.9)	3583 (9.5)	1.05 (0.91–1.20)	0.51
Medication or psychologic treatment for mental illness	129 (6.3)	2058 (5.4)	1.15 (0.97–1.37)	0.11

* Mean age of women who contacted care=31.2, who did not contact care=32.4 ($p<0.001$)
¶ Data missing for women who contacted care n=143 (6.9%), who did not contact care n=3090 (8.2%)
§ Data missing for women who contacted care n=2 (0.1%), who did not contact care n=138 (0.4%)
∫ Data missing for women who contacted care n=83 (4.0%), who did not contact care n=1661 (4.4%)

The number of women seeking care for decreased fetal movements decreased by increased age ($p<0.001$) (Table 2). As compared to women born in Sweden, the relative risk for seeking care due to decreased fetal movements was lower for women born outside Sweden ($p<0.001$).

Table 2. Predictors for contacting healthcare due to decreased fetal movements

Predictor	*p-value*	*n (%)*	*RR (CI)*	*p-value*	*AdjRR (CI)**
Age	<0.001			0.02	
≤24 years		187 (6.6)	1.24 (1.07–1.44)		1.15 (0.98–1.33)
25–34 years		1361 (5.3)	1.0 (Reference)		1.0 (Reference)
≥35 years		511 (4.4)	0.83 (0.75–0.91)		0.90 (0.82–1.00)
Country of origin	<0.001			<0.001	
Sweden		1595 (6.0)	1.0 (Reference)		1.0 (Reference)
Europe (except Sweden)		137 (3.5)	0.58 (0.49–0.69)		0.58 (0.49 – 0.69)
Africa		51 (2.0)	0.34 (0.25–0.44)		0.36 (0.27–0.47)
Asia		224 (3.8)	0.63 (0.55–0.72)		0.66 (0.57–0.75)
North America		16 (5.0)	0.83 (0.49–1.29)		0.83 (0.49–1.28)
South America		31 (4.7)	0.79 (0.54–1.09)		0.82 (0.56–1.13)
Others		5 (4.9)	0.81 (0.30–1.70)		0.84 (0.31–1.75)
Marital status	0.20			0.06	
Cohabiting with becoming father		1914 (5.2)	1.0 (Reference)		1.0 (Reference)
Single		29 (4)	0.76 (0.52–1.07)		0.73 (0.49–1.02)
Other family situation		114 (4.8)	0.92 (0.76–1.10)		0.87 (0.72–1.04)
Education level	<0.001			<0.001	
Shorter than 9 years		11 (2.0)	0.36 (0.19–0.62)		0.41 (0.21–0.69)
Elementary school		69 (3.8)	0.71 (0.56–0.90)		0.75 (0.58–0.94)
High school		546 (5.3)	0.99 (0.90–1.09)		1.00 (0.91–1.10)
University		1290 (5.4)	1.0 (Reference)		1.0 (Reference)
Parity	<0.001			NA	
Primipara		1127 (6.5)	1.54 (1.42–1.68)		NA
Multipara		930 (4.2)	1.0 (Reference)		NA

For peer review only

Gestation week >41+6	<0.001			0.01	
No			1.0 (Reference)		1.0 (Reference)
Yes			1.34 (1.13–1.56)		1.25 (1.06–1.46)
Assisted reproduction	0.10			0.55	
No		1928 (5.1)	1.0 (Reference)		1.0 (Reference)
Yes		129 (6.0)	1.16 (0.97–1.38)		1.05 (0.88–1.25)
Previous stillbirth	0.46			0.95	
No		2050 (5.2)	1.0 (Reference)		1.0 (Reference)
Yes		9 (4.1)	0.79 (0.38–1.41)		0.98 (0.47–1.74)
Tobacco user at registration at the maternity clinic§	0.22			0.17	
No			1.0 (Reference)		1.0 (Reference)
Yes		79 (5.9)	1.15 (0.92–1.42)		1.17 (0.93–1.44)
Body Mass Index	0.77			0.37	
<18.5 kg/m²		54 (5.0)	0.98 (0.74–1.26)		0.95 (0.72–1.23)
18.5–24.9 kg/m²		1228 (5.1)	1.0 (Reference)		1.0 (Reference)
25.0–29.9 kg/m²		464 (5.1)	0.99 (0.89–1.10)		1.03 (0.92–1.14)
30.0–34.9 kg/m²		167 (5.5)	1.06 (0.90–1.24)		1.12 (0.95–1.31)
≥35.0 kg/m²		63 (5.9)	1.15 (0.89–1.46)		1.22 (0.94–1.54)
Psychiatric care	<0.001			<0.001	
No		1705 (4.9)	1.0 (Reference)		1.0 (Reference)
Yes		354 (6.9)	1.42 (1.27–1.58)		1.37 (1.22–1.52)
Medication or psychologic treatment for mental illness	0.12			0.13	
No		1930 (5.1)	1.0 (Reference)		1.0 (Reference)
Yes		129 (5.9)	1.15 (0.96–1.36)		1.15 (0.96–1.36)

*Adjusted for parity

Women with low educational levels sought care due to decreased fetal movements to a lower extent compared to women with university-level education ($p<0.001$). Further, primiparas, women with prolonged pregnancies (>41+6 gestation weeks), and women with experience of psychiatric care more often sought care due to decreased fetal movements (Table 2). The predictors for contacting healthcare due to decreased fetal movements remain after adjustment for parity. Mindfetalness increased the prevalence of women contacting healthcare due to decreased fetal movements overall (6.6% versus 3.8%, RR 1.72, CI 1.57–1.87) (not in table). When stratifying women into groups according to background factors, the percentage of women contacting healthcare increased in all groups.

Figures 2a and 2b show the pregnancy outcomes for women contacting healthcare due to decreased fetal movements, stratified for women registered at maternity clinics randomized to Mindfetalness and routine care, respectively. Compared to routine care, women in the Mindfetalness group had a higher percentage of women with spontaneous start of labor (66.4%

For peer review only

versus 63.0%) and a lower rate of labor induction (25.6% versus 28.6%). The proportion of women who had onset of labor from 42 weeks' gestation was 6.8% in the Mindfetalness group, and 7.8% in routine care. Further, a lower proportion of babies in the Mindfetalness group were in need of transfer to NICU (6.3% versus 8.0%). One stillbirth was observed, in the routine care group (not in table). There were no statistically significant differences in the investigated pregnancy outcomes in Figures 2a and 2b, between the Mindfetalness and routine care groups. For further information in pregnancy outcomes for Mindfetalness and routine care, see Supplementary Table 1.

Discussion

During the studied period, in the Stockholm region, women born outside Sweden and women with low levels of education sought care for decreased fetal movements to a lower extent when compared to women born in Sweden and women with university-level education. Further, women of higher age contacted healthcare due to decreased fetal movements to a lower extent than women of a younger age. Country of birth outside Sweden, low educational level and advanced maternal age have previously been related to adverse birth outcomes. Introducing Mindfetalness in maternity care increased the number of women contacting healthcare due to decreased fetal movements.

We found that women born outside Sweden (mostly from Africa and Asia) sought care due to decreased fetal movements to a lower extent. No studies, to our knowledge, have investigated the association between contact with healthcare due to maternal concern of fetal movements and country of origin. However, previous research has reported a higher risk of stillbirth among women not being born, but giving birth, in a Western country [18-23]. Suggested mechanisms for this difference include sub-standard maternity care, lack of communication between the pregnant woman and healthcare professionals, and variations in pre-pregnancy health [19,24]. It is possible that the higher risk for stillbirth can be linked to lower awareness and knowledge about fetal movements and when to contact healthcare [6,7].

Giving information to pregnant women about fetal movements may reduce pre-hospital delays [25-27] and has been suggested to reduce the stillbirth rate in an intervention study performed in Norway[27]. The study documented a 50 percent reduction in the stillbirth rate among women who

For peer review only - http://bmjopen.bmj.com/site/about/guidelines.xhtml

contacted healthcare due to decreased fetal movements as compared to women who did not. The intervention included providing health promotion information to the pregnant women, an invitation to count fetal movements, and information that they should never wait until the next day to contact healthcare if they had concerns about fetal movements. Additionally, providing new guidelines to the healthcare professionals was included in the intervention. A reduction in patient delay and increased knowledge about fetal movements was shown in a recent study from the Netherlands after the introduction of a leaflet about fetal movements [28]. However, in the AFFIRM-trial[29], women in the intervention arm (including information to pregnant women and new guidelines to the healthcare professionals) had lower rate of stillbirths than women in the control group, but the difference was not statistically significant. Labor inductions and cesarean section increased, and the number of babies in need of neonatal care more than 48 hours.

The midwife is the most important source of information [30,31] and The Swedish National Board of Health and Welfare recently introduced new guidelines for maternity care, including instructions to midwives to inform pregnant women about fetal movements [32]. The guidelines have good compliance among midwives (79-87% answered yes to the different questions whether they followed the guidelines), but some report barriers due to lack of time, high workload and problems in communicating with women born outside of Sweden [33]. Suboptimal care is more common among non-western women and action to improve perinatal mortality for this group is needed [19].

In this study, women with low education levels sought care due to decreased fetal movements to a lower extent, as compared to women with high education levels. A higher risk for stillbirth has been found among women with low educational levels and low-socioeconomic status [6,34-36]. There are reasons to believe that raising awareness about fetal movements among women with low education or socioeconomic status would increase the number of women seeking care due to decreased fetal movements, which in turn would possibly decrease perinatal mortality.

Women of advanced maternal age contacted healthcare due to decreased fetal movements to a lower extent than younger women in our study, and a higher risk of stillbirth is reported among women aged from 35 years compared to younger women [37]. The mean age in an Israeli study was lower among women who had contacted healthcare due to decreased fetal movements compared to the reference group [38]. In a study from Norway, the percentage of women over 35 years of age

For peer review only - http://bmjopen.bmj.com/site/about/guidelines.xhtml

was lower among women who contacted healthcare due to decreased fetal movements compared to the reference group (16% versus 20%) [3]. However, in a recent systematic review, the authors interpreted current evidence as not supporting the notion that a woman's mean age influences whether she seeks care for decreased fetal movements [39].

Introducing Mindfetalness in maternity care increased the number of women contacting healthcare due to decreased fetal movements overall. Mindfetalness possibly increases the woman's ability to become familiar with the unborn baby's fetal movement patterns. Reducing pre-hospital delay when the perception that fetal movements are reduced is suggested to be one way for improving perinatal mortality [7,25,26].

Methodological considerations

The prevalence of women seeking care due to decreased or altered fetal movements in our study is low compared to the figures from other research. In a study performed in Sweden in 2014, 9.3 percent of the women sought care due to decreased fetal movements when measured from gestational week 28 until birth [9]. Further, international studies show a prevalence of 6–34 percent [8,10,11,40]. We believe that the figures in our study are underestimated, mostly due to the fact that we only included women who sought care from gestational week 32. Additionally, the ICD-code AM041 is not the best tool to use for including all women who contact healthcare due to decreased fetal movements, but it is the only code available. Misclassification in the study may be important to consider as some women who have sought care due to decreased fetal movements probably didn't get the ICD-code. In the analyze, these women were classified as they had not sought care due to decreased fetal movements, which might have affected the result. But, when comparing the Mindfetalness group with routine care group, only non-differential misclassification is likable, which do not affect the results differently in the two groups.

The only potential confounder we adjusted for was parity. We do not have information about other possibly confounders, for example, if some of the women had extra appointments in specialist maternity clinics, which may have affected their care-seeking behavior. Extra pre-booked meetings with midwives or obstetricians and investigations with cardiotocography or ultrasound due to other complication can also decrease the need for unscheduled visits at the hospitals.

For peer review only - http://bmjopen.bmj.com/site/about/guidelines.xhtml

When evaluating whether Mindfetalness has an effect on women contacting healthcare due to decreased fetal movements, the random allocation, the large sample size, and zero lost to follow-up are strengths of the study. Further, the missing data are negligible, thanks to the unique population-based register in Sweden. While the intention-to-treat design does preserve the advantages of random allocation, it also risks misclassification and the dilution of the results, i.e., it is an underestimation of the true effects. From the pilot study, we know that approximately 75 percent of the Swedish-speaking women use the method [41], and, from the main trial, about 22 percent did not receive a leaflet [15]. Additionally, the information was available in nine languages and, in Stockholm, Sweden, there is a large variety of women who originate from different countries and who speak languages that were not included in the intervention and this may have diluted the effect. This further contributes to the possible dilution of effects.

Implications

The National Board of Health and Welfare claim that pregnant women should receive information about risk factors for adverse pregnancy outcomes, including stillbirth [33]. Women born in low-income countries and women with advanced maternal age are factors to consider when trying to improve perinatal mortality in high-income countries [37,42]. Providing targeted information, available in a variety of languages, to these women about fetal movements and when to contact healthcare might help to improve perinatal outcomes. Also, providing information about how to practice Mindfetalness can be one way forward to reduce adverse pregnancy outcomes. Today, we have a great number of opportunities to reach pregnant women thanks to the internet and to target women in at-risk groups. Additionally, this information can be provided in different forms, for example, videos, apps and interactive webpages.

Conclusion

We do not know for certain what the ideal frequency is for women seeking care due to decreased fetal movements, but we can conclude that it is lower in women who have migrated to Sweden from, for example, Africa and Asia, and in women with low educational levels. Women from low-income countries have documented worse pregnancy outcomes. An implication of our results may be to examine whether pregnancy outcomes can be improved by providing customized information to women from low-income countries with the goal that they seek care if they feel a decreased strength or frequency of fetal movements. More data are needed to draw conclusions

For peer review only - http://bmjopen.bmj.com/site/about/guidelines.xhtml

For peer review only

about the effects of Mindfetalness in perinatal mortality among women contacting healthcare due to decreased fetal movements.

Acknowledgements

The authors would like to thank the midwives and the pregnant women in the maternity clinics in Stockholm. Thanks to the coordination midwives and medical doctors at Mödrahälsovårdsenheten and to The Swedish Pregnancy Register for cooperation. Special thanks to The Swedish Research Council for funding this study.

For peer review only

For peer review only - http://bmjopen.bmj.com/site/about/guidelines.xhtml

References

1. Dutton PJ, Warrander LK, Roberts SA, et al. Predictors of poor perinatal outcome following maternal perception of reduced fetal movements--a prospective cohort study. *PLoS One* 2012; **7**(7): e39784.
2. Higgins LE, Myers JE, Sibley CP, Johnstone ED, Heazell AEP. Antenatal placental assessment in the prediction of adverse pregnancy outcome after reduced fetal movement. *PLoS One* 2018; **13**(11): e0206533.
3. Holm Tveit JV, Saastad E, Stray-Pedersen B, Bordahl PE, Froen JF. Maternal characteristics and pregnancy outcomes in women presenting with decreased fetal movements in late pregnancy. *Acta obstetricia et gynecologica Scandinavica* 2009; **88**(12): 1345-51.
4. Linde A, Pettersson K, Radestad I. Women's Experiences of Fetal Movements before the Confirmation of Fetal DeathContractions Misinterpreted as Fetal Movement. *Birth-Issues in Perinatal Care* 2015; **42**(2): 189-94.
5. Warland J, O'Brien LM, Heazell AE, Mitchell EA. An international internet survey of the experiences of 1,714 mothers with a late stillbirth: the STARS cohort study. *BMC pregnancy and childbirth* 2015; **15**: 172.
6. Froen JF, Arnestad M, Frey K, Vege A, Saugstad OD, Stray-Pedersen B. Risk factors for sudden intrauterine unexplained death: epidemiologic characteristics of singleton cases in Oslo, Norway, 1986-1995. *American journal of obstetrics and gynecology* 2001; **184**(4): 694-702.
7. Koshida S, Ono T, Tsuji S, Murakami T, Arima H, Takahashi K. Excessively delayed maternal reaction after their perception of decreased fetal movements in stillbirths: Population-based study in Japan. *Women and birth : journal of the Australian College of Midwives* 2017.
8. Kapaya H, Almeida J, Karouni F, Anumba D. Management of reduced fetal movement: A comparative analysis of two audits at a tertiary care clinical service. *Eur J Obstet Gynecol Reprod Biol* 2020; **248**: 128-32.
9. Akselsson A, Lindgren H, Georgsson S, Pettersson K, Radestad I. Increased labor induction and women presenting with decreased or altered fetal movements - a population-based survey. *PLoS One* 2019; **14**(5): e0216216.
10. Froen JF. A kick from within--fetal movement counting and the cancelled progress in antenatal care. *J Perinat Med* 2004; **32**(1): 13-24.
11. Sergent F, Lefevre A, Verspyck E, Marpeau L. [Decreased fetal movements in the third trimester: what to do?]. *Gynecologie, obstetrique & fertilite* 2005; **33**(11): 861-9.
12. Radestad I, Lindgren H. Women's perceptions of fetal movements in full-term pregnancy. *Sexual & reproductive healthcare : official journal of the Swedish Association of Midwives* 2012; **3**(3): 113-6.
13. Bradford BF, Cronin RS, McCowan LME, McKinlay CJD, Mitchell EA, Thompson JMD. Association between maternally perceived quality and pattern of fetal movements and late stillbirth. *Scientific reports* 2019; **9**(1): 9815.
14. Radestad I. Strengthening mindfetalness. *Sexual & reproductive healthcare : official journal of the Swedish Association of Midwives* 2012; **3**(2): 59-60.
15. Akselsson A, Lindgren H, Georgsson S, et al. Mindfetalness to increase women's awareness of fetal movements and pregnancy outcomes: a cluster-randomised controlled trial including 39 865 women. *BJOG : an international journal of obstetrics and gynaecology* 2020.
16. Radestad I, Akselsson A, Georgsson S, Lindgren H, Pettersson K, Steineck G. Rationale, study protocol and the cluster randomization process in a controlled trial including 40,000 women investigating the effects of mindfetalness. *Sexual & reproductive healthcare : official journal of the Swedish Association of Midwives* 2016; **10**: 56-61.
17. ICD internetmedicin. 2018. http://icd.internetmedicin.se/ (accessed December 2020).

For peer review only - http://bmjopen.bmj.com/site/about/guidelines.xhtml

18. Gagnon AJ, Zimbeck M, Zeitlin J, et al. Migration to western industrialised countries and perinatal health: a systematic review. *Soc Sci Med* 2009; **69**(6): 934-46.
19. Saastad E, Vangen S, Froen JF. Suboptimal care in stillbirths - a retrospective audit study. *Acta obstetricia et gynecologica Scandinavica* 2007; **86**(4): 444-50.
20. Khalil A, Rezende J, Akolekar R, Syngelaki A, Nicolaides KH. Maternal racial origin and adverse pregnancy outcome: a cohort study. *Ultrasound Obstet Gynecol* 2013; **41**(3): 278-85.
21. Ravelli AC, Tromp M, Eskes M, et al. Ethnic differences in stillbirth and early neonatal mortality in The Netherlands. *J Epidemiol Community Health* 2011; **65**(8): 696-701.
22. Drysdale H, Ranasinha S, Kendall A, Knight M, Wallace EM. Ethnicity and the risk of late-pregnancy stillbirth. *Med J Aust* 2012; **197**(5): 278-81.
23. Vik ES, Aasheim V, Schytt E, Small R, Moster D, Nilsen RM. Stillbirth in relation to maternal country of birth and other migration related factors: a population-based study in Norway. *BMC pregnancy and childbirth* 2019; **19**(1): 5.
24. Essen B, Bodker B, Sjoberg NO, et al. Are some perinatal deaths in immigrant groups linked to suboptimal perinatal care services? *BJOG : an international journal of obstetrics and gynaecology* 2002; **109**(6): 677-82.
25. Koshida S, Ono T, Tsuji S, Murakami T, Takahashi K. Recommendations for preventing stillbirth: a regional population-based study in Japan during 2007-2011. *Tohoku J Exp Med* 2015; **235**(2): 145-9.
26. RCOG. Reduced Fetal Movements. 2011 https://www.rcog.org.uk/en/guidelines-research-services/guidelines/gtg57/57).
27. Tveit JV, Saastad E, Stray-Pedersen B, et al. Reduction of late stillbirth with the introduction of fetal movement information and guidelines - a clinical quality improvement. *BMC pregnancy and childbirth* 2009; **9**: 32.
28. Wackers K, Wassen M, Zeegers B, Bude L, Nieuwenhuijze MJ. Effect of the use of a national information brochure about fetal movements on patient delay. *Women and birth : journal of the Australian College of Midwives* 2019; **32**(2): 131-6.
29. Norman JE, Heazell AEP, Rodriguez A, et al. Awareness of fetal movements and care package to reduce fetal mortality (AFFIRM): a stepped wedge, cluster-randomised trial. *Lancet* 2018.
30. Grimes HA, Forster DA, Newton MS. Sources of information used by women during pregnancy to meet their information needs. *Midwifery* 2014; **30**(1): e26-33.
31. McArdle A, Flenady V, Toohill J, Gamble J, Creedy D. How pregnant women learn about foetal movements: sources and preferences for information. *Women and birth : journal of the Australian College of Midwives* 2015; **28**(1): 54-9.
32. Socialstyrelsen. Minskade fosterrörelser – rekommendationer om information, råd och en inledande bedömning – Kunskapsstöd med nationella rekommendationer. 2016. http://www.socialstyrelsen.se/publikationer2016/2016-10-9 (accessed december 2020).
33. Socialstyrelsen. Dödfödda barn - En inventering och förslag på åtgärder. 2018. https://www.socialstyrelsen.se/publikationer2018/2018-12-36/Sidor/default.aspx (accessed december 2020).
34. Stephansson O, Dickman PW, Johansson AL, Cnattingius S. The influence of socioeconomic status on stillbirth risk in Sweden. *Int J Epidemiol* 2001; **30**(6): 1296-301.
35. Zeitlin J, Mortensen L, Prunet C, et al. Socioeconomic inequalities in stillbirth rates in Europe: measuring the gap using routine data from the Euro-Peristat Project. *BMC pregnancy and childbirth* 2016; **16**: 15.
36. Socialstyrelsen. Socioekonomiska faktorers påverkan på kvinnors och barns hälsa efter förlossning 2016. file:///H:/Artiklar/Socialstyrelsen/Socioekonomiska%20faktorer%20risk%20negativt%20utfall.pdf (accessed 01-28 2019).

For peer review only - http://bmjopen.bmj.com/site/about/guidelines.xhtml

37. Flenady V, Middleton P, Smith GC, et al. Stillbirths: the way forward in high-income countries. *Lancet* 2011; **377**(9778): 1703-17.
38. Levy M, Kovo M, Barda G, et al. Reduced fetal movements at term, low-risk pregnancies: is it associated with adverse pregnancy outcomes? Ten years of experience from a single tertiary center. *Archives of gynecology and obstetrics* 2020; **301**(4): 987-93.
39. Carroll L, Gallagher L, Smith V. Risk factors for reduced fetal movements in pregnancy: A systematic review and meta-analysis. *Eur J Obstet Gynecol Reprod Biol* 2019; **243**: 72-82.
40. Moore TR, Piacquadio K. A prospective evaluation of fetal movement screening to reduce the incidence of antepartum fetal death. *American journal of obstetrics and gynecology* 1989; **160**(5 Pt 1): 1075-80.
41. Akselsson A, Georgsson S, Lindgren H, Pettersson K, Radestad I. Women's attitudes, experiences and compliance concerning the use of Mindfetalness- a method for systematic observation of fetal movements in late pregnancy. *BMC pregnancy and childbirth* 2017; **17**(1): 359.
42. Flenady V, Koopmans L, Middleton P, et al. Major risk factors for stillbirth in high-income countries: a systematic review and meta-analysis. *Lancet* 2011; **377**(9774): 1331-40.

For peer review only

For peer review only - http://bmjopen.bmj.com/site/about/guidelines.xhtml

Author contributions

IR, KP, HL and AA were involved in the design and planning of the cluster-randomized controlled trial and IR obtained funding. AA had overall responsibility for the study. VS and AA analysed the data with input from IR, KP and HL. AA wrote the original manuscript and all authors made critical input. All authors were responsible for drafting and validating the following versions of the manuscript. All authors approved the final version of the manuscript.

Funding: This study was funded by The Swedish Research Council. Grant number not applicable.

Competing interests: The authors declare that they have no competing interests and there are not any financial competing interests.

Patient consent: Not required.

Ethics approval: This study was approved by the Regional Ethics committee in Stockholm, Sweden (Dnr 2015/2105–31/1), 13 January 2016.

Data sharing statement No additional data is available. Anonymised data are available after ethical approval by the Regional Ethics committee in Stockholm, Sweden and after request to The Swedish Pregnancy Register.

Patient and Public Involvement: Patients or the public were not involved in the design, or conduct, or reporting, or dissemination plans of our research.

For peer review only

Figure 1. Flow chart.

Figure 2a. Birth outcome among women who contacted healthcare due to decreased fetal movements from 32 weeks' gestation, where the examination did not indicate that the pregnancy needed to be terminated, at the time the women sought care.

Figure 2b. Start of labor among women who contacted healthcare due to decreased fetal movements from 32 weeks' gestation, where the examination did not indicate that the pregnancy needed to be terminated, at the time the women sought care.

For peer review only - http://bmjopen.bmj.com/site/about/guidelines.xhtml

Figure 1. Flow chart.

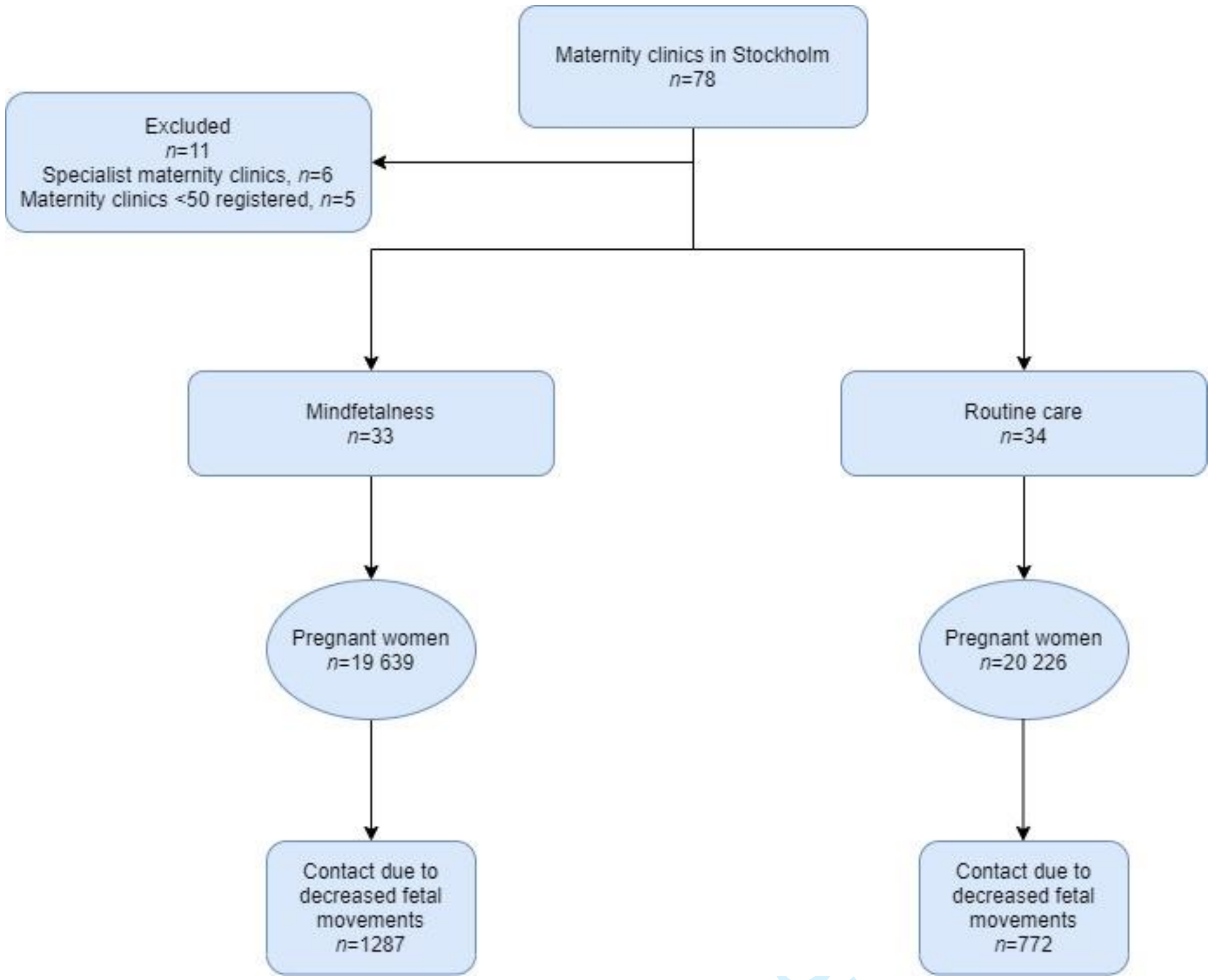

Figure 2a. Birth outcome among women who contacted healthcare due to decreased fetal movements from 32 weeks' gestation, where the examination did not indicate that the pregnancy needed to be terminated, at the time the women sought care.

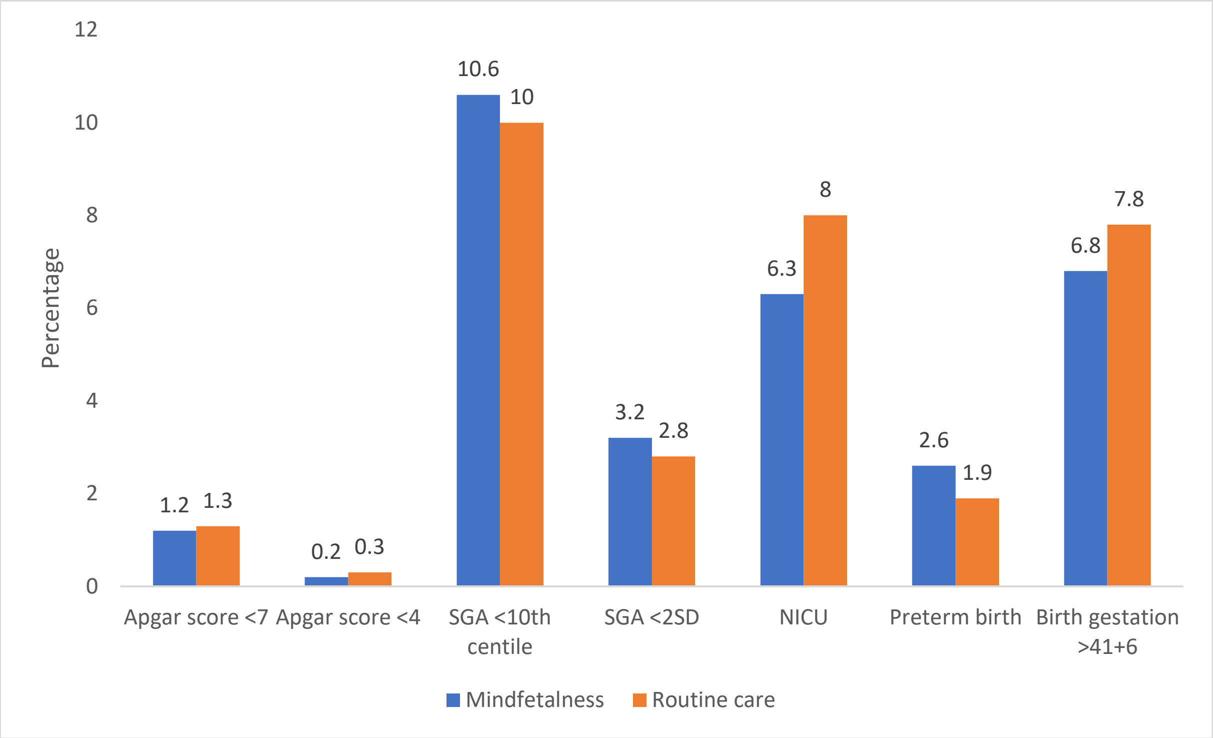

For peer review only - http://bmjopen.bmj.com/site/about/guidelines.xhtml

Figure 2b. Start of labor among women who contacted healthcare due to decreased fetal movements from 32 weeks' gestation, where the examination did not indicate that the pregnancy needed to be terminated, at the time the women sought care.

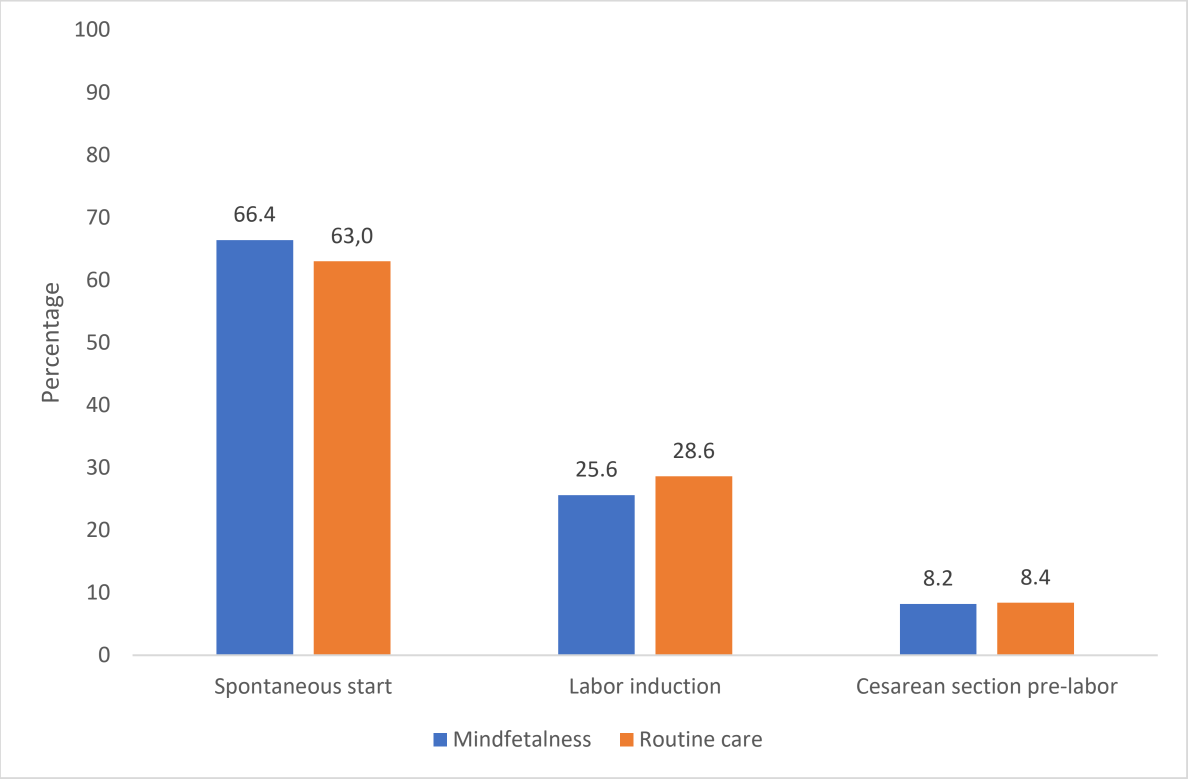

For peer review only - http://bmjopen.bmj.com/site/about/guidelines.xhtml

Supplementary Table 1. Obstetric outcome from gestation week 32+0 among women with singleton pregnancy who have contacted healthcare due to decreased fetal movements; 1287 registered at a maternity clinic randomized to Mindfetalness, and 772 registered at a maternity clinic randomized to routine care.

**Outcome**	**Mindfetalness** ***n*** **(%)**	**Routine care** ***n*** **(%)**	**Rate Ratio (95% CI)**	***p*-value**
Apgar Score <10 at 5 minutes†	147 (11.4)	88 (11.4)	1.00 (0.79–1.29)	0.97
Apgar Score <7 at 5 minutes†	15 (1.2)	10 (1.3)	0.90 (0.41–2.06)	0.80
Apgar Score < 4 at 5 minutes†	3 (0.2)	2 (0.3)	0.90 (0.15–6.84)	0.91
Birthweight <10th centile*‡	136 (10.6)	77 (10.0)	1.06 (0.82–1.39)	0.67
Birthweight <2SD**‡	41 (3.2)	22 (2.8)	1.12 (0.68–1.89)	0.67
Admitted to NICU	81 (6.3)	62 (8.0)	0.78 (0.57–1.08)	0.14
Death within 27 days after birth	0 (0.0)	0 (0.0)	NA	NA
Preterm delivery (<37+0)	34 (2.6)	15 (1.9)	1.36 (0.76–2.55)	0.31
Birth gestation >41+6	87 (6.8)	60 (7.8)	0.87 (0.64–1.20)	0.39
Spontaneous start of labor	854 (66.4)	486 (63.0)	1.05 (0.99–1.13)	0.12
Induction of labor	329 (25.6)	221 (28.6)	0.89 (0.77–1.03)	0.13
Cesarean section (total)	251 (19.5)	154 (19.9)	0.98 (0.82–1.17)	0.81
Pre–labor	106 (8.2)	65 (8.4)	0.98 (0.73–1.32)	0.88
In labor	145 (11.3)	89 (11.5)	0.98 (0.76–1.26)	0.86

Stillborn routine care *n*=1 (0.1%), Mindfetalness *n*=0 (0.0)
†Data are missing for 3 women (3 in Mindfetalness group and 0 in Routine care group)
‡Data are missing for 2 women (1 in Mindfetalness group and 1 in Routine care group)
*for the gestational age (International definition of Small for Gestational Age)
**from the national reference mean (Swedish definition of Small for Gestational Age)
NICU: Neonatal intensive care unit

For peer review only

Mindfetalness – a method for focusing upon fetal movements

The first perception of fetal movements is sometimes described as a gentle tickling. As the pregnancy proceeds and the fetus develops the movements become more distinct. Just as new-born babies vary, there are differences between fetuses. Some fetuses are very active during their time in the uterus whilst others are calm, but all fetuses move up until birth. Each unborn baby has its own pattern of movements and at the end of the pregnancy the pattern can be recognised. The fetus moves between periods of wakefulness with much movement and calmer periods of rest. Movement frequency usually peaks around week 32 of pregnancy and remains for the most part at that level until delivery. The movements increase in strength as the fetus grows but at the end of the pregnancy can be experienced differently to the movements felt earlier.

That the fetus moves is a good sign and many pregnant women describe how they notice their unborn baby's movements every day. One systematic way of observing the movements is the method of *Mindfetalness* that can be used to get to know the movement pattern. An appropriate time to begin with *Mindfetalness* is in gestational week 28.

How to put Mindfetalness into practice

Mindfetalness is practiced ideally on a daily basis. **Choose a time of day that suits you best but also wait until you feel that your unborn baby is having a period of wakefulness.** Lie down or sit comfortably when you engage in Mindfetalness. If you lie down, then preferably on your left side. The movements are felt more distinctly when you lie down, and the blood flow is at its best in the uterus on the left side, which is good for the fetus. Concentrate on your unborn baby's movements for approximately 15 minutes. You will feel yourself if you need a longer or shorter time to perceive the movements. For some women it is enough just to get an idea of how the movements feel, others prefer to write notes about what they experience. At the end of this information there is space in which you can write down how you experience the movements. If you have a smartphone or computer you can write your impressions at www.mindfetalness.com

During Mindfetalness you focus upon:

The intensity of the movements
The way in which the baby moves
How much the baby moves

The questions to be answered are:

Can the movements be felt distinctly?
Are the movements of the same intensity as usual?
Does the fetus move as much as usual?

For peer review only

For peer review only - http://bmjopen.bmj.com/site/about/guidelines.xhtml

Fetal movements at the end of the pregnancy

The unborn baby's movements can be divided into two main groups: large movements and small movements. The large movements are felt distinctly; this can be when the fetus kicks or stretches out its body. The small movements that the fetus makes, but which are not felt, are gripping movements with fingers and toes as well as breathing movements. In approximately weeks 25 to 30 the movements begin to become organized and the unborn baby has periods of wakefulness interspersed with periods of rest lasting approximately 40 minutes up to an hour.

The movements change as the pregnancy proceeds and can feel different due to the fact that the space the fetus has at its disposal becomes smaller, although this does not affect the frequency of the movements. Women at full term pregnancy often describe how the movements feel powerful, pushing, stretching, large, from side to side, slow and light.

If you focus upon the fetal movements for a while every day when the fetus has a period of wakefulness, you can gain a good understanding of your unborn baby's movement pattern. It is important the observation occurs when the fetus is awake (the fetus is less active during a rest period). There can be wide variations from fetus to fetus regarding the frequency and intensity of the movements.

Women who have tried *Mindfetalness* describe how they felt calm, present and focussed while using the method. They also describe the period as a communication with their unborn baby and that they experienced a powerful bonding with their baby. Only you can decide whether the method suits you.

Summary

The movements become organised during pregnancy weeks 25 to 30 and the fetus has periods of wakefulness interspersed with periods of rest, approximately 40 minutes up to an hour. Most fetuses have, at the end of the pregnancy, a daily rhythm and are active in the evening.

In pregnancy week 32 a plateau phase is reported regarding the frequency of movements but there is nothing to indicate that the movements decrease at the end of pregnancy.

There can be a wide variation between fetuses in the frequency and intensity of their movements.

Get to know your unborn baby's movement pattern during pregnancy. Trust your intuition.

If you are concerned that the fetus is moving less or that the movements are weaker, you should contact health care.

For peer review only - http://bmjopen.bmj.com/site/about/guidelines.xhtml

What does the fetus do in the uterus?
As the fetus grows, the movements become more distinct and successively regular. Small movements are not felt, e.g. when the fetus sucks its thumb or flexes its toes. Kicks, and when the child stretches out, can usually be felt clearly and many also feel when the child hiccups (small rhythmic jerks) during the latter part of pregnancy.

During the final months of the pregnancy the movements are distinct and powerful, but may be experienced as being of a different character compared with when the fetus had a larger space at its disposal. Some women describe how the unborn baby stretches out, as if the fetus is trying to stretch as the space begins to be tight. Others describe the movements as large, that they involve the unborn baby's whole body and can be described as slow. The larger the fetus the more distinct the movement.

Is it true that the fetus moves less towards the end of pregnancy?
No, this is not true. Fetal movements increase up until pregnancy week 32, thereafter and up until delivery, the frequency of movements generally remains the same. It is important to remember that the fetus should continue to be active throughout the pregnancy.

Does the fetus move the whole time?
The fetus does not move the whole time. All unborn babies are calm and sleep for short periods. There can be wide variations from fetus to fetus regarding the frequency and intensity of the movements.

Can it be more difficult for some women to feel the movements?
It is probable that it is easier to feel the movements if the woman lies on her left side and concentrates on them. Some women describe how, in spite of doing so, they have great difficulty in feeling their unborn baby move. Extreme overweight, amongst other things, can make it more difficult. If one is much stressed it may be more difficult to feel the movements.

What should I do if I feel that the movements become fewer towards the end of the pregnancy?
If the movements decrease in intensity or frequency, and deviate from the fetus' normal way of moving, it can be a sign that the child is not doing so well in the uterus. Most pregnant women who experience fewer and weaker movements give birth to a healthy child, but there is an increased risk that the fetus is not fit. If you experience that the movements have become fewer and weaker, and you feel that there is a difference compared with earlier in the pregnancy it should *not* be interpreted as something normal until the child has been examined.

For peer review only - http://bmjopen.bmj.com/site/about/guidelines.xhtml

Diary of my unborn baby's activities

Pregnancy week	How my unborn baby moved
28 +	
Pregnancy week 29 +	How my unborn baby moved
Pregnancy week 30 +	How my unborn baby moved
Pregnancy week 31 +	How my unborn baby moved

For peer review only

For peer review only - http://bmjopen.bmj.com/site/about/guidelines.xhtml

Diary of my unborn baby's activities

Pregnancy week	How my unborn baby moved
32 +	
**Pregnancy week** 33 +	**How my unborn baby moved**
**Pregnancy week** 34 +	**How my unborn baby moved**
**Pregnancy week** 35 +	**How my unborn baby moved**

For peer review only

For peer review only - http://bmjopen.bmj.com/site/about/guidelines.xhtml

Diary of my unborn baby's activities

Week of pregnancy	How my unborn baby moved
Week of pregnancy 36 +	How my unborn baby moved
Week of pregnancy 37 +	How my unborn baby moved
Week of pregnancy 38 +	How my unborn baby moved
Week of pregnancy 39 +	How my unborn baby moved

For peer review only

For peer review only - http://bmjopen.bmj.com/site/about/guidelines.xhtml

Diary of my unborn baby's activities

Week of pregnancy 40 +	How my unborn baby moved
**Week of pregnancy 41 +**	**How my unborn baby moved**
**Week of pregnancy 42 +**	**How my unborn baby moved**

For peer review only

For peer review only - http://bmjopen.bmj.com/site/about/guidelines.xhtml

Table 1: CONSORT 2010 checklist of information to include when reporting a cluster randomised trial

Section/Topic	Item No	Standard Checklist item	Extension for cluster designs	Page No *
**Title and abstract**				
	1a	Identification as a randomised trial in the title	Identification as a cluster randomised trial in the title	No, this is a subanalysis within an RCT
	1b	Structured summary of trial design, methods, results, and conclusions (for specific guidance see CONSORT for abstracts)[1,2]	See table 2	Yes
**Introduction**				
**Background and objectives**	2a	Scientific background and explanation of rationale	Rationale for using a cluster design	Yes, page 4
	2b	Specific objectives or hypotheses	Whether objectives pertain to the the cluster level, the individual participant level or both	Yes, page 4, individual participant level
**Methods**				
**Trial design**	3a	Description of trial design (such as parallel, factorial) including allocation ratio	Definition of cluster and description of how the design features apply to the clusters	Yes, page 4-5
	3b	Important changes to methods after trial commencement (such as eligibility criteria), with reasons		No changes done
**Participants**	4a	Eligibility criteria for participants	Eligibility criteria for clusters	Yes, page 4-5
	4b	Settings and locations where the data were collected		Yes, page 4-5
**Interventions**	5	The interventions for each group with sufficient details to allow replication, including how and when they were actually administered	Whether interventions pertain to the cluster level, the individual participant level or both	Yes, page 4-5

For peer review

**Outcomes**	6a	Completely defined pre-specified primary and secondary outcome measures, including how and when they were assessed	Whether outcome measures pertain to the cluster level, the individual participant level or both	Yes, individual level and specified in advance
	6b	Any changes to trial outcomes after the trial commenced, with reasons		No
**Sample size**	7a	How sample size was determined	Method of calculation, number of clusters(s) (and whether equal or unequal cluster sizes are assumed), cluster size, a coefficient of intracluster correlation (ICC or *k*), and an indication of its uncertainty	Yes, short version 4-5, longer version previous articles
	7b	When applicable, explanation of any interim analyses and stopping guidelines		Not applicable
**Randomisation:**				
**Sequence generation**	8a	Method used to generate the random allocation sequence		Yes, short version 4-5, longer version previous articles
	8b	Type of randomisation; details of any restriction (such as blocking and block size)	Details of stratification or matching if used	Yes, page 4-5
**Allocation concealment mechanism**	9	Mechanism used to implement the random allocation sequence (such as sequentially numbered containers), describing any steps taken to conceal the sequence until interventions were assigned	Specification that allocation was based on clusters rather than individuals and whether allocation concealment (if any) was at the cluster level, the individual participant level or both	Yes, individual level, page 4-5
**Implementation**	10	Who generated the random allocation sequence, who enrolled participants, and who assigned participants to interventions	Replace by 10a, 10b and 10c	

For peer review only

	10a		Who generated the random allocation sequence, who enrolled clusters, and who assigned clusters to interventions	Yes, page 4-5 and previous articles
	10b		Mechanism by which individual participants were included in clusters for the purposes of the trial (such as complete enumeration, random sampling)	Yes, page 4-5
	10c		From whom consent was sought (representatives of the cluster, or individual cluster members, or both), and whether consent was sought before or after randomisation	Yes, page 4 and previous articles
**Blinding**	11a	If done, who was blinded after assignment to interventions (for example, participants, care providers, those assessing outcomes) and how		Yes, previous articles
	11b	If relevant, description of the similarity of interventions		Yes, page 4-5 and previous articles
**Statistical methods**	12a	Statistical methods used to compare groups for primary and secondary outcomes	How clustering was taken into account	Yes, page 5-6 and previous articles
	12b	Methods for additional analyses, such as subgroup analyses and adjusted analyses		Yes, page 5-6
**Results**				
**Participant flow (a diagram is strongly recommended)**	13a	For each group, the numbers of participants who were randomly assigned, received intended treatment, and were analysed for the primary outcome	For each group, the numbers of clusters that were randomly assigned, received intended treatment, and were analysed for the primary outcome	No, but flow chart of the randomization can be found in previous papers

For peer review only

For peer review only - http://bmjopen.bmj.com/site/about/guidelines.xhtml

	13b	For each group, losses and exclusions after randomisation, together with reasons	For each group, losses and exclusions for both clusters and individual cluster members	No, see above
**Recruitment**	14a	Dates defining the periods of recruitment and follow-up		Yes, page 4-5
	14b	Why the trial ended or was stopped		Not appropriate
**Baseline data**	15	A table showing baseline demographic and clinical characteristics for each group	Baseline characteristics for the individual and cluster levels as applicable for each group	Yes, table 1
**Numbers analysed**	16	For each group, number of participants (denominator) included in each analysis and whether the analysis was by original assigned groups	For each group, number of clusters included in each analysis	Yes, page 4-5
**Outcomes and estimation**	17a	For each primary and secondary outcome, results for each group, and the estimated effect size and its precision (such as 95% confidence interval)	Results at the individual or cluster level as applicable and a coefficient of intracluster correlation (ICC or k) for each primary outcome	Yes, page 6-7, Suppl. Table and figures
	17b	For binary outcomes, presentation of both absolute and relative effect sizes is recommended		Page 6-7 and tables 2 and Suppl. Table
**Ancillary analyses**	18	Results of any other analyses performed, including subgroup analyses and adjusted analyses, distinguishing pre-specified from exploratory		Yes, this is a sub-group anayses
**Harms**	19	All important harms or unintended effects in each group (for specific guidance see CONSORT for harms[3])		Yes, tables, figures
**Discussion**				
**Limitations**	20	Trial limitations, addressing sources of potential bias,		Yes

For peer review only - http://bmjopen.bmj.com/site/about/guidelines.xhtml

		imprecision, and, if relevant, multiplicity of analyses		
**Generalisability**	21	Generalisability (external validity, applicability) of the trial findings	Generalisability to clusters and/or individual participants (as relevant)	Yes
**Interpretation**	22	Interpretation consistent with results, balancing benefits and harms, and considering other relevant evidence		Yes
**Other information**				
**Registration**	23	Registration number and name of trial registry		Yes, page 2
**Protocol**	24	Where the full trial protocol can be accessed, if available		Yes, page 2
**Funding**	25	Sources of funding and other support (such as supply of drugs), role of funders		Yes

** Note: page numbers optional depending on journal requirements*

For peer review only

BMJ Open

Country of birth, educational level and other predictors of seeking care due to decreased fetal movements – data from a randomized controlled trial in Sweden

Journal:	*BMJ Open*
Manuscript ID	bmjopen-2021-050621.R1
Article Type:	Original research
Date Submitted by the Author:	10-May-2021
Complete List of Authors:	Radestad, Ingela; Sophiahemmet University<br>Pettersson, Karin; Karolinska Institute, Department of Clinical Science, Intervention and Technology<br>Lindgren, Helena; Karolinska Institute, Women's and Children's Health<br>Skokic, Viktor; University of Gothenburg Sahlgrenska Academy, Oncology<br>Akselsson, Anna; Sophiahemmet University, Health Promoting Science
**Primary Subject Heading**:	Obstetrics and gynaecology
Secondary Subject Heading:	Epidemiology
Keywords:	Fetal medicine < OBSTETRICS, Maternal medicine < OBSTETRICS, Prenatal diagnosis < OBSTETRICS

SCHOLARONE™
Manuscripts

I, the Submitting Author has the right to grant and does grant on behalf of all authors of the Work (as defined in the below author licence), an exclusive licence and/or a non-exclusive licence for contributions from authors who are: i) UK Crown employees; ii) where BMJ has agreed a CC-BY licence shall apply, and/or iii) in accordance with the terms applicable for US Federal Government officers or employees acting as part of their official duties; on a worldwide, perpetual, irrevocable, royalty-free basis to BMJ Publishing Group Ltd ("BMJ") its licensees and where the relevant Journal is co-owned by BMJ to the co-owners of the Journal, to publish the Work in this journal and any other BMJ products and to exploit all rights, as set out in our licence.

The Submitting Author accepts and understands that any supply made under these terms is made by BMJ to the Submitting Author unless you are acting as an employee on behalf of your employer or a postgraduate student of an affiliated institution which is paying any applicable article publishing charge ("APC") for Open Access articles. Where the Submitting Author wishes to make the Work available on an Open Access basis (and intends to pay the relevant APC), the terms of reuse of such Open Access shall be governed by a Creative Commons licence – details of these licences and which Creative Commons licence will apply to this Work are set out in our licence referred to above.

Other than as permitted in any relevant BMJ Author's Self Archiving Policies, I confirm this Work has not been accepted for publication elsewhere, is not being considered for publication elsewhere and does not duplicate material already published. I confirm all authors consent to publication of this Work and authorise the granting of this licence.

For peer review only

Country of birth, educational level and other predictors of seeking care due to decreased fetal movements – data from a randomized controlled trial in Sweden

Ingela Rådestad, RNM, Professor, Sophiahemmet University, Stockholm, Sweden.
E-mail: ingela.radestad@shh.se

Karin Pettersson, MD, Associate Professor, Department of Clinical Science, Intervention and Technology, Karolinska Institutet, Stockholm, Sweden. E-mail: karin.ma.pettersson@sll.se

Helena Lindgren, Associate Professor, Department of Women's and children's health, Karolinska Institutet, Stockholm, Sweden. E-mail: Helena.lindgren@ki.se

Viktor Skokic, Statistician, Institute of Clinical Sciences, Sahlgrenska Academy, University of Gothenburg, Gothenburg, Sweden. E-mail: viktor.skokic@gu.se

Anna Akselsson*, RNM, PhD, Department of Health Promoting Science, Sophiahemmet University, Karolinska Institutet, Department of Women's and children's health, Stockholm, Sweden. E-mail: anna.akselsson@shh.se

*Corresponding author
Anna Akselsson, RNM, PhD
Sophiahemmet University
PB 5605, S-114 86, Stockholm, Sweden.
E-mail: anna.akselsson@shh.se
Phone: +46 8 406 20 79

For peer review only

Abstract

Design Observational study with data from a cluster-randomized controlled trial.

Setting 67 maternity clinics and six obstetrical clinics in Sweden.

Participants All pregnant women with a singleton pregnancy who contacted the obstetrical clinic due to decreased fetal movements from 32 weeks' gestation of 39 865 women included in a cluster-randomized controlled trial.

Methods Data were collected from a cluster-randomized controlled trial in where maternity clinics were randomized to Mindfetalness or routine care. Mindfetalness is a self-assessment method for the women to use daily to become familiar with the unborn baby's fetal movement pattern.

Outcome measures Predictors for contacting healthcare due to decreased fetal movements and whether the Mindfetalness method affects women's care-seeking behavior.

Results Overall, 5.2% (n=2059) of women contacted healthcare due to decreased fetal movements, among which 1287 women (62.5%) were registered at a maternity clinic randomized to Mindfetalness and 772 women (37.5%) were randomized to routine care. Predictors for contacting healthcare due to decreased fetal movements were age, country of birth, educational level, parity, prolonged pregnancy and previous psychiatric care ($p<0.001$). The main differences were seen among women born in Africa as compared to Swedish-born women (2.0% vs. 6.0%, RR 0.34, CI 0.25-0.44) and among women with low educational level compared to women with university-level education (2.0% vs. 5.4%, RR 0.36, CI 0.19-0.62). Introducing Mindfetalness in maternity care increased the number of women seeking care due to decreased fetal movements overall.

Conclusion Women with country of birth outside Sweden and low educational level sought care for decreased fetal movements to a lesser extent compared to women born in Sweden and those with university degrees. Future research could explore whether pregnancy outcomes can be improved by motivating women in these groups to contact healthcare if they feel a decreased strength or frequency of fetal movements.

Trial registration www.clinicaltrials.gov (NCT02865759).

For peer review only - http://bmjopen.bmj.com/site/about/guidelines.xhtml

Strengths and limitations of this study

- This is the first study exploring the association between country of birth and contact due to decreased fetal movements.

- The new information about predictors for contacting healthcare due to decreased fetal movements can be valuable for preventing stillbirth.

- Zero lost to follow up, large sample size and population-based register is a methodological strength.

- Misclassified subjects might have affected result of the predictors and it is probably an underestimation of the number of women seeking care.

- Possible confounders, not being considered in the study, could have had an impact of the result.

Introduction

A woman's interpretation of signals from the fetus and her ability to take action will determine whether she contacts healthcare when she has concerns for her unborn baby's health. Her response to altered fetal movement patterns can be decisive for the pregnancy outcomes. Among women who have sought care due to decreased fetal movements, reports indicate that one quarter will later experience adverse baby outcomes, such as low Apgar score, small for gestational age, or stillbirth [1-3]. Further, up to fifty percent of women who experience stillbirth report a gradual decrease in fetal movements several days before the baby died [4,5]. The majority waited more than 24 hours before contacting healthcare without the perception of any movements [6,7]. Contact with healthcare due to decreased fetal movements is common and most of the women return home after normal findings are confirmed. The prevalence of women contacting healthcare with concerns of fetal movements varies internationally; from six to 34 percent [8-11].

Women with normal pregnancies can distinguish between many different types of fetal movements and most feel strong movements [12]. Maternal perception of fetal movements and becoming familiar with the movement pattern is important in reducing stillbirth, as reported in research from New Zealand [13]. The character and diurnal rhythm of movements seem to be essential factors in preventing stillbirth. Maternally perceived increased strength of fetal

For peer review only - http://bmjopen.bmj.com/site/about/guidelines.xhtml

movements is associated with reduced stillbirth rate (aOR 0.21, 95% CI 0.12–0.36) and quiet or light movements in the evening were associated with higher risk (aOR 3.82, 95% CI 1.57–9.31).

Raising maternal awareness of fetal movements by using the Mindfetalness method may be a way forward for women to become familiar with the unborn baby's movement pattern [14]. Compared to counting methods [15,16], Mindfetalness instruct the women to observe the variation of movements. Practicing the method includes to lie down for 15 minutes a day from 28 weeks' gestation, when the baby is awake and to focus on the character, strength and frequency of the unborn baby's movements, without counting each movement. Mindfetalness was invented in 2012 [14] and has been evaluated by women and midwives in two studies [17,18]. Firstly, the women tested Mindfetalness once, and count-to-ten once. The women appreciated both methods but preferred Mindfetalness if they had to choose. Secondly, information about Mindfetalness were distributed to 104 pregnant women in maternity care and the majority liked the method and 75 percent practiced the method. Additionally, in a cluster randomized controlled trial, 67 maternity clinics were allocated to either Mindfetalness or routine care [19,20]. Midwives in the Mindfetalness group were instructed to distribute oral and written information about fetal movements and about Mindfetalness. Information regarding the method was available in nine different languages and a website was open for anyone to access. The trial showed several benefits of introducing Mindfetalness into maternity care [19]. In the Mindfetalness group, more women started their labor spontaneously, followed by a reduction in labor inductions and cesarean sections. Further, less babies were born small for gestational age and needed neonatal care.

The ambition to encourage women to seek care if they have concerns of their unborn baby's fetal movements pattern is to reduce the stillbirth rate without too much overload on both women and caregivers. However, stillbirth is a very rare phenomenon and thus comparing stillbirth rate in this study is impossible. In this study, we investigate predictors of seeking care for decreased fetal movements and whether care-seeking behavior was influenced by a method (Mindfetalness) aimed at increasing pregnant women's knowledge of their unborn baby's fetal movement patterns [14]. We further investigate pregnancy outcomes for women seeking care for decreased fetal movements but where, at the time of the examination, no interventions such as induction of labor or caesarean section were made.

For peer review only - http://bmjopen.bmj.com/site/about/guidelines.xhtml

Methods

Pregnant women with a singleton pregnancy who contacted healthcare due to decreased fetal movements from 32 weeks' gestation constitute the population of this study. Data were collected from a cluster-randomized controlled trial in where 67 maternity clinics in Stockholm, Sweden, were randomized to either intervention with Mindfetalness or routine care, as reported previously [19,20]. The women who contacted healthcare due to decreased fetal movements from 32 weeks' gestation were generated from the ICD-code AM041 (examination due to decreased fetal movements) [21]. The code is a diagnosis for pregnant women who have contacted healthcare due to decreased fetal movements, where no interventions, such as labor induction or cesarean section, were made, at the time the woman sought care.

The randomization resulted in 33 maternity clinics in the Mindfetalness group and 34 in routine care group. After the randomization, one clinic randomized to Mindfetalness declined participation. We applied intention-to-treat design; extended information about the randomization process can be found in previous publications [19,20]. The Mindfetalness group included 19 639 women and the routine care group 20 226 women.

Starting in October 2016, midwives, working at maternity clinics randomized to the intervention, distributed a leaflet to pregnant women, including information about fetal movements and the self-assessment method, Mindfetalness. The last leaflet was distributed on 31 January 2018, and the women were followed-up until birth in population-based registers. Midwives in the routine care group followed standard care, which includes providing oral information about fetal movements at 24 weeks' gestation.

The Mindfetalness leaflet (appendix) included information about fetal movements and when to contact healthcare; "If you are concerned that the fetus is moving less or that the movements are weaker, you should contact healthcare." Further, the leaflet included an invitation to practice Mindfetalness daily from 28 weeks' gestation. The women are instructed to lie down on the left side 15 minutes a day, when the baby is awake, and focus on the intensity, character and frequency of the movements, without counting each movement. The information was available in nine different languages and the website www.mindfetalness.com was open for anyone to access.

For peer review only - http://bmjopen.bmj.com/site/about/guidelines.xhtml

This study was approved by the Regional Ethics committee in Stockholm, Sweden (Dnr 2015/2105–31/1), 13 January 2016.

Statistical analysis

We identified the background factors of the women who contacted healthcare due to decreased fetal movements by calculating percentages. The included variables were age, country of birth, education level, parity, assisted reproduction, previous stillbirth, tobacco use at registration in maternity clinic, body mass index, and maternal diseases. To calculate predictors for contacting healthcare, we investigated which factors were statistically significantly associated with contact due to decreased fetal movements. As a metric for association, we calculated relative risks and 95% confidence intervals using log-binomial regression. In the predictor calculation, we adjusted for the possible confounding factor, parity. For outcome measures, we calculated percentage and compared the Mindfetalness group with the routine care group using log-binomial regression. We considered a *p*-value of 0.05 to be statistically significant. We used the statistical program R (version 3.2.4). Loss to follow-up was zero due to the population-based Swedish Pregnancy Register [22] used for data collection.

Results

Among all women in this study, 5.2% (*n*=2059) contacted healthcare due to decreased fetal movements, measured from gestational week 32 until birth. As shown in Figure 1, 62.5% (*n*=1287) were registered at a maternity clinic randomized to Mindfetalness, and 37.5% (*n*=772) to routine care. The background data for the women are displayed in Table 1.

Table 1. Characteristics among women with a singleton pregnancy from 32 weeks' gestation contacting healthcare versus not contacting healthcare due to decreased fetal movements.

Characteristics	Contacted healthcare due to decreased fetal movements (*n*=2059) *n* (%)	Did not contact healthcare due to decreased fetal movements (*n*=37 806) *n* (%)	Relative risk (CI)	*p*-value
**Age***				
≤24 years	187 (9.1)	2628 (7.0)	1.31 (1.13–1.51)	<0.001
25–34 years	1361 (66.1)	24 137 (63.8)	1.04 (1.00–1.07)	0.04
≥35 years	511 (24.8)	11 041 (29.2)	0.85 (0.79–0.92)	<0.001
**Country of birth**				

For peer review only - http://bmjopen.bmj.com/site/about/guidelines.xhtml

Sweden	1595 (77.5)	24 890 (65.8)	1.18 (1.15–1.21)	<0.001
Europe (except Sweden)	137 (6.7)	3780 (10.0)	0.67 (0.56–0.79)	<0.001
Africa	51 (2.5)	2450 (6.5)	0.38 (0.29–0.50)	<0.001
Asia	224 (10.9)	5663 (15.0)	0.73 (0.64–0.82)	<0.001
North America	16 (0.8)	304 (0.8)	0.97 (0.59–1.60)	1.00
South America	31 (1.5)	622 (1.6)	0.92 (0.64–1.31)	0.72
Others	5 (0.2)	97 (0.3)	0.95 (0.39–2.33)	1.00
**Education level¶**				
Shorter than 9 years	11 (0.5)	551 (1.5)	0.36 (0.20–0.66)	<0.001
Elementary school	69 (3.4)	1734 (4.6)	0.72 (0.57–0.91)	0.006
High school	546 (26.5)	9717 (25.7)	1.02 (0.95–1.10)	0.64
University	1290 (62.7)	22 714 (60.1)	1.03 (1.00–1.06)	0.09
**Parity §**				
Primipara	1127 (54.7)	16 344 (43.2)	1.26 (1.21–1.32)	<0.001
Multipara	930 (45.2)	21 324 (56.4)	0.80 (0.76–0.84)	<0.001
**Assisted reproduction§**	129 (6.3)	2033 (5.4)	1.17 (0.98–1.38)	0.09
**Previous stillbirth**	9 (0.4)	211 (0.6)	0.78 (0.40–1.52)	0.64
**Tobacco user at registration in the maternity clinic (including snuff) §**	79 (3.8)	1256 (3.3)	1.16 (0.92–1.44)	0.21
**Body Mass Index∫**				
<18.5 kg/m²	54 (2.6)	1021 (2.7)	0.97 (0.74–1.27)	0.89
18.5–24.9 kg/m²	1228 (59.6)	22 624 (59.8)	0.99 (0.96–1.03)	0.70
25.0–29.9 kg/m²	464 (22.5)	8611 (22.8)	0.99 (0.91–1.07)	0.74
30.0–34.9 kg/m²	167 (8.1)	2889 (7.6)	1.06 (0.91–1.23)	0.47
≥35.0 kg/m²	63 (3.1)	1000 (2.6)	1.15 (0.90–1.48)	0.26
**Maternal diseases**				
Diabetes mellitus	1 (0.0)	63 (0.2)	0.29 (0.04–2.10)	0.26
Coronary heart disease	25 (1.2)	600 (1.6)	0.77 (0.51–1.14)	0.20
Thrombosis	13 (0.6)	283 (0.7)	0.84 (0.49–1.47)	0.69
Systemic lupus erythematosus	1 (0.0)	58 (0.2)	0.32 (0.04–2.28)	0.37
Psychiatric care	354 (17.2)	4744 (12.5)	1.37 (1.24–1.51)	<0.001
Endocrine disease	156 (7.6)	2633 (7.0)	1.09 (0.93–1.27)	0.29
Epilepsy	10 (0.5)	167 (0.4)	1.10 (0.58–2.08)	0.73
Chronic hypertension	9 (0.4)	184 (0.5)	0.90 (0.46–1.75)	0.87
Other disease	204 (9.9)	3583 (9.5)	1.05 (0.91–1.20)	0.51
Medication or psychologic treatment for mental illness	129 (6.3)	2058 (5.4)	1.15 (0.97–1.37)	0.11

* Mean age of women who contacted care=31.2, who did not contact care=32.4 (p<0.001)
¶ Data missing for women who contacted care n=143 (6.9%), who did not contact care n=3090 (8.2%)
§ Data missing for women who contacted care n=2 (0.1%), who did not contact care n=138 (0.4%)
∫ Data missing for women who contacted care n=83 (4.0%), who did not contact care n=1661 (4.4%)

Predictors for seeking care for decreased fetal movements were age, country of birth, educational level, parity, prolonged pregnancy and previous psychiatric care (p<0.001) (Table 2). The main differences were seen in country of birth and educational levels. Women born in Africa sought care to a lower extent as compared to women born in Sweden, 2.0% versus 6.0% (RR 0.34, CI 0.25–0.44).

Table 2. Predictors for contacting healthcare due to decreased fetal movements

For peer review only

Predictor	*p-value*	*n (%)*	*RR (CI)*	*p-value*	*AdjRR (CI)**
Age	<0.001			0.02	
≤24 years		187 (6.6)	1.24 (1.07–1.44)		1.15 (0.98–1.33)
25–34 years		1361 (5.3)	1.0 (Reference)		1.0 (Reference)
≥35 years		511 (4.4)	0.83 (0.75–0.91)		0.90 (0.82–1.00)
Country of birth	<0.001			<0.001	
Sweden		1595 (6.0)	1.0 (Reference)		1.0 (Reference)
Europe (except Sweden)		137 (3.5)	0.58 (0.49–0.69)		0.58 (0.49 – 0.69)
Africa		51 (2.0)	0.34 (0.25–0.44)		0.36 (0.27–0.47)
Asia		224 (3.8)	0.63 (0.55–0.72)		0.66 (0.57–0.75)
North America		16 (5.0)	0.83 (0.49–1.29)		0.83 (0.49–1.28)
South America		31 (4.7)	0.79 (0.54–1.09)		0.82 (0.56–1.13)
Others		5 (4.9)	0.81 (0.30–1.70)		0.84 (0.31–1.75)
Marital status	0.20			0.06	
Cohabiting with becoming father		1914 (5.2)	1.0 (Reference)		1.0 (Reference)
Single		29 (4)	0.76 (0.52–1.07)		0.73 (0.49–1.02)
Other family situation		114 (4.8)	0.92 (0.76–1.10)		0.87 (0.72–1.04)
Education level	<0.001			<0.001	
Shorter than 9 years		11 (2.0)	0.36 (0.19–0.62)		0.41 (0.21–0.69)
Elementary school		69 (3.8)	0.71 (0.56–0.90)		0.75 (0.58–0.94)
High school		546 (5.3)	0.99 (0.90–1.09)		1.00 (0.91–1.10)
University		1290 (5.4)	1.0 (Reference)		1.0 (Reference)
Parity	<0.001			NA	
Primipara		1127 (6.5)	1.54 (1.42–1.68)		NA
Multipara		930 (4.2)	1.0 (Reference)		NA
Gestation week >41+6	<0.001			0.01	
No			1.0 (Reference)		1.0 (Reference)
Yes			1.34 (1.13–1.56)		1.25 (1.06–1.46)
Assisted reproduction	0.10			0.55	
No		1928 (5.1)	1.0 (Reference)		1.0 (Reference)
Yes		129 (6.0)	1.16 (0.97–1.38)		1.05 (0.88–1.25)
Previous stillbirth	0.46			0.95	
No		2050 (5.2)	1.0 (Reference)		1.0 (Reference)
Yes		9 (4.1)	0.79 (0.38–1.41)		0.98 (0.47–1.74)
Tobacco user at registration at the maternity clinic§	0.22			0.17	
No			1.0 (Reference)		1.0 (Reference)
Yes		79 (5.9)	1.15 (0.92–1.42)		1.17 (0.93–1.44)
Body Mass Index	0.77			0.37	
<18.5 kg/m²		54 (5.0)	0.98 (0.74–1.26)		0.95 (0.72–1.23)
18.5–24.9 kg/m²		1228 (5.1)	1.0 (Reference)		1.0 (Reference)
25.0–29.9 kg/m²		464 (5.1)	0.99 (0.89–1.10)		1.03 (0.92–1.14)
30.0–34.9 kg/m²		167 (5.5)	1.06 (0.90–1.24)		1.12 (0.95–1.31)
≥35.0 kg/m²		63 (5.9)	1.15 (0.89–1.46)		1.22 (0.94–1.54)
Psychiatric care	<0.001			<0.001	
No		1705 (4.9)	1.0 (Reference)		1.0 (Reference)
Yes		354 (6.9)	1.42 (1.27–1.58)		1.37 (1.22–1.52)
Medication or psychologic treatment for mental illness	0.12			0.13	
No		1930 (5.1)	1.0 (Reference)		1.0 (Reference)
Yes		129 (5.9)	1.15 (0.96–1.36)		1.15 (0.96–1.36)

*Adjusted for parity

For peer review only

For peer review only - http://bmjopen.bmj.com/site/about/guidelines.xhtml

Further, women with lowest educational levels sought care due to decreased fetal movements to a lower extent compared to women with university-level education (2.0% versus 5.4%, RR 0.36, CI 0.19–0.62). Additionally, primiparas, women with prolonged pregnancies (>41+6 gestation weeks), and women with experience of psychiatric care more often sought care due to decreased fetal movements (Table 2). The predictors for contacting healthcare due to decreased fetal movements remain after adjustment for parity. Mindfetalness increased the prevalence of women contacting healthcare due to decreased fetal movements overall (6.6% versus 3.8%, RR 1.72, CI 1.57–1.87) (not in table). When stratifying women into groups according to background factors, the percentage of women contacting healthcare increased in all groups (Supplementray Table 1).

Figures 2a and 2b show the pregnancy outcomes for women contacting healthcare due to decreased fetal movements, stratified for women registered at maternity clinics randomized to Mindfetalness and routine care, respectively. Compared to routine care, women in the Mindfetalness group had a higher percentage of women with spontaneous start of labor (66.4% versus 63.0%) and a lower rate of labor induction (25.6% versus 28.6%). The proportion of women who had onset of labor from 42 weeks' gestation was 6.8% in the Mindfetalness group, and 7.8% in routine care. Further, a lower proportion of babies in the Mindfetalness group were in need of transfer to NICU (6.3% versus 8.0%). One stillbirth was observed, in the routine care group (not in table). There were no statistically significant differences in the investigated pregnancy outcomes in Figures 2a and 2b, between the Mindfetalness and routine care groups. For further information in pregnancy outcomes for Mindfetalness and routine care, see Supplementary Table 2.

Discussion

During the studied period, in the Stockholm region, women born outside Sweden and women with low levels of education sought care for decreased fetal movements to a lower extent when compared to women born in Sweden and women with university-level education. Further, women of higher age contacted healthcare due to decreased fetal movements to a lower extent than women of a younger age. Country of birth outside Sweden, low educational level and advanced maternal age have previously been related to adverse birth outcomes. Introducing Mindfetalness

For peer review only - http://bmjopen.bmj.com/site/about/guidelines.xhtml

in maternity care increased the number of women contacting healthcare due to decreased fetal movements.

We found that women born outside Sweden (mostly from Africa and Asia) sought care due to decreased fetal movements to a lower extent. No studies, to our knowledge, have investigated the association between contact with healthcare due to maternal concern of fetal movements and country of birth. However, previous research has reported a higher risk of stillbirth among women not being born, but giving birth, in a Western country [23-28]. Suggested mechanisms for this difference include sub-standard maternity care, lack of communication between the pregnant woman and healthcare professionals, and variations in pre-pregnancy health [24,29]. It is possible that the higher risk for stillbirth can be linked to lower awareness and knowledge about fetal movements and when to contact healthcare [6,7].

Giving information to pregnant women about fetal movements may reduce pre-hospital delays [30-32] and has been suggested to reduce the stillbirth rate in an intervention study performed in Norway[32]. The study documented a 50 percent reduction in the stillbirth rate among women who contacted healthcare due to decreased fetal movements as compared to women who did not. The intervention included providing health promotion information to the pregnant women, an invitation to count fetal movements, and information that they should never wait until the next day to contact healthcare if they had concerns about fetal movements. Additionally, providing new guidelines to the healthcare professionals was included in the intervention. A reduction in patient delay and increased knowledge about fetal movements was shown in a recent study from the Netherlands after the introduction of a leaflet about fetal movements [33]. However, in the AFFIRM-trial[34], women in the intervention arm (including information to pregnant women and new guidelines to the healthcare professionals) had lower rate of stillbirths than women in the control group, but the difference was not statistically significant. Labor inductions and cesarean section increased, and the number of babies in need of neonatal care more than 48 hours.

The midwife is the most important source of information [35,36] and The Swedish National Board of Health and Welfare recently introduced new guidelines for maternity care, including instructions to midwives to inform pregnant women about fetal movements [37]. The guidelines have good compliance among midwives (79-87% answered yes to the different questions whether they followed the guidelines), but some report barriers due to lack of time, high workload and

For peer review only - http://bmjopen.bmj.com/site/about/guidelines.xhtml

problems in communicating with women born outside of Sweden [38]. Suboptimal care is more common among non-western women and action to improve perinatal mortality for this group is needed [24].

In this study, women with low education levels sought care due to decreased fetal movements to a lower extent, as compared to women with high education levels. A higher risk for stillbirth has been found among women with low educational levels and low-socioeconomic status [6,39-41]. There are reasons to believe that raising awareness about fetal movements among women with low education or socioeconomic status would increase the number of women seeking care due to decreased fetal movements, which in turn would possibly decrease perinatal mortality.

Women of advanced maternal age contacted healthcare due to decreased fetal movements to a lower extent than younger women in our study, and a higher risk of stillbirth is reported among women aged from 35 years compared to younger women [42]. The mean age in an Israeli study was lower among women who had contacted healthcare due to decreased fetal movements compared to the reference group [43]. In a study from Norway, the percentage of women over 35 years of age was lower among women who contacted healthcare due to decreased fetal movements compared to the reference group (16% versus 20%) [3]. However, in a recent systematic review, the authors interpreted current evidence as not supporting the notion that a woman's mean age influences whether she seeks care for decreased fetal movements [44].

Introducing Mindfetalness in maternity care increased the number of women contacting healthcare due to decreased fetal movements overall. Mindfetalness possibly increases the woman's ability to become familiar with the unborn baby's fetal movement patterns. Reducing pre-hospital delay when the perception that fetal movements are reduced is suggested to be one way for improving perinatal mortality [7,30,31].

Methodological considerations

The prevalence of women seeking care due to decreased or altered fetal movements in our study is low compared to the figures from other research. In a study performed in Sweden in 2014, 9.3 percent of the women sought care due to decreased fetal movements when measured from gestational week 28 until birth [9]. Further, international studies show a prevalence of 6–34 percent [8,10,11,45]. We believe that the figures in our study are underestimated, mostly due to the fact that

For peer review only - http://bmjopen.bmj.com/site/about/guidelines.xhtml

we only included women who sought care from gestational week 32. Additionally, the ICD-code AM041 is not the best tool to use for including all women who contact healthcare due to decreased fetal movements, but it is the only code available. Misclassification in the study may be important to consider as some women who sought care due to decreased fetal movements didn't get the ICD-code and were classified as they had not sought care. This might have diluted the differences between the groups of women who sought/not sought care due to decreased fetal movements. However, when comparing the Mindfetalness group with routine care group, only non-differential misclassification is likable, which effect the differences in the same way in the two groups.

The only potential confounder we adjusted for was parity. We do not have information about other possibly confounders, for example, if some of the women had extra appointments in specialist maternity clinics, which may have affected their care-seeking behavior. Extra pre-booked meetings with midwives or obstetricians and investigations with cardiotocography or ultrasound due to other complication can also decrease the need for unscheduled visits at the hospitals.

When evaluating whether Mindfetalness has an effect on women contacting healthcare due to decreased fetal movements, the random allocation, the large sample size, and zero lost to follow-up are strengths of the study. Further, the missing data are negligible, thanks to the unique population-based register in Sweden. While the intention-to-treat design does preserve the advantages of random allocation, it also risks misclassification and the dilution of the results, i.e., it is an underestimation of the true effects. From the pilot study, we know that approximately 75 percent of the Swedish-speaking women use the method [17], and, from the main trial, about 22 percent did not receive a leaflet [19]. Additionally, the information was available in nine languages and, in Stockholm, Sweden, there is a large variety of women who originate from different countries and who speak languages that were not included in the intervention and this may have diluted the effect. This further contributes to the possible dilution of effects.

We can only study predictors for seeking care for decreased fetal movements and less likely to seek care were i) aged women, ii) less educated, and iii) non-Swedish women. In the Mindfetalness-group, effects of the intervention affect the results. Women using Mindfetalness were more likely to visit healthcare-providers due to decreased fetal movements

For peer review only - http://bmjopen.bmj.com/site/about/guidelines.xhtml

than women in the control-group. We believe that Mindfetalness may have reduced the fraction of women of no-visit of i) aged women, ii) less educated, and iii) non-Swedish women. However we cannot, in this study draw conclusions that we would not had de same results if we had used conventional count-ten in our study.

Implications

The National Board of Health and Welfare claim that pregnant women should receive information about risk factors for adverse pregnancy outcomes, including stillbirth [38]. Women born in low-income countries and women with advanced maternal age are factors to consider when trying to improve perinatal mortality in high-income countries [42,46]. Providing targeted information, available in a variety of languages, to these women about fetal movements and when to contact healthcare might help to improve perinatal outcomes. Also, providing information about how to practice Mindfetalness can be one way forward to reduce adverse pregnancy outcomes. Today, we have a great number of opportunities to reach pregnant women thanks to the internet and to target women in at-risk groups. Additionally, this information can be provided in different forms, for example, videos, apps and interactive webpages.

Conclusion

We do not know for certain what the ideal frequency is for women seeking care due to decreased fetal movements, but we can conclude that it is lower in women who have migrated to Sweden from, for example, Africa and Asia, and in women with low educational levels. Women from low-income countries have documented worse pregnancy outcomes. An implication of our results may be to examine whether pregnancy outcomes can be improved by providing customized information to women from low-income countries with the goal that they seek care if they feel a decreased strength or frequency of fetal movements. More data are needed to draw conclusions about the effects of Mindfetalness in perinatal mortality among women contacting healthcare due to decreased fetal movements.

Acknowledgements

The authors would like to thank the midwives and the pregnant women in the maternity clinics in Stockholm. Thanks to the coordination midwives and medical doctors at

For peer review only - http://bmjopen.bmj.com/site/about/guidelines.xhtml

Mödrahälsovårdsenheten and to The Swedish Pregnancy Register for cooperation. Special thanks to The Swedish Research Council for funding this study.

References

1. Dutton PJ, Warrander LK, Roberts SA, et al. Predictors of poor perinatal outcome following maternal perception of reduced fetal movements--a prospective cohort study. *PLoS One* 2012; **7**(7): e39784.
2. Higgins LE, Myers JE, Sibley CP, Johnstone ED, Heazell AEP. Antenatal placental assessment in the prediction of adverse pregnancy outcome after reduced fetal movement. *PLoS One* 2018; **13**(11): e0206533.
3. Holm Tveit JV, Saastad E, Stray-Pedersen B, Bordahl PE, Froen JF. Maternal characteristics and pregnancy outcomes in women presenting with decreased fetal movements in late pregnancy. *Acta obstetricia et gynecologica Scandinavica* 2009; **88**(12): 1345-51.
4. Linde A, Pettersson K, Radestad I. Women's Experiences of Fetal Movements before the Confirmation of Fetal DeathContractions Misinterpreted as Fetal Movement. *Birth-Issues in Perinatal Care* 2015; **42**(2): 189-94.
5. Warland J, O'Brien LM, Heazell AE, Mitchell EA. An international internet survey of the experiences of 1,714 mothers with a late stillbirth: the STARS cohort study. *BMC pregnancy and childbirth* 2015; **15**: 172.
6. Froen JF, Arnestad M, Frey K, Vege A, Saugstad OD, Stray-Pedersen B. Risk factors for sudden intrauterine unexplained death: epidemiologic characteristics of singleton cases in Oslo, Norway, 1986-1995. *American journal of obstetrics and gynecology* 2001; **184**(4): 694-702.
7. Koshida S, Ono T, Tsuji S, Murakami T, Arima H, Takahashi K. Excessively delayed maternal reaction after their perception of decreased fetal movements in stillbirths: Population-based study in Japan. *Women and birth : journal of the Australian College of Midwives* 2017.
8. Kapaya H, Almeida J, Karouni F, Anumba D. Management of reduced fetal movement: A comparative analysis of two audits at a tertiary care clinical service. *Eur J Obstet Gynecol Reprod Biol* 2020; **248**: 128-32.
9. Akselsson A, Lindgren H, Georgsson S, Pettersson K, Radestad I. Increased labor induction and women presenting with decreased or altered fetal movements - a population-based survey. *PLoS One* 2019; **14**(5): e0216216.
10. Froen JF. A kick from within--fetal movement counting and the cancelled progress in antenatal care. *J Perinat Med* 2004; **32**(1): 13-24.
11. Sergent F, Lefevre A, Verspyck E, Marpeau L. [Decreased fetal movements in the third trimester: what to do?]. *Gynecologie, obstetrique & fertilite* 2005; **33**(11): 861-9.
12. Radestad I, Lindgren H. Women's perceptions of fetal movements in full-term pregnancy. *Sexual & reproductive healthcare : official journal of the Swedish Association of Midwives* 2012; **3**(3): 113-6.
13. Bradford BF, Cronin RS, McCowan LME, McKinlay CJD, Mitchell EA, Thompson JMD. Association between maternally perceived quality and pattern of fetal movements and late stillbirth. *Scientific reports* 2019; **9**(1): 9815.
14. Radestad I. Strengthening mindfetalness. *Sexual & reproductive healthcare : official journal of the Swedish Association of Midwives* 2012; **3**(2): 59-60.

For peer review only - http://bmjopen.bmj.com/site/about/guidelines.xhtml

15. Pearson JF. Fetal movements--a new approach to antenatal care. *Nurs Mirror Midwives J* 1977; **144**(16): 49-51.

16. Sadovsky E, Yaffe H. Daily fetal movement recording and fetal prognosis. *Obstetrics and gynecology* 1973; **41**(6): 845-50.

17. Akselsson A, Georgsson S, Lindgren H, Pettersson K, Radestad I. Women's attitudes, experiences and compliance concerning the use of Mindfetalness- a method for systematic observation of fetal movements in late pregnancy. *BMC pregnancy and childbirth* 2017; **17**(1): 359.

18. Malm MC, Radestad I, Rubertsson C, Hildingsson I, Lindgren H. Women's experiences of two different self-assessment methods for monitoring fetal movements in full-term pregnancy--a crossover trial. *BMC pregnancy and childbirth* 2014; **14**: 349.

19. Akselsson A, Lindgren H, Georgsson S, et al. Mindfetalness to increase women's awareness of fetal movements and pregnancy outcomes: a cluster-randomised controlled trial including 39 865 women. *BJOG : an international journal of obstetrics and gynaecology* 2020.

20. Radestad I, Akselsson A, Georgsson S, Lindgren H, Pettersson K, Steineck G. Rationale, study protocol and the cluster randomization process in a controlled trial including 40,000 women investigating the effects of mindfetalness. *Sexual & reproductive healthcare : official journal of the Swedish Association of Midwives* 2016; **10**: 56-61.

21. AB IiG. ICD internetmedicin. 2018. http://icd.internetmedicin.se/ (accessed December 2018).

22. Graviditetsregistret. Graviditetsregistret. https://www.medscinet.com/gr/engelska.aspx (accessed January 2021).

23. Gagnon AJ, Zimbeck M, Zeitlin J, et al. Migration to western industrialised countries and perinatal health: a systematic review. *Soc Sci Med* 2009; **69**(6): 934-46.

24. Saastad E, Vangen S, Froen JF. Suboptimal care in stillbirths - a retrospective audit study. *Acta obstetricia et gynecologica Scandinavica* 2007; **86**(4): 444-50.

25. Khalil A, Rezende J, Akolekar R, Syngelaki A, Nicolaides KH. Maternal racial origin and adverse pregnancy outcome: a cohort study. *Ultrasound Obstet Gynecol* 2013; **41**(3): 278-85.

26. Ravelli AC, Tromp M, Eskes M, et al. Ethnic differences in stillbirth and early neonatal mortality in The Netherlands. *J Epidemiol Community Health* 2011; **65**(8): 696-701.

27. Drysdale H, Ranasinha S, Kendall A, Knight M, Wallace EM. Ethnicity and the risk of late-pregnancy stillbirth. *Med J Aust* 2012; **197**(5): 278-81.

28. Vik ES, Aasheim V, Schytt E, Small R, Moster D, Nilsen RM. Stillbirth in relation to maternal country of birth and other migration related factors: a population-based study in Norway. *BMC pregnancy and childbirth* 2019; **19**(1): 5.

29. Essen B, Bodker B, Sjoberg NO, et al. Are some perinatal deaths in immigrant groups linked to suboptimal perinatal care services? *BJOG : an international journal of obstetrics and gynaecology* 2002; **109**(6): 677-82.

30. Koshida S, Ono T, Tsuji S, Murakami T, Takahashi K. Recommendations for preventing stillbirth: a regional population-based study in Japan during 2007-2011. *Tohoku J Exp Med* 2015; **235**(2): 145-9.

31. RCOG. Reduced Fetal Movements. 2011 https://www.rcog.org.uk/en/guidelines-research-services/guidelines/gtg57/57).

32. Tveit JV, Saastad E, Stray-Pedersen B, et al. Reduction of late stillbirth with the introduction of fetal movement information and guidelines - a clinical quality improvement. *BMC pregnancy and childbirth* 2009; **9**: 32.

For peer review only - http://bmjopen.bmj.com/site/about/guidelines.xhtml

33. Wackers K, Wassen M, Zeegers B, Bude L, Nieuwenhuijze MJ. Effect of the use of a national information brochure about fetal movements on patient delay. *Women and birth : journal of the Australian College of Midwives* 2019; **32**(2): 131-6.
34. Norman JE, Heazell AEP, Rodriguez A, et al. Awareness of fetal movements and care package to reduce fetal mortality (AFFIRM): a stepped wedge, cluster-randomised trial. *Lancet* 2018.
35. Grimes HA, Forster DA, Newton MS. Sources of information used by women during pregnancy to meet their information needs. *Midwifery* 2014; **30**(1): e26-33.
36. McArdle A, Flenady V, Toohill J, Gamble J, Creedy D. How pregnant women learn about foetal movements: sources and preferences for information. *Women and birth : journal of the Australian College of Midwives* 2015; **28**(1): 54-9.
37. Socialstyrelsen. Minskade fosterrörelser – rekommendationer om information, råd och en inledande bedömning – Kunskapsstöd med nationella rekommendationer. 2016. http://www.socialstyrelsen.se/publikationer2016/2016-10-9 (accessed 0407 2020).
38. Socialstyrelsen. Dödfödda barn - En inventering och förslag på åtgärder. 2018. https://www.socialstyrelsen.se/publikationer2018/2018-12-36/Sidor/default.aspx (accessed 01-28 2019).
39. Stephansson O, Dickman PW, Johansson AL, Cnattingius S. The influence of socioeconomic status on stillbirth risk in Sweden. *Int J Epidemiol* 2001; **30**(6): 1296-301.
40. Zeitlin J, Mortensen L, Prunet C, et al. Socioeconomic inequalities in stillbirth rates in Europe: measuring the gap using routine data from the Euro-Peristat Project. *BMC pregnancy and childbirth* 2016; **16**: 15.
41. Socialstyrelsen. Socioekonomiska faktorers påverkan på kvinnors och barns hälsa efter förlossning 2016. file:///H:/Artiklar/Socialstyrelsen/Socioekonomiska%20faktorer%20risk%20negativt%20utfall.pdf (accessed April 2021).
42. Flenady V, Middleton P, Smith GC, et al. Stillbirths: the way forward in high-income countries. *Lancet* 2011; **377**(9778): 1703-17.
43. Levy M, Kovo M, Barda G, et al. Reduced fetal movements at term, low-risk pregnancies: is it associated with adverse pregnancy outcomes? Ten years of experience from a single tertiary center. *Archives of gynecology and obstetrics* 2020; **301**(4): 987-93.
44. Carroll L, Gallagher L, Smith V. Risk factors for reduced fetal movements in pregnancy: A systematic review and meta-analysis. *Eur J Obstet Gynecol Reprod Biol* 2019; **243**: 72-82.
45. Moore TR, Piacquadio K. A prospective evaluation of fetal movement screening to reduce the incidence of antepartum fetal death. *American journal of obstetrics and gynecology* 1989; **160**(5 Pt 1): 1075-80.
46. Flenady V, Koopmans L, Middleton P, et al. Major risk factors for stillbirth in high-income countries: a systematic review and meta-analysis. *Lancet* 2011; **377**(9774): 1331-40.

For peer review only - http://bmjopen.bmj.com/site/about/guidelines.xhtml

Author contributions

IR, KP, HL and AA were involved in the design and planning of the cluster-randomized controlled trial and IR obtained funding. AA had overall responsibility for the study. VS and AA analysed the data with input from IR, KP and HL. AA wrote the original manuscript and all authors made critical input. All authors were responsible for drafting and validating the following versions of the manuscript. All authors approved the final version of the manuscript.

Funding: This study was funded by The Swedish Research Council. Grant number not applicable.

Competing interests: The authors declare that they have no competing interests and there are not any financial competing interests.

Patient consent: Not required.

Ethics approval: This study was approved by the Regional Ethics committee in Stockholm, Sweden (Dnr 2015/2105–31/1), 13 January 2016.

Data sharing statement No additional data is available. Anonymised data are available after ethical approval by the Regional Ethics committee in Stockholm, Sweden and after request to The Swedish Pregnancy Register.

Patient and Public Involvement: Patients or the public were not involved in the design, or conduct, or reporting, or dissemination plans of our research.

Figure 1. Flow chart.

Figure 2a. Birth outcome among women who contacted healthcare due to decreased fetal movements from 32 weeks' gestation, where the examination did not indicate that the pregnancy needed to be terminated, at the time the women sought care.

For peer review only

Figure 2b. Start of labor among women who contacted healthcare due to decreased fetal movements from 32 weeks' gestation, where the examination did not indicate that the pregnancy needed to be terminated, at the time the women sought care.

Supplementary Table 1. Percentage of women who sought care due to decreased fetal movements in Mindfetalness (*n*=19 639) versus routine care (*n*=20 226), divided into characteristics and a calculation of absolute differences between the groups.

Supplementary Table 2. Obstetric outcome from gestation week 32+0 among women with singleton pregnancy who have contacted healthcare due to decreased fetal movements; 1287 registered at a maternity clinic randomized to Mindfetalness, and 772 registered at a maternity clinic randomized to routine care.

For peer review only

Figure 1. Flow chart.

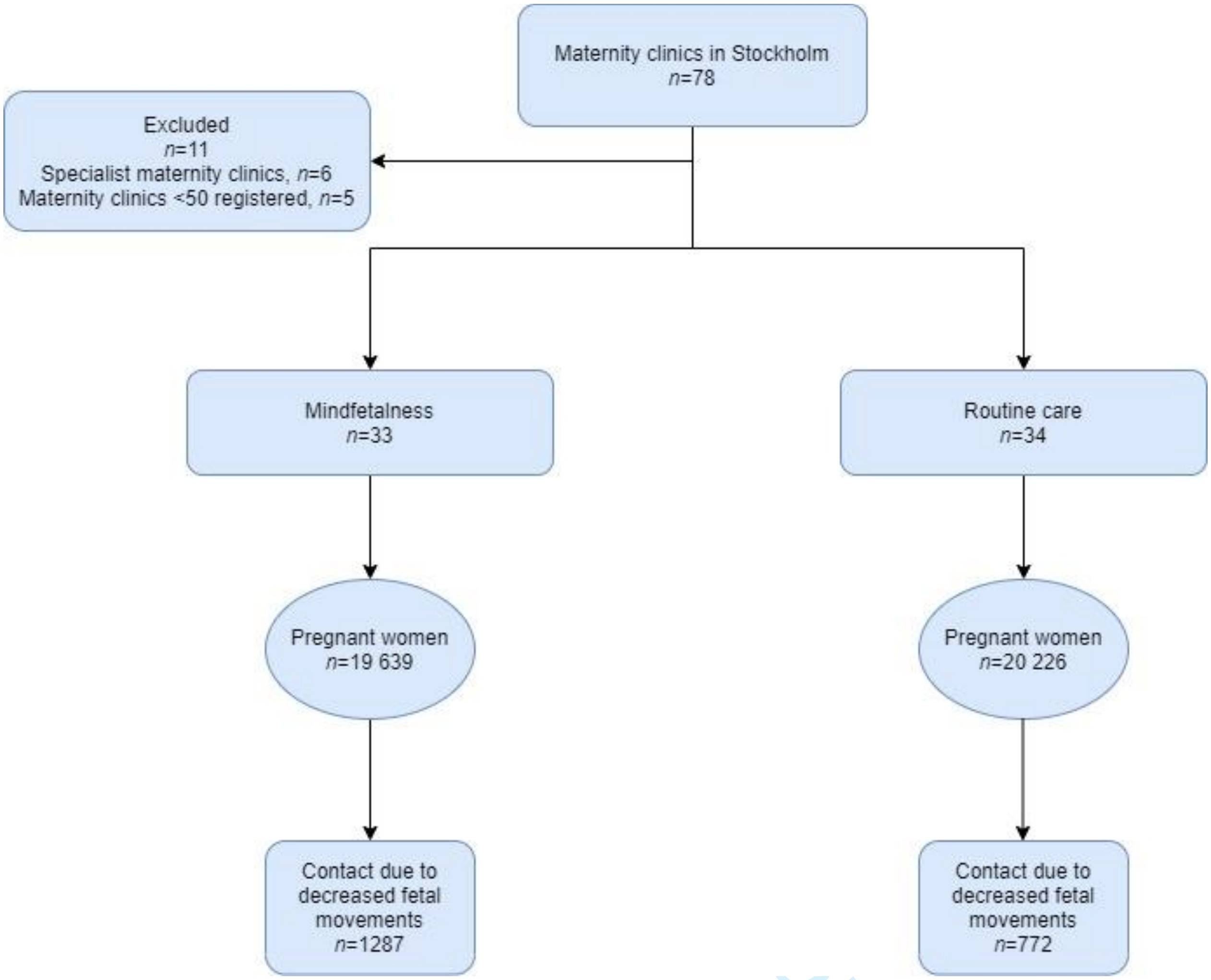

Figure 2a. Birth outcome among women who contacted healthcare due to decreased fetal movements from 32 weeks' gestation, where the examination did not indicate that the pregnancy needed to be terminated, at the time the women sought care.

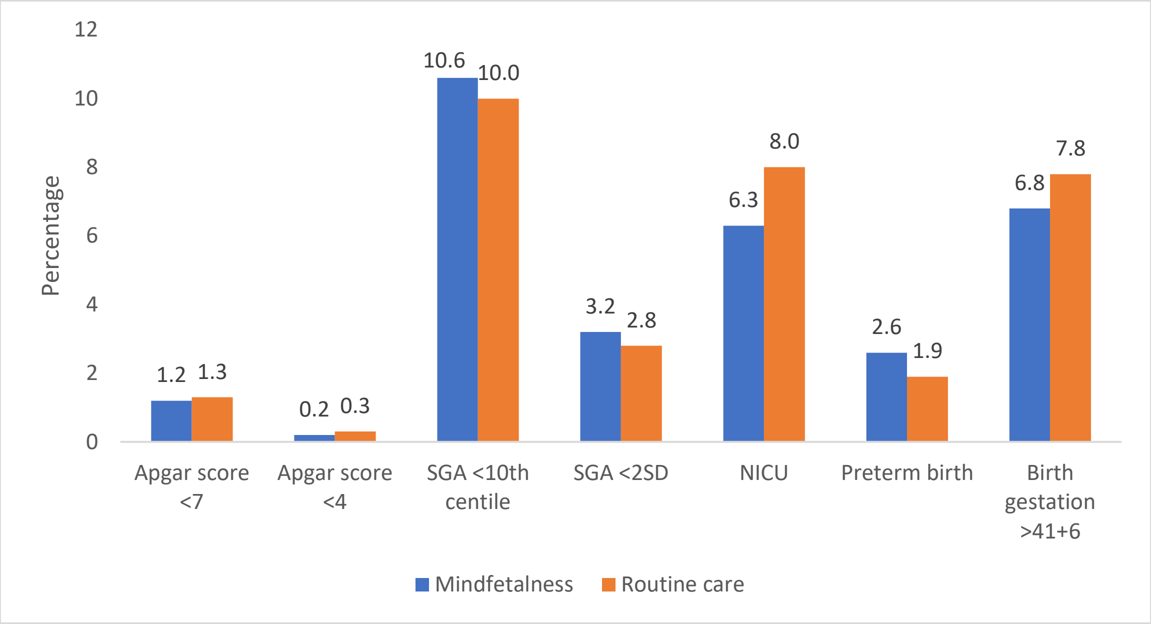

Figure 2b. Start of labor among women who contacted healthcare due to decreased fetal movements from 32 weeks' gestation, where the examination did not indicate that the pregnancy needed to be terminated, at the time the women sought care.

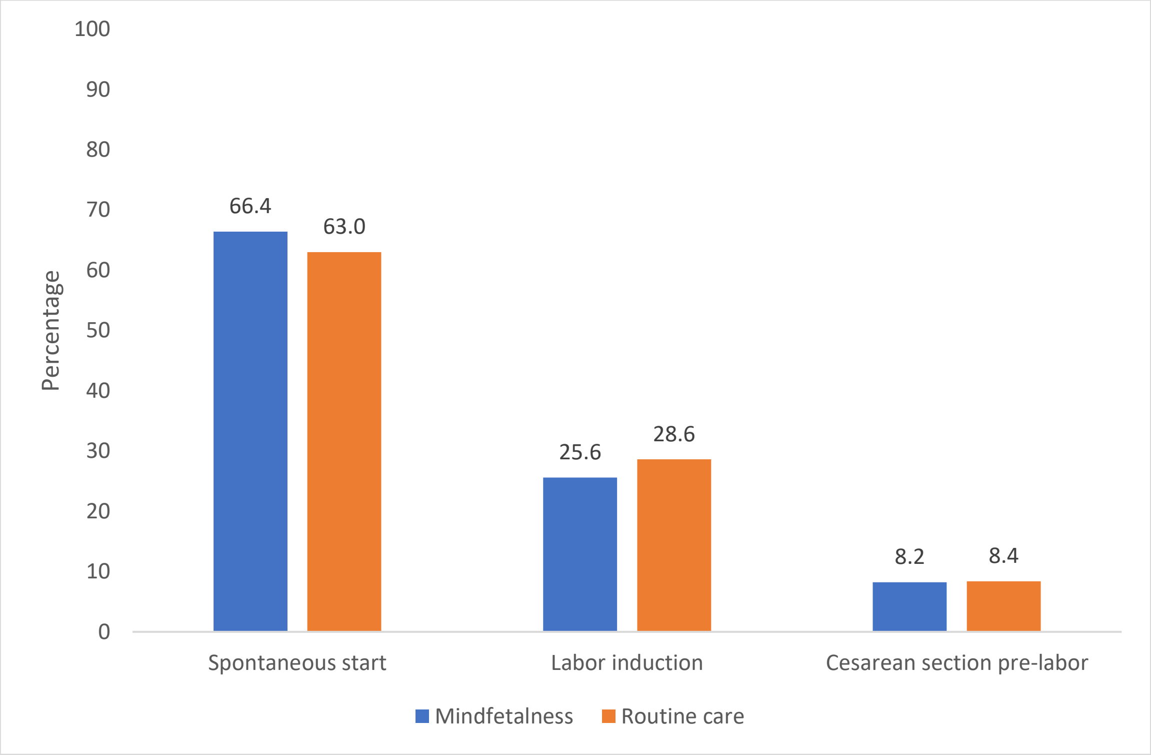

Supplementary Table 1. Percentage of women who sought care due to decreased fetal movements in Mindfetalness (*n*=19 639) versus routine care (*n*=20 226), divided into characteristics and a calculation of absolute differences between the groups.

**Characteristics**	**Mindfetalness** *n*=1287	**Percentage sought care**	**Routine care** *n*=772	**Percentage sought care**	**Absolute difference**
**Age**					
≤24 years	122/1483	8.2%	65/1322	4.9%	3.3%
25–34 years	859/12 715	6.8%	502/12 783	3.9%	2.9%
≥35 years	306/5441	5.6%	205/6111	3.4%	2.2%
**Country of birth**					
Sweden	1011/13 029	7.8%	584/13 456	4.3%	3.5%
Europe (except Sweden)	82/2026	4.0%	55/1891	2.9%	1.1%
Africa	28/1063	2.6%	23/1438	1.6%	1.0%
Asia	134/2989	4.5%	90/2898	3.1%	1.4%
North America	9/166	5.4%	7/154	4.5%	0.9%
South America	19/328	5.8%	12/325	3.7%	2.1%
Other	4/38	10.5%	1/64	1.6%	8.9%
**Education level¶**					
Shorter than 9 years	4/203	2.0%	7/359	1.9%	0.1%
Elementary school	48/916	5.2%	21/887	2.4%	2.8%
High school	339/5192	6.5%	207/5071	4.1%	2.4%
University	829/11 916	7.0%	461/12 088	3.8%	3.2%
**Parity §**					
Primipara	686/8544	8.0%	441/8927	4.9%	3.1%
Multipara	600/11 012	5.4%	330/11 242	2.9%	2.5%
**Assisted reproduction§**	79/1078	7.3%	50/1166	4.3%	3.0%
**Previous stillbirth**	9/116	7.8%	0/104	0%	7.8%
**Tobacco user at registration at the maternity clinic§**	56/737	7.6%	23/598	3.8%	3.8%
**Body Mass Index∫**					
<18.5 kg/m²	38/568	6.7%	16/507	3.2%	3.5%
18.5–24.9 kg/m²	771/11 828	6.5%	457/12 024	3.8%	2.7%
25.0–29.9 kg/m²	285/4336	6.6%	179/4739	3.8%	2.8%
30.0–34.9 kg/m²	107/1488	7.2%	60/1568	3.8%	3.4%
≥35.0 kg/m²	38/533	7.1%	25/530	4.7%	2.4%
**Maternal diseases***					
Psychiatric care	225/2468	9.1%	129/2630	4.9%	4.2%
Medication or psychologic treatment for mental illness	82/1160	7.1%	47/935	5.0%	2.1%

¶ Data are missing for 3,344 women (1,412 in Mindfetalness group and 1,932 in Routine care group)
§ Data are missing for 140 women (83 in Mindfetalness group and 57 in Routine care group)
∫ Data are missing for 1,744 women (886 in Mindfetalness group and 858 in Routine care group)
*Selected common diseases that can be associated with search behavior

For peer review only

Supplementary Table 2. Obstetric outcome from gestation week 32+0 among women with singleton pregnancy who have contacted healthcare due to decreased fetal movements; 1287 registered at a maternity clinic randomized to Mindfetalness, and 772 registered at a maternity clinic randomized to routine care.

**Outcome**	**Mindfetalness *n* (%)**	**Routine care *n* (%)**	**Rate Ratio (95% CI)**	***p*-value**
Apgar Score <10 at 5 minutes†	147 (11.4)	88 (11.4)	1.00 (0.79–1.29)	0.97
Apgar Score <7 at 5 minutes†	15 (1.2)	10 (1.3)	0.90 (0.41–2.06)	0.80
Apgar Score < 4 at 5 minutes†	3 (0.2)	2 (0.3)	0.90 (0.15–6.84)	0.91
Birthweight <10th centile*‡	136 (10.6)	77 (10.0)	1.06 (0.82–1.39)	0.67
Birthweight <2SD**‡	41 (3.2)	22 (2.8)	1.12 (0.68–1.89)	0.67
Admitted to NICU	81 (6.3)	62 (8.0)	0.78 (0.57–1.08)	0.14
Death within 27 days after birth	0 (0.0)	0 (0.0)	NA	NA
Preterm delivery (<37+0)	34 (2.6)	15 (1.9)	1.36 (0.76–2.55)	0.31
Birth gestation >41+6	87 (6.8)	60 (7.8)	0.87 (0.64–1.20)	0.39
Spontaneous start of labor	854 (66.4)	486 (63.0)	1.05 (0.99–1.13)	0.12
Induction of labor	329 (25.6)	221 (28.6)	0.89 (0.77–1.03)	0.13
Cesarean section (total)	251 (19.5)	154 (19.9)	0.98 (0.82–1.17)	0.81
Pre–labor	106 (8.2)	65 (8.4)	0.98 (0.73–1.32)	0.88
In labor	145 (11.3)	89 (11.5)	0.98 (0.76–1.26)	0.86

Stillborn routine care *n*=1 (0.1%), Mindfetalness *n*=0 (0.0)
†Data are missing for 3 women (3 in Mindfetalness group and 0 in Routine care group)
‡Data are missing for 2 women (1 in Mindfetalness group and 1 in Routine care group)
*for the gestational age (International definition of Small for Gestational Age)
**from the national reference mean (Swedish definition of Small for Gestational Age)
NICU: Neonatal intensive care unit

For peer review only

Mindfetalness – a method for focusing upon fetal movements

The first perception of fetal movements is sometimes described as a gentle tickling. As the pregnancy proceeds and the fetus develops the movements become more distinct. Just as new-born babies vary, there are differences between fetuses. Some fetuses are very active during their time in the uterus whilst others are calm, but all fetuses move up until birth. Each unborn baby has its own pattern of movements and at the end of the pregnancy the pattern can be recognised. The fetus moves between periods of wakefulness with much movement and calmer periods of rest. Movement frequency usually peaks around week 32 of pregnancy and remains for the most part at that level until delivery. The movements increase in strength as the fetus grows but at the end of the pregnancy can be experienced differently to the movements felt earlier.

That the fetus moves is a good sign and many pregnant women describe how they notice their unborn baby's movements every day. One systematic way of observing the movements is the method of *Mindfetalness* that can be used to get to know the movement pattern. An appropriate time to begin with *Mindfetalness* is in gestational week 28.

How to put Mindfetalness into practice

Mindfetalness is practiced ideally on a daily basis. **Choose a time of day that suits you best but also wait until you feel that your unborn baby is having a period of wakefulness.** Lie down or sit comfortably when you engage in Mindfetalness. If you lie down, then preferably on your left side. The movements are felt more distinctly when you lie down, and the blood flow is at its best in the uterus on the left side, which is good for the fetus. Concentrate on your unborn baby's movements for approximately 15 minutes. You will feel yourself if you need a longer or shorter time to perceive the movements. For some women it is enough just to get an idea of how the movements feel, others prefer to write notes about what they experience. At the end of this information there is space in which you can write down how you experience the movements. If you have a smartphone or computer you can write your impressions at www.mindfetalness.com

During Mindfetalness you focus upon:
The intensity of the movements
The way in which the baby moves
How much the baby moves

The questions to be answered are:
Can the movements be felt distinctly?
Are the movements of the same intensity as usual?
Does the fetus move as much as usual?

For peer review only - http://bmjopen.bmj.com/site/about/guidelines.xhtml

Fetal movements at the end of the pregnancy

The unborn baby's movements can be divided into two main groups: large movements and small movements. The large movements are felt distinctly; this can be when the fetus kicks or stretches out its body. The small movements that the fetus makes, but which are not felt, are gripping movements with fingers and toes as well as breathing movements. In approximately weeks 25 to 30 the movements begin to become organized and the unborn baby has periods of wakefulness interspersed with periods of rest lasting approximately 40 minutes up to an hour.

The movements change as the pregnancy proceeds and can feel different due to the fact that the space the fetus has at its disposal becomes smaller, although this does not affect the frequency of the movements. Women at full term pregnancy often describe how the movements feel powerful, pushing, stretching, large, from side to side, slow and light.

If you focus upon the fetal movements for a while every day when the fetus has a period of wakefulness, you can gain a good understanding of your unborn baby's movement pattern. It is important the observation occurs when the fetus is awake (the fetus is less active during a rest period). There can be wide variations from fetus to fetus regarding the frequency and intensity of the movements.

Women who have tried *Mindfetalness* describe how they felt calm, present and focussed while using the method. They also describe the period as a communication with their unborn baby and that they experienced a powerful bonding with their baby. Only you can decide whether the method suits you.

For peer review

Summary

The movements become organised during pregnancy weeks 25 to 30 and the fetus has periods of wakefulness interspersed with periods of rest, approximately 40 minutes up to an hour. Most fetuses have, at the end of the pregnancy, a daily rhythm and are active in the evening.

In pregnancy week 32 a plateau phase is reported regarding the frequency of movements but there is nothing to indicate that the movements decrease at the end of pregnancy.

There can be a wide variation between fetuses in the frequency and intensity of their movements.

Get to know your unborn baby's movement pattern during pregnancy. Trust your intuition.

If you are concerned that the fetus is moving less or that the movements are weaker, you should contact health care.

What does the fetus do in the uterus?
As the fetus grows, the movements become more distinct and successively regular. Small movements are not felt, e.g. when the fetus sucks its thumb or flexes its toes. Kicks, and when the child stretches out, can usually be felt clearly and many also feel when the child hiccups (small rhythmic jerks) during the latter part of pregnancy.

During the final months of the pregnancy the movements are distinct and powerful, but may be experienced as being of a different character compared with when the fetus had a larger space at its disposal. Some women describe how the unborn baby stretches out, as if the fetus is trying to stretch as the space begins to be tight. Others describe the movements as large, that they involve the unborn baby's whole body and can be described as slow. The larger the fetus the more distinct the movement.

Is it true that the fetus moves less towards the end of pregnancy?
No, this is not true. Fetal movements increase up until pregnancy week 32, thereafter and up until delivery, the frequency of movements generally remains the same. It is important to remember that the fetus should continue to be active throughout the pregnancy.

Does the fetus move the whole time?
The fetus does not move the whole time. All unborn babies are calm and sleep for short periods. There can be wide variations from fetus to fetus regarding the frequency and intensity of the movements.

Can it be more difficult for some women to feel the movements?
It is probable that it is easier to feel the movements if the woman lies on her left side and concentrates on them. Some women describe how, in spite of doing so, they have great difficulty in feeling their unborn baby move. Extreme overweight, amongst other things, can make it more difficult. If one is much stressed it may be more difficult to feel the movements.

What should I do if I feel that the movements become fewer towards the end of the pregnancy?
If the movements decrease in intensity or frequency, and deviate from the fetus' normal way of moving, it can be a sign that the child is not doing so well in the uterus. Most pregnant women who experience fewer and weaker movements give birth to a healthy child, but there is an increased risk that the fetus is not fit. If you experience that the movements have become fewer and weaker, and you feel that there is a difference compared with earlier in the pregnancy it should *not* be interpreted as something normal until the child has been examined.

For peer review only - http://bmjopen.bmj.com/site/about/guidelines.xhtml

Diary of my unborn baby's activities

Pregnancy week	How my unborn baby moved
28 +	
**Pregnancy week** 29 +	**How my unborn baby moved**
**Pregnancy week** 30 +	**How my unborn baby moved**
**Pregnancy week** 31 +	**How my unborn baby moved**

For peer review only

For peer review only - http://bmjopen.bmj.com/site/about/guidelines.xhtml

Diary of my unborn baby's activities

Pregnancy week	How my unborn baby moved
32 +	
Pregnancy week 33 +	How my unborn baby moved
Pregnancy week 34 +	How my unborn baby moved
Pregnancy week 35 +	How my unborn baby moved

For peer review only

For peer review only - http://bmjopen.bmj.com/site/about/guidelines.xhtml

Diary of my unborn baby's activities

Week of pregnancy	How my unborn baby moved
Week of pregnancy 36 +	
Week of pregnancy 37 +	How my unborn baby moved
Week of pregnancy 38 +	How my unborn baby moved
Week of pregnancy 39 +	How my unborn baby moved

For peer review only

For peer review only - http://bmjopen.bmj.com/site/about/guidelines.xhtml

Diary of my unborn baby's activities

Week of pregnancy 40 +	How my unborn baby moved
**Week of pregnancy 41 +**	**How my unborn baby moved**
**Week of pregnancy 42 +**	**How my unborn baby moved**

For peer review only

For peer review only - http://bmjopen.bmj.com/site/about/guidelines.xhtml

STROBE 2007 (v4) checklist of items to be included in reports of observational studies in epidemiology*

Checklist for cohort, case-control, and cross-sectional studies (combined)

Section/Topic	Item #	Recommendation	Reported on page #
**Title and abstract**	1	(*a*) Indicate the study's design with a commonly used term in the title or the abstract	1-2
		(*b*) Provide in the abstract an informative and balanced summary of what was done and what was found	2
**Introduction**			
Background/rationale	2	Explain the scientific background and rationale for the investigation being reported	4
Objectives	3	State specific objectives, including any pre-specified hypotheses	4
**Methods**			
Study design	4	Present key elements of study design early in the paper	5
Setting	5	Describe the setting, locations, and relevant dates, including periods of recruitment, exposure, follow-up, and data collection	5 and in previous papers
Participants	6	(*a*) *Cohort study*—Give the eligibility criteria, and the sources and methods of selection of participants. Describe methods of follow-up<br>*Case-control study*—Give the eligibility criteria, and the sources and methods of case ascertainment and control selection. Give the rationale for the choice of cases and controls<br>*Cross-sectional study*—Give the eligibility criteria, and the sources and methods of selection of participants	5
		(*b*) *Cohort study*—For matched studies, give matching criteria and number of exposed and unexposed<br>*Case-control study*—For matched studies, give matching criteria and the number of controls per case	
Variables	7	Clearly define all outcomes, exposures, predictors, potential confounders, and effect modifiers. Give diagnostic criteria, if applicable	5-6
Data sources/ measurement	8*	For each variable of interest, give sources of data and details of methods of assessment (measurement). Describe comparability of assessment methods if there is more than one group	*5-6*
Bias	9	Describe any efforts to address potential sources of bias	5-6 and in previous papers
Study size	10	Explain how the study size was arrived at	5 and in previous papers
Quantitative variables	11	Explain how quantitative variables were handled in the analyses. If applicable, describe which groupings were chosen and why	6
Statistical methods	12	(*a*) Describe all statistical methods, including those used to control for confounding	6
		(*b*) Describe any methods used to examine subgroups and interactions	6

For peer review only

		(*c*) Explain how missing data were addressed	Negligible due to unique population-based register
		(*d*) *Cohort study*—If applicable, explain how loss to follow-up was addressed<br>*Case-control study*—If applicable, explain how matching of cases and controls was addressed<br>*Cross-sectional study*—If applicable, describe analytical methods taking account of sampling strategy	6
		(*e*) Describe any sensitivity analyses	
**Results**			
Participants	13*	(a) Report numbers of individuals at each stage of study—eg numbers potentially eligible, examined for eligibility, confirmed eligible, included in the study, completing follow-up, and analysed	6-9
		(b) Give reasons for non-participation at each stage	NA
		(c) Consider use of a flow diagram	Yes, included
Descriptive data	14*	(a) Give characteristics of study participants (eg demographic, clinical, social) and information on exposures and potential confounders	6-7 and Suppl. Table 1
		(b) Indicate number of participants with missing data for each variable of interest	6-9
		(c) *Cohort study*—Summarise follow-up time (eg, average and total amount)	
Outcome data	15*	*Cohort study*—Report numbers of outcome events or summary measures over time	6-9
		Case-control study—Report numbers in each exposure category, or summary measures of exposure	
		Cross-sectional study—Report numbers of outcome events or summary measures	
Main results	16	(*a*) Give unadjusted estimates and, if applicable, confounder-adjusted estimates and their precision (eg, 95% confidence interval). Make clear which confounders were adjusted for and why they were included	8
		(*b*) Report category boundaries when continuous variables were categorized	Yes, 6-9
		(*c*) If relevant, consider translating estimates of relative risk into absolute risk for a meaningful time period	Yes, 6-9
Other analyses	17	Report other analyses done—eg analyses of subgroups and interactions, and sensitivity analyses	Suppl. Table 1
**Discussion**			
Key results	18	Summarise key results with reference to study objectives	9-10
Limitations	19	Discuss limitations of the study, taking into account sources of potential bias or imprecision. Discuss both direction and magnitude of any potential bias	11-13
Interpretation	20	Give a cautious overall interpretation of results considering objectives, limitations, multiplicity of analyses, results from similar studies, and other relevant evidence	13
Generalisability	21	Discuss the generalisability (external validity) of the study results	13
**Other information**			
Funding	22	Give the source of funding and the role of the funders for the present study and, if applicable, for the original study on which the present article is based	17

For peer review only

*Give information separately for cases and controls in case-control studies and, if applicable, for exposed and unexposed groups in cohort and cross-sectional studies.

Note: An Explanation and Elaboration article discusses each checklist item and gives methodological background and published examples of transparent reporting. The STROBE checklist is best used in conjunction with this article (freely available on the Web sites of PLoS Medicine at http://www.plosmedicine.org/, Annals of Internal Medicine at http://www.annals.org/, and Epidemiology at http://www.epidem.com/). Information on the STROBE Initiative is available at www.strobe-statement.org.

For peer review only

Country of birth, educational level and other predictors of seeking care due to decreased fetal movements: An observational study in Sweden using data from a cluster-randomized controlled trial

Journal:	*BMJ Open*
Manuscript ID	bmjopen-2021-050621.R2
Article Type:	Original research
Date Submitted by the Author:	31-May-2021

Complete List of Authors:	Radestad, Ingela; Sophiahemmet University<br>Pettersson, Karin; Karolinska Institute, Department of Clinical Science, Intervention and Technology<br>Lindgren, Helena; Karolinska Institute, Women's and Children's Health<br>Skokic, Viktor; University of Gothenburg Sahlgrenska Academy, Oncology<br>Akselsson, Anna; Sophiahemmet University, Health Promoting Science

**Primary Subject Heading**:	Obstetrics and gynaecology
Secondary Subject Heading:	Epidemiology
Keywords:	Fetal medicine < OBSTETRICS, Maternal medicine < OBSTETRICS, Prenatal diagnosis < OBSTETRICS

SCHOLARONE™ Manuscripts

I, the Submitting Author has the right to grant and does grant on behalf of all authors of the Work (as defined in the below author licence), an exclusive licence and/or a non-exclusive licence for contributions from authors who are: i) UK Crown employees; ii) where BMJ has agreed a CC-BY licence shall apply, and/or iii) in accordance with the terms applicable for US Federal Government officers or employees acting as part of their official duties; on a worldwide, perpetual, irrevocable, royalty-free basis to BMJ Publishing Group Ltd ("BMJ") its licensees and where the relevant Journal is co-owned by BMJ to the co-owners of the Journal, to publish the Work in this journal and any other BMJ products and to exploit all rights, as set out in our licence.

The Submitting Author accepts and understands that any supply made under these terms is made by BMJ to the Submitting Author unless you are acting as an employee on behalf of your employer or a postgraduate student of an affiliated institution which is paying any applicable article publishing charge ("APC") for Open Access articles. Where the Submitting Author wishes to make the Work available on an Open Access basis (and intends to pay the relevant APC), the terms of reuse of such Open Access shall be governed by a Creative Commons licence – details of these licences and which Creative Commons licence will apply to this Work are set out in our licence referred to above.

Other than as permitted in any relevant BMJ Author's Self Archiving Policies, I confirm this Work has not been accepted for publication elsewhere, is not being considered for publication elsewhere and does not duplicate material already published. I confirm all authors consent to publication of this Work and authorise the granting of this licence.

For peer review only

Country of birth, educational level and other predictors of seeking care due to decreased fetal movements: An observational study in Sweden using data from a cluster-randomized controlled trial

Ingela Rådestad, RNM, Professor, Sophiahemmet University, Stockholm, Sweden.
E-mail: ingela.radestad@shh.se

Karin Pettersson, MD, Associate Professor, Department of Clinical Science, Intervention and Technology, Karolinska Institutet, Stockholm, Sweden. E-mail: karin.ma.pettersson@sll.se

Helena Lindgren, Associate Professor, Department of Women's and children's health, Karolinska Institutet, Stockholm, Sweden. E-mail: Helena.lindgren@ki.se

Viktor Skokic, Statistician, Institute of Clinical Sciences, Sahlgrenska Academy, University of Gothenburg, Gothenburg, Sweden. E-mail: viktor.skokic@gu.se

Anna Akselsson*, RNM, PhD, Department of Health Promoting Science, Sophiahemmet University, Karolinska Institutet, Department of Women's and children's health, Stockholm, Sweden. E-mail: anna.akselsson@shh.se

*Corresponding author
Anna Akselsson, RNM, PhD
Sophiahemmet University
PB 5605, S-114 86, Stockholm, Sweden.
E-mail: anna.akselsson@shh.se
Phone: +46 8 406 20 79

For peer review only - http://bmjopen.bmj.com/site/about/guidelines.xhtml

Abstract

Objectives Predictors of seeking care for decreased fetal movements and whether care-seeking behavior was influenced by Mindfetalness.

Design Observational study with data from a cluster-randomized controlled trial.

Setting 67 maternity clinics and six obstetrical clinics in Sweden.

Participants All pregnant women with a singleton pregnancy who contacted the obstetrical clinic due to decreased fetal movements from 32 weeks' gestation of 39 865 women.

Methods Data were collected from a cluster-randomized controlled trial where maternity clinics were randomized to Mindfetalness or routine care. Mindfetalness is a self-assessment method for women to use daily to become familiar with the unborn baby's fetal movement pattern.

Outcome measures Predictors for contacting healthcare due to decreased fetal movements.

Results Overall, 5.2% (n=2059) of women contacted healthcare due to decreased fetal movements, among which 1287 women (62.5%) were registered at a maternity clinic randomized to Mindfetalness and 772 women (37.5%) were randomized to routine care. Predictors for contacting healthcare due to decreased fetal movements were age, country of birth, educational level, parity, prolonged pregnancy and previous psychiatric care (p<0.001). The main differences were seen among women born in Africa as compared to Swedish-born women (2.0% vs. 6.0%, RR 0.34, CI 0.25-0.44) and among women with low educational level compared to women with university-level education (2.0% vs. 5.4%, RR 0.36, CI 0.19-0.62). Introducing Mindfetalness in maternity care increased the number of women seeking care due to decreased fetal movements overall.

Conclusion Women with country of birth outside Sweden and low educational level sought care for decreased fetal movements to a lesser extent compared to women born in Sweden and those with university degrees. Future research could explore whether pregnancy outcomes can be improved by motivating women in these groups to contact healthcare if they feel a decreased strength or frequency of fetal movements.

Trial registration www.clinicaltrials.gov (NCT02865759).

For peer review only - http://bmjopen.bmj.com/site/about/guidelines.xhtml

Strengths and limitations of this study

- This is the first study exploring the association between country of birth and contact due to decreased fetal movements.

- The new information about predictors for contacting healthcare due to decreased fetal movements can be valuable for preventing stillbirth.

- Zero lost to follow up, large sample size and population-based register is a methodological strength.

- Misclassified subjects might have affected result of the predictors and it is probably an underestimation of the number of women seeking care.

- Possible confounders, not being considered in the study, could have had an impact of the result.

Introduction

A woman's interpretation of signals from the fetus and her ability to take action will determine whether she contacts healthcare when she has concerns for her unborn baby's health. Her response to altered fetal movement patterns can be decisive for the pregnancy outcomes. Among women who have sought care due to decreased fetal movements, reports indicate that one quarter will later experience adverse baby outcomes, such as low Apgar score, small for gestational age, or stillbirth [1-3]. Further, up to fifty percent of women who experience stillbirth report a gradual decrease in fetal movements several days before the baby died [4,5]. The majority waited more than 24 hours before contacting healthcare without the perception of any movements [6,7]. Contact with healthcare due to decreased fetal movements is common and most of the women return home after normal findings are confirmed. The prevalence of women contacting healthcare with concerns of fetal movements varies internationally; from six to 34 percent [8-11].

Women with normal pregnancies can distinguish between many different types of fetal movements and most feel strong movements [12]. Maternal perception of fetal movements and becoming familiar with the movement pattern is important in reducing stillbirth, as reported in research from New Zealand [13]. The character and diurnal rhythm of movements seem to be essential factors in preventing stillbirth. Maternally perceived increased strength of fetal

For peer review only - http://bmjopen.bmj.com/site/about/guidelines.xhtml

movements is associated with reduced stillbirth rate (aOR 0.21, 95% CI 0.12–0.36) and quiet or light movements in the evening were associated with higher risk (aOR 3.82, 95% CI 1.57–9.31).

Raising maternal awareness of fetal movements by using the Mindfetalness method may be a way forward for women to become familiar with the unborn baby's movement pattern [14]. Compared to counting methods [15,16], Mindfetalness instruct the women to observe the variation of fetal movements. Practicing the method includes to lie down for 15 minutes a day from 28 weeks' gestation, when the baby is awake and to focus on the character, strength and frequency of the unborn baby's movements, without counting each movement. Mindfetalness was invented in 2012 [14] and has been evaluated by women and midwives in two studies [17,18]. Additionally, in a cluster randomized controlled trial, 67 maternity clinics were allocated to either Mindfetalness or routine care [19,20]. Midwives in the Mindfetalness group were instructed to distribute oral and written information about fetal movements and about Mindfetalness. Information regarding the method was available in nine different languages and a website was open for anyone to access. The trial showed several benefits of introducing Mindfetalness into maternity care [19]. In the Mindfetalness group, more women started their labor spontaneously, followed by a reduction in labor inductions and cesarean sections. Further, less babies were born small for gestational age and needed neonatal care.

The ambition to encourage women to seek care if they have concerns of their unborn baby's fetal movements pattern is to reduce the stillbirth rate without too much overload on both women and caregivers. However, stillbirth is a very rare phenomenon and thus comparing stillbirth rate in this study is impossible. In this study, we investigate predictors of seeking care for decreased fetal movements and whether care-seeking behavior was influenced by a method (Mindfetalness) aimed at increasing pregnant women's knowledge of their unborn baby's fetal movement patterns [14]. We further investigate pregnancy outcomes for women seeking care for decreased fetal movements but where, at the time of the examination, no interventions such as induction of labor or caesarean section were made.

For peer review only - http://bmjopen.bmj.com/site/about/guidelines.xhtml

Methods

Pregnant women with a singleton pregnancy who contacted healthcare due to decreased fetal movements from 32 weeks' gestation constitute the population of this study. Data were collected from a cluster-randomized controlled trial in which 67 maternity clinics in Stockholm, Sweden, were randomized to either intervention with Mindfetalness or routine care, as reported previously [19,20]. The women who contacted healthcare due to decreased fetal movements from 32 weeks' gestation were generated from the ICD-code AM041 (examination due to decreased fetal movements) [21]. The code is a diagnosis for pregnant women who have contacted healthcare due to decreased fetal movements, where no interventions, such as labor induction or cesarean section, were made, at the time the woman sought care.

The randomization resulted in 33 maternity clinics in the Mindfetalness group and 34 in routine care group. After the randomization, one clinic randomized to Mindfetalness declined participation. We applied intention-to-treat design; extended information about the randomization process can be found in previous publications [19,20]. The Mindfetalness group included 19 639 women and the routine care group 20 226 women.

Starting in October 2016, midwives, working at maternity clinics randomized to the intervention, distributed a leaflet to pregnant women, including information about fetal movements and the self-assessment method, Mindfetalness. The last leaflet was distributed on 31 January 2018, and the women were followed-up until birth in population-based registers. Midwives in the routine care group followed standard care, which includes providing oral information about fetal movements at 24 weeks' gestation.

The Mindfetalness leaflet (appendix) included information about fetal movements and when to contact healthcare; "If you are concerned that the fetus is moving less or that the movements are weaker, you should contact healthcare." Further, the leaflet included an invitation to practice Mindfetalness daily from 28 weeks' gestation. The information was available in nine different languages and the website www.mindfetalness.com was open for anyone to access.

This study was approved by the Regional Ethics committee in Stockholm, Sweden (Dnr 2015/2105–31/1), 13 January 2016.

For peer review only

Statistical analysis

We identified the background factors of the women who contacted healthcare due to decreased fetal movements by calculating percentages. The included variables were age, country of birth, education level, parity, assisted reproduction, previous stillbirth, tobacco use at registration in maternity clinic, body mass index, and maternal diseases. To calculate predictors for contacting healthcare, we investigated which factors were statistically significantly associated with contact due to decreased fetal movements. As a metric for association, we calculated relative risks and 95% confidence intervals using log-binomial regression. In the predictor calculation, we adjusted for the possible confounding factor, parity. For outcome measures, we calculated percentage and compared the Mindfetalness group with the routine care group using log-binomial regression. We considered a *p*-value of 0.05 to be statistically significant. We used the statistical program R (version 3.2.4). Loss to follow-up was zero due to the population-based Swedish Pregnancy Register [22] used for data collection.

Results

Among all women in this study, 5.2% (*n*=2059) contacted healthcare due to decreased fetal movements, measured from gestational week 32 until birth. As shown in Figure 1, 62.5% (*n*=1287) were registered at a maternity clinic randomized to Mindfetalness, and 37.5% (*n*=772) to routine care. The background data for the women are displayed in Table 1.

Table 1. Characteristics among women with a singleton pregnancy from 32 weeks' gestation contacting healthcare versus not contacting healthcare due to decreased fetal movements.

Characteristics	Contacted healthcare due to decreased fetal movements (*n*=2059) *n* (%)	Did not contact healthcare due to decreased fetal movements (*n*=37 806) *n* (%)	Relative risk (CI)	*p*-value
**Age***				
≤24 years	187 (9.1)	2628 (7.0)	1.31 (1.13–1.51)	<0.001
25–34 years	1361 (66.1)	24 137 (63.8)	1.04 (1.00–1.07)	0.04
≥35 years	511 (24.8)	11 041 (29.2)	0.85 (0.79–0.92)	<0.001
**Country of birth**				
Sweden	1595 (77.5)	24 890 (65.8)	1.18 (1.15–1.21)	<0.001
Europe (except Sweden)	137 (6.7)	3780 (10.0)	0.67 (0.56–0.79)	<0.001
Africa	51 (2.5)	2450 (6.5)	0.38 (0.29–0.50)	<0.001
Asia	224 (10.9)	5663 (15.0)	0.73 (0.64–0.82)	<0.001
North America	16 (0.8)	304 (0.8)	0.97 (0.59–1.60)	1.00

For peer review only - http://bmjopen.bmj.com/site/about/guidelines.xhtml

South America	31 (1.5)	622 (1.6)	0.92 (0.64–1.31)	0.72
Others	5 (0.2)	97 (0.3)	0.95 (0.39–2.33)	1.00
**Education level¶**				
Shorter than 9 years	11 (0.5)	551 (1.5)	0.36 (0.20–0.66)	<0.001
Elementary school	69 (3.4)	1734 (4.6)	0.72 (0.57–0.91)	0.006
High school	546 (26.5)	9717 (25.7)	1.02 (0.95–1.10)	0.64
University	1290 (62.7)	22 714 (60.1)	1.03 (1.00–1.06)	0.09
**Parity §**				
Primipara	1127 (54.7)	16 344 (43.2)	1.26 (1.21–1.32)	<0.001
Multipara	930 (45.2)	21 324 (56.4)	0.80 (0.76–0.84)	<0.001
**Assisted reproduction§**	129 (6.3)	2033 (5.4)	1.17 (0.98–1.38)	0.09
**Previous stillbirth**	9 (0.4)	211 (0.6)	0.78 (0.40–1.52)	0.64
**Tobacco user at registration in the maternity clinic (including snuff) §**	79 (3.8)	1256 (3.3)	1.16 (0.92–1.44)	0.21
**Body Mass Index∫**				
<18.5 kg/m²	54 (2.6)	1021 (2.7)	0.97 (0.74–1.27)	0.89
18.5–24.9 kg/m²	1228 (59.6)	22 624 (59.8)	0.99 (0.96–1.03)	0.70
25.0–29.9 kg/m²	464 (22.5)	8611 (22.8)	0.99 (0.91–1.07)	0.74
30.0–34.9 kg/m²	167 (8.1)	2889 (7.6)	1.06 (0.91–1.23)	0.47
≥35.0 kg/m²	63 (3.1)	1000 (2.6)	1.15 (0.90–1.48)	0.26
**Maternal diseases**				
Diabetes mellitus	1 (0.0)	63 (0.2)	0.29 (0.04–2.10)	0.26
Coronary heart disease	25 (1.2)	600 (1.6)	0.77 (0.51–1.14)	0.20
Thrombosis	13 (0.6)	283 (0.7)	0.84 (0.49–1.47)	0.69
Systemic lupus erythematosus	1 (0.0)	58 (0.2)	0.32 (0.04–2.28)	0.37
Psychiatric care	354 (17.2)	4744 (12.5)	1.37 (1.24–1.51)	<0.001
Endocrine disease	156 (7.6)	2633 (7.0)	1.09 (0.93–1.27)	0.29
Epilepsy	10 (0.5)	167 (0.4)	1.10 (0.58–2.08)	0.73
Chronic hypertension	9 (0.4)	184 (0.5)	0.90 (0.46–1.75)	0.87
Other disease	204 (9.9)	3583 (9.5)	1.05 (0.91–1.20)	0.51
Medication or psychologic treatment for mental illness	129 (6.3)	2058 (5.4)	1.15 (0.97–1.37)	0.11

* Mean age of women who contacted care=31.2, who did not contact care=32.4 (*p*<0.001)
¶ Data missing for women who contacted care *n*=143 (6.9%), who did not contact care *n*=3090 (8.2%)
§ Data missing for women who contacted care *n*=2 (0.1%), who did not contact care *n*=138 (0.4%)
∫ Data missing for women who contacted care *n*=83 (4.0%), who did not contact care *n*=1661 (4.4%)

Predictors for seeking care for decreased fetal movements were age, country of birth, educational level, parity, prolonged pregnancy and previous psychiatric care (*p*<0.001) (Table 2). The main differences were seen in country of birth and educational levels. Women born in Africa sought care to a lesser extent as compared to women born in Sweden, 2.0% versus 6.0% (RR 0.34, CI 0.25–0.44).

Table 2. Predictors for contacting healthcare due to decreased fetal movements

Predictor	*p-value*	*n (%)*	*RR (CI)*	*p-value*	*AdjRR (CI)**
Age	<0.001			0.02	
≤24 years		187 (6.6)	1.24 (1.07–1.44)		1.15 (0.98–1.33)
25–34 years		1361 (5.3)	1.0 (Reference)		1.0 (Reference)
≥35 years		511 (4.4)	0.83 (0.75–0.91)		0.90 (0.82–1.00)

For peer review only - http://bmjopen.bmj.com/site/about/guidelines.xhtml

Country of birth	<0.001			<0.001	
Sweden		1595 (6.0)	1.0 (Reference)		1.0 (Reference)
Europe (except Sweden)		137 (3.5)	0.58 (0.49–0.69)		0.58 (0.49 – 0.69)
Africa		51 (2.0)	0.34 (0.25–0.44)		0.36 (0.27–0.47)
Asia		224 (3.8)	0.63 (0.55–0.72)		0.66 (0.57–0.75)
North America		16 (5.0)	0.83 (0.49–1.29)		0.83 (0.49–1.28)
South America		31 (4.7)	0.79 (0.54–1.09)		0.82 (0.56–1.13)
Others		5 (4.9)	0.81 (0.30–1.70)		0.84 (0.31–1.75)
Marital status	0.20			0.06	
Cohabiting with becoming father		1914 (5.2)	1.0 (Reference)		1.0 (Reference)
Single		29 (4)	0.76 (0.52–1.07)		0.73 (0.49–1.02)
Other family situation		114 (4.8)	0.92 (0.76–1.10)		0.87 (0.72–1.04)
Education level	<0.001			<0.001	
Shorter than 9 years		11 (2.0)	0.36 (0.19–0.62)		0.41 (0.21–0.69)
Elementary school		69 (3.8)	0.71 (0.56–0.90)		0.75 (0.58–0.94)
High school		546 (5.3)	0.99 (0.90–1.09)		1.00 (0.91–1.10)
University		1290 (5.4)	1.0 (Reference)		1.0 (Reference)
Parity	<0.001			NA	
Primipara		1127 (6.5)	1.54 (1.42–1.68)		NA
Multipara		930 (4.2)	1.0 (Reference)		NA
Gestation week >41+6	<0.001			0.01	
No			1.0 (Reference)		1.0 (Reference)
Yes			1.34 (1.13–1.56)		1.25 (1.06–1.46)
Assisted reproduction	0.10			0.55	
No		1928 (5.1)	1.0 (Reference)		1.0 (Reference)
Yes		129 (6.0)	1.16 (0.97–1.38)		1.05 (0.88–1.25)
Previous stillbirth	0.46			0.95	
No		2050 (5.2)	1.0 (Reference)		1.0 (Reference)
Yes		9 (4.1)	0.79 (0.38–1.41)		0.98 (0.47–1.74)
Tobacco user at registration at the maternity clinic§	0.22			0.17	
No			1.0 (Reference)		1.0 (Reference)
Yes		79 (5.9)	1.15 (0.92–1.42)		1.17 (0.93–1.44)
Body Mass Index	0.77			0.37	
<18.5 kg/m²		54 (5.0)	0.98 (0.74–1.26)		0.95 (0.72–1.23)
18.5–24.9 kg/m²		1228 (5.1)	1.0 (Reference)		1.0 (Reference)
25.0–29.9 kg/m²		464 (5.1)	0.99 (0.89–1.10)		1.03 (0.92–1.14)
30.0–34.9 kg/m²		167 (5.5)	1.06 (0.90–1.24)		1.12 (0.95–1.31)
≥35.0 kg/m²		63 (5.9)	1.15 (0.89–1.46)		1.22 (0.94–1.54)
Psychiatric care	<0.001			<0.001	
No		1705 (4.9)	1.0 (Reference)		1.0 (Reference)
Yes		354 (6.9)	1.42 (1.27–1.58)		1.37 (1.22–1.52)
Medication or psychologic treatment for mental illness	0.12			0.13	
No		1930 (5.1)	1.0 (Reference)		1.0 (Reference)
Yes		129 (5.9)	1.15 (0.96–1.36)		1.15 (0.96–1.36)

*Adjusted for parity

Further, women with lowest educational levels sought care due to decreased fetal movements to a lesser extent compared to women with university-level education (2.0% versus 5.4%, RR 0.36,

For peer review only - http://bmjopen.bmj.com/site/about/guidelines.xhtml

For peer review only

CI 0.19–0.62). Additionally, primiparas, women with prolonged pregnancies (>41+6 gestation weeks), and women with experience of psychiatric care more often sought care due to decreased fetal movements (Table 2). The predictors for contacting healthcare due to decreased fetal movements remain after adjustment for parity. Mindfetalness increased the prevalence of women contacting healthcare due to decreased fetal movements overall (6.6% versus 3.8%, RR 1.72, CI 1.57–1.87) (not in table). When stratifying women into groups according to background factors, the percentage of women contacting healthcare increased in all groups (Supplementray Table 1).

Figures 2a and 2b show the pregnancy outcomes for women contacting healthcare due to decreased fetal movements, stratified for women registered at maternity clinics randomized to Mindfetalness and routine care, respectively. Compared to routine care, women in the Mindfetalness group had a higher percentage of women with spontaneous start of labor (66.4% versus 63.0%) and a lower rate of labor induction (25.6% versus 28.6%). The proportion of women who had onset of labor from 42 weeks' gestation was 6.8% in the Mindfetalness group, and 7.8% in routine care. Further, a lower proportion of babies in the Mindfetalness group were in need of transfer to NICU (6.3% versus 8.0%). One stillbirth was observed, in the routine care group (not in table). There were no statistically significant differences in the investigated pregnancy outcomes in Figures 2a and 2b, between the Mindfetalness and routine care groups. For further information in pregnancy outcomes for Mindfetalness and routine care, see Supplementary Table 2.

Discussion

During the studied period, in the Stockholm region, women born outside Sweden and women with low levels of education sought care for decreased fetal movements to a lower extent when compared to women born in Sweden and women with university-level education. Further, women of higher age contacted healthcare due to decreased fetal movements to a lower extent than women of a younger age. Country of birth outside Sweden, low educational level and advanced maternal age have previously been related to adverse birth outcomes. Introducing Mindfetalness in maternity care increased the number of women contacting healthcare due to decreased fetal movements.

For peer review only - http://bmjopen.bmj.com/site/about/guidelines.xhtml

We found that women born outside Sweden (mostly from Africa and Asia) sought care due to decreased fetal movements to a lower extent. No studies, to our knowledge, have investigated the association between contact with healthcare due to maternal concern of fetal movements and country of birth. However, previous research has reported a higher risk of stillbirth among women not being born, but giving birth, in a Western country [23-28]. Suggested mechanisms for this difference include sub-standard maternity care, lack of communication between the pregnant woman and healthcare professionals, and variations in pre-pregnancy health [24,29]. It is possible that the higher risk for stillbirth can be linked to lower awareness and knowledge about fetal movements and when to contact healthcare [6,7].

Giving information to pregnant women about fetal movements may reduce pre-hospital delays [30-32] and has been suggested to reduce the stillbirth rate in an intervention study performed in Norway[32]. The study documented a 50 percent reduction in the stillbirth rate among women who contacted healthcare due to decreased fetal movements as compared to women who did not. The intervention included providing health promotion information to the pregnant women, an invitation to count fetal movements, and information that they should never wait until the next day to contact healthcare if they had concerns about fetal movements. Additionally, providing new guidelines to the healthcare professionals was included in the intervention. A reduction in patient delay and increased knowledge about fetal movements was shown in a recent study from the Netherlands after the introduction of a leaflet about fetal movements [33]. However, in the AFFIRM-trial[34], women in the intervention arm (including information to pregnant women and new guidelines to the healthcare professionals) had lower rate of stillbirths than women in the control group, but the difference was not statistically significant. Labor inductions and cesarean section increased, and the number of babies in need of neonatal care more than 48 hours.

The midwife is the most important source of information [35,36] and The Swedish National Board of Health and Welfare recently introduced new guidelines for maternity care, including instructions to midwives to inform pregnant women about fetal movements [37]. The guidelines have good compliance among midwives (79-87% answered yes to the different questions whether they followed the guidelines), but some report barriers due to lack of time, high workload and problems in communicating with women born outside of Sweden [38]. Suboptimal care is more

For peer review only - http://bmjopen.bmj.com/site/about/guidelines.xhtml

common among non-western women and action to improve perinatal mortality for this group is needed [24].

In this study, women with low education levels sought care due to decreased fetal movements to a lower extent, as compared to women with high education levels. A higher risk for stillbirth has been found among women with low educational levels and low-socioeconomic status [6,39-41]. There are reasons to believe that raising awareness about fetal movements among women with low education or socioeconomic status would increase the number of women seeking care due to decreased fetal movements, which in turn would possibly decrease perinatal mortality.

Women of advanced maternal age contacted healthcare due to decreased fetal movements to a lower extent than younger women in our study, and a higher risk of stillbirth is reported among women aged from 35 years compared to younger women [42]. The mean age in an Israeli study was lower among women who had contacted healthcare due to decreased fetal movements compared to the reference group [43]. In a study from Norway, the percentage of women over 35 years of age was lower among women who contacted healthcare due to decreased fetal movements compared to the reference group (16% versus 20%) [3]. However, in a recent systematic review, the authors interpreted current evidence as not supporting the notion that a woman's mean age influences whether she seeks care for decreased fetal movements [44].

Introducing Mindfetalness in maternity care increased the number of women contacting healthcare due to decreased fetal movements overall. Mindfetalness possibly increases the woman's ability to become familiar with the unborn baby's fetal movement patterns. Reducing pre-hospital delay when the perception that fetal movements are reduced is suggested to be one way for improving perinatal mortality [7,30,31].

Methodological considerations

The prevalence of women seeking care due to decreased or altered fetal movements in our study is low compared to the figures from other research. In a study performed in Sweden in 2014, 9.3 percent of the women sought care due to decreased fetal movements when measured from gestational week 28 until birth [9]. Further, international studies show a prevalence of 6–34 percent [8,10,11,45]. We believe that the figures in our study are underestimated, mostly due to the fact that we only included women who sought care from gestational week 32. Additionally, the ICD-code

For peer review only - http://bmjopen.bmj.com/site/about/guidelines.xhtml

AM041 is not the best tool to use for including all women who contact healthcare due to decreased fetal movements, but it is the only code available. Misclassification in the study may be important to consider as some women who sought care due to decreased fetal movements didn't get the ICD-code and were classified as they had not sought care. This might have diluted the differences between the groups of women who sought/not sought care due to decreased fetal movements. However, when comparing the Mindfetalness group with routine care group, only non-differential misclassification is likely, which effect the differences in the same way in the two groups.

The only potential confounder we adjusted for was parity. We do not have information about other possibly confounders, for example, if some of the women had extra appointments in specialist maternity clinics, which may have affected their care-seeking behavior. Extra pre-booked meetings with midwives or obstetricians and investigations with cardiotocography or ultrasound due to other complication can also decrease the need for unscheduled visits at the hospitals.

When evaluating whether Mindfetalness has an effect on women contacting healthcare due to decreased fetal movements, the random allocation, the large sample size, and zero lost to follow-up are strengths of the study. Further, the missing data are negligible, thanks to the unique population-based register in Sweden. While the intention-to-treat design does preserve the advantages of random allocation, it also risks misclassification and the dilution of the results, i.e., it is an underestimation of the true effects. From the pilot study, we know that approximately 75 percent of the Swedish-speaking women use the method [17], and, from the main trial, about 22 percent did not receive a leaflet [19]. Additionally, the information was available in nine languages and, in Stockholm, Sweden, there is a large variety of women who originate from different countries and who speak languages that were not included in the intervention and this may have diluted the effect. This further contributes to the possible dilution of effects.

Less likely to seek care were i) women 35 and over, ii) less educated, and iii) non-Swedish women. Women using Mindfetalness were more likely to visit healthcare-providers due to decreased fetal movements than women in the control-group. It is unclear from this study whether we would have seen the same results if we had used a conventional "count to ten" approach to maternal awareness of fetal movements

For peer review only - http://bmjopen.bmj.com/site/about/guidelines.xhtml

Implications

The National Board of Health and Welfare claim that pregnant women should receive information about risk factors for adverse pregnancy outcomes, including stillbirth [38]. Women born in low-income countries and women with advanced maternal age are factors to consider when trying to improve perinatal mortality in high-income countries [42,46]. Providing targeted information, available in a variety of languages, to these women about fetal movements and when to contact healthcare might help to improve perinatal outcomes. Also, providing information about how to practice Mindfetalness can be one way forward to reduce adverse pregnancy outcomes. Today, we have a great number of opportunities to reach pregnant women thanks to the internet and to target women in at-risk groups. Additionally, this information can be provided in different forms, for example, videos, apps and interactive webpages.

Conclusion

We do not know for certain what the ideal frequency is for women seeking care due to decreased fetal movements, but we can conclude that it is lower in women who have migrated to Sweden from, for example, Africa and Asia, and in women with low educational levels. Women from low-income countries have documented worse pregnancy outcomes. An implication of our results may be to examine whether pregnancy outcomes can be improved by providing customized information to women from low-income countries with the goal that they seek care if they feel a decreased strength or frequency of fetal movements. More data are needed to draw conclusions about the effects of Mindfetalness in perinatal mortality among women contacting healthcare due to decreased fetal movements.

Acknowledgements

The authors would like to thank the midwives and the pregnant women in the maternity clinics in Stockholm. Thanks to the coordination midwives and medical doctors at Mödrahälsovårdsenheten and to The Swedish Pregnancy Register for cooperation. Special thanks to The Swedish Research Council for funding this study.

For peer review only - http://bmjopen.bmj.com/site/about/guidelines.xhtml

References

1. Dutton PJ, Warrander LK, Roberts SA, et al. Predictors of poor perinatal outcome following maternal perception of reduced fetal movements--a prospective cohort study. *PLoS One* 2012; **7**(7): e39784.
2. Higgins LE, Myers JE, Sibley CP, Johnstone ED, Heazell AEP. Antenatal placental assessment in the prediction of adverse pregnancy outcome after reduced fetal movement. *PLoS One* 2018; **13**(11): e0206533.
3. Holm Tveit JV, Saastad E, Stray-Pedersen B, Bordahl PE, Froen JF. Maternal characteristics and pregnancy outcomes in women presenting with decreased fetal movements in late pregnancy. *Acta obstetricia et gynecologica Scandinavica* 2009; **88**(12): 1345-51.
4. Linde A, Pettersson K, Radestad I. Women's Experiences of Fetal Movements before the Confirmation of Fetal DeathContractions Misinterpreted as Fetal Movement. *Birth-Issues in Perinatal Care* 2015; **42**(2): 189-94.
5. Warland J, O'Brien LM, Heazell AE, Mitchell EA. An international internet survey of the experiences of 1,714 mothers with a late stillbirth: the STARS cohort study. *BMC pregnancy and childbirth* 2015; **15**: 172.
6. Froen JF, Arnestad M, Frey K, Vege A, Saugstad OD, Stray-Pedersen B. Risk factors for sudden intrauterine unexplained death: epidemiologic characteristics of singleton cases in Oslo, Norway, 1986-1995. *American journal of obstetrics and gynecology* 2001; **184**(4): 694-702.
7. Koshida S, Ono T, Tsuji S, Murakami T, Arima H, Takahashi K. Excessively delayed maternal reaction after their perception of decreased fetal movements in stillbirths: Population-based study in Japan. *Women and birth : journal of the Australian College of Midwives* 2017.
8. Kapaya H, Almeida J, Karouni F, Anumba D. Management of reduced fetal movement: A comparative analysis of two audits at a tertiary care clinical service. *Eur J Obstet Gynecol Reprod Biol* 2020; **248**: 128-32.
9. Akselsson A, Lindgren H, Georgsson S, Pettersson K, Radestad I. Increased labor induction and women presenting with decreased or altered fetal movements - a population-based survey. *PLoS One* 2019; **14**(5): e0216216.
10. Froen JF. A kick from within--fetal movement counting and the cancelled progress in antenatal care. *J Perinat Med* 2004; **32**(1): 13-24.
11. Sergent F, Lefevre A, Verspyck E, Marpeau L. [Decreased fetal movements in the third trimester: what to do?]. *Gynecologie, obstetrique & fertilite* 2005; **33**(11): 861-9.
12. Radestad I, Lindgren H. Women's perceptions of fetal movements in full-term pregnancy. *Sexual & reproductive healthcare : official journal of the Swedish Association of Midwives* 2012; **3**(3): 113-6.
13. Bradford BF, Cronin RS, McCowan LME, McKinlay CJD, Mitchell EA, Thompson JMD. Association between maternally perceived quality and pattern of fetal movements and late stillbirth. *Scientific reports* 2019; **9**(1): 9815.
14. Radestad I. Strengthening mindfetalness. *Sexual & reproductive healthcare : official journal of the Swedish Association of Midwives* 2012; **3**(2): 59-60.
15. Pearson JF. Fetal movements--a new approach to antenatal care. *Nurs Mirror Midwives J* 1977; **144**(16): 49-51.
16. Sadovsky E, Yaffe H. Daily fetal movement recording and fetal prognosis. *Obstetrics and gynecology* 1973; **41**(6): 845-50.
17. Akselsson A, Georgsson S, Lindgren H, Pettersson K, Radestad I. Women's attitudes, experiences and compliance concerning the use of Mindfetalness- a method for systematic observation of fetal movements in late pregnancy. *BMC pregnancy and childbirth* 2017; **17**(1): 359.

For peer review only

For peer review only - http://bmjopen.bmj.com/site/about/guidelines.xhtml

18. Malm MC, Radestad I, Rubertsson C, Hildingsson I, Lindgren H. Women's experiences of two different self-assessment methods for monitoring fetal movements in full-term pregnancy--a crossover trial. *BMC pregnancy and childbirth* 2014; **14**: 349.

19. Akselsson A, Lindgren H, Georgsson S, et al. Mindfetalness to increase women's awareness of fetal movements and pregnancy outcomes: a cluster-randomised controlled trial including 39 865 women. *BJOG : an international journal of obstetrics and gynaecology* 2020.

20. Radestad I, Akselsson A, Georgsson S, Lindgren H, Pettersson K, Steineck G. Rationale, study protocol and the cluster randomization process in a controlled trial including 40,000 women investigating the effects of mindfetalness. *Sexual & reproductive healthcare : official journal of the Swedish Association of Midwives* 2016; **10**: 56-61.

21. ICD internetmedicin i Göteborg AB. http://icd.internetmedicin.se/ (accessed December 2020).

22. Graviditetsregistret. Graviditetsregistret. https://www.medscinet.com/gr/engelska.aspx (accessed January 2021).

23. Gagnon AJ, Zimbeck M, Zeitlin J, et al. Migration to western industrialised countries and perinatal health: a systematic review. *Soc Sci Med* 2009; **69**(6): 934-46.

24. Saastad E, Vangen S, Froen JF. Suboptimal care in stillbirths - a retrospective audit study. *Acta obstetricia et gynecologica Scandinavica* 2007; **86**(4): 444-50.

25. Khalil A, Rezende J, Akolekar R, Syngelaki A, Nicolaides KH. Maternal racial origin and adverse pregnancy outcome: a cohort study. *Ultrasound Obstet Gynecol* 2013; **41**(3): 278-85.

26. Ravelli AC, Tromp M, Eskes M, et al. Ethnic differences in stillbirth and early neonatal mortality in The Netherlands. *J Epidemiol Community Health* 2011; **65**(8): 696-701.

27. Drysdale H, Ranasinha S, Kendall A, Knight M, Wallace EM. Ethnicity and the risk of late-pregnancy stillbirth. *Med J Aust* 2012; **197**(5): 278-81.

28. Vik ES, Aasheim V, Schytt E, Small R, Moster D, Nilsen RM. Stillbirth in relation to maternal country of birth and other migration related factors: a population-based study in Norway. *BMC pregnancy and childbirth* 2019; **19**(1): 5.

29. Essen B, Bodker B, Sjoberg NO, et al. Are some perinatal deaths in immigrant groups linked to suboptimal perinatal care services? *BJOG : an international journal of obstetrics and gynaecology* 2002; **109**(6): 677-82.

30. Koshida S, Ono T, Tsuji S, Murakami T, Takahashi K. Recommendations for preventing stillbirth: a regional population-based study in Japan during 2007-2011. *Tohoku J Exp Med* 2015; **235**(2): 145-9.

31. RCOG. Reduced Fetal Movements. 2011 https://www.rcog.org.uk/en/guidelines-research-services/guidelines/gtg57/57) (accessed December 2020 .

32. Tveit JV, Saastad E, Stray-Pedersen B, et al. Reduction of late stillbirth with the introduction of fetal movement information and guidelines - a clinical quality improvement. *BMC pregnancy and childbirth* 2009; **9**: 32.

33. Wackers K, Wassen M, Zeegers B, Bude L, Nieuwenhuijze MJ. Effect of the use of a national information brochure about fetal movements on patient delay. *Women and birth : journal of the Australian College of Midwives* 2019; **32**(2): 131-6.

34. Norman JE, Heazell AEP, Rodriguez A, et al. Awareness of fetal movements and care package to reduce fetal mortality (AFFIRM): a stepped wedge, cluster-randomised trial. *Lancet* 2018.

35. Grimes HA, Forster DA, Newton MS. Sources of information used by women during pregnancy to meet their information needs. *Midwifery* 2014; **30**(1): e26-33.

36. McArdle A, Flenady V, Toohill J, Gamble J, Creedy D. How pregnant women learn about foetal movements: sources and preferences for information. *Women and birth : journal of the Australian College of Midwives* 2015; **28**(1): 54-9.

For peer review only

For peer review only - http://bmjopen.bmj.com/site/about/guidelines.xhtml

37. Socialstyrelsen. Minskade fosterrörelser – rekommendationer om information, råd och en inledande bedömning – Kunskapsstöd med nationella rekommendationer. 2016. http://www.socialstyrelsen.se/publikationer2016/2016-10-9 (accessed December 2020).
38. Socialstyrelsen. Dödfödda barn - En inventering och förslag på åtgärder. 2018. https://www.socialstyrelsen.se/publikationer2018/2018-12-36/Sidor/default.aspx (accessed January 2021).
39. Stephansson O, Dickman PW, Johansson AL, Cnattingius S. The influence of socioeconomic status on stillbirth risk in Sweden. *Int J Epidemiol* 2001; **30**(6): 1296-301.
40. Zeitlin J, Mortensen L, Prunet C, et al. Socioeconomic inequalities in stillbirth rates in Europe: measuring the gap using routine data from the Euro-Peristat Project. *BMC pregnancy and childbirth* 2016; **16**: 15.
41. Socialstyrelsen. Socioekonomiska faktorers påverkan på kvinnors och barns hälsa efter förlossning 2016. https://www.socialstyrelsen.se/globalassets/sharepoint-dokument/artikelkatalog/ovrigt/2016-12-14.pdf (accessed April 2021).
42. Flenady V, Middleton P, Smith GC, et al. Stillbirths: the way forward in high-income countries. *Lancet* 2011; **377**(9778): 1703-17.
43. Levy M, Kovo M, Barda G, et al. Reduced fetal movements at term, low-risk pregnancies: is it associated with adverse pregnancy outcomes? Ten years of experience from a single tertiary center. *Archives of gynecology and obstetrics* 2020; **301**(4): 987-93.
44. Carroll L, Gallagher L, Smith V. Risk factors for reduced fetal movements in pregnancy: A systematic review and meta-analysis. *Eur J Obstet Gynecol Reprod Biol* 2019; **243**: 72-82.
45. Moore TR, Piacquadio K. A prospective evaluation of fetal movement screening to reduce the incidence of antepartum fetal death. *American journal of obstetrics and gynecology* 1989; **160**(5 Pt 1): 1075-80.
46. Flenady V, Koopmans L, Middleton P, et al. Major risk factors for stillbirth in high-income countries: a systematic review and meta-analysis. *Lancet* 2011; **377**(9774): 1331-40.

For peer review only - http://bmjopen.bmj.com/site/about/guidelines.xhtml

Author contributions

IR, KP, HL and AA were involved in the design and planning of the cluster-randomized controlled trial and IR obtained funding. AA had overall responsibility for the study. VS and AA analysed the data with input from IR, KP and HL. AA wrote the original manuscript and all authors made critical input. All authors were responsible for drafting and validating the following versions of the manuscript. All authors approved the final version of the manuscript.

Funding: This study was funded by The Swedish Research Council. Grant number not applicable.

Competing interests: The authors declare that they have no competing interests and there are not any financial competing interests.

Patient consent: Not required.

Ethics approval: This study was approved by the Regional Ethics committee in Stockholm, Sweden (Dnr 2015/2105–31/1), 13 January 2016.

Data sharing statement No additional data is available. Anonymised data are available after ethical approval by the Regional Ethics committee in Stockholm, Sweden and after request to The Swedish Pregnancy Register.

Patient and Public Involvement: Patients or the public were not involved in the design, or conduct, or reporting, or dissemination plans of our research.

Figure 1. Flow chart.

Figure 2a. Birth outcome among women who contacted healthcare due to decreased fetal movements from 32 weeks' gestation, where the examination did not indicate that the pregnancy needed to be terminated, at the time the women sought care.

Figure 2b. Start of labor among women who contacted healthcare due to decreased fetal movements from 32 weeks' gestation, where the examination did not indicate that the pregnancy needed to be terminated, at the time the women sought care.

Supplementary Table 1. Percentage of women who sought care due to decreased fetal movements in Mindfetalness (*n*=19 639) versus routine care (*n*=20 226), divided into characteristics and a calculation of absolute differences between the groups.

For peer review only - http://bmjopen.bmj.com/site/about/guidelines.xhtml

For peer review only

Supplementary Table 2. Obstetric outcome from gestation week 32+0 among women with singleton pregnancy who have contacted healthcare due to decreased fetal movements; 1287 registered at a maternity clinic randomized to Mindfetalness, and 772 registered at a maternity clinic randomized to routine care.

For peer review only

For peer review only - http://bmjopen.bmj.com/site/about/guidelines.xhtml

Figure 1. Flow chart.

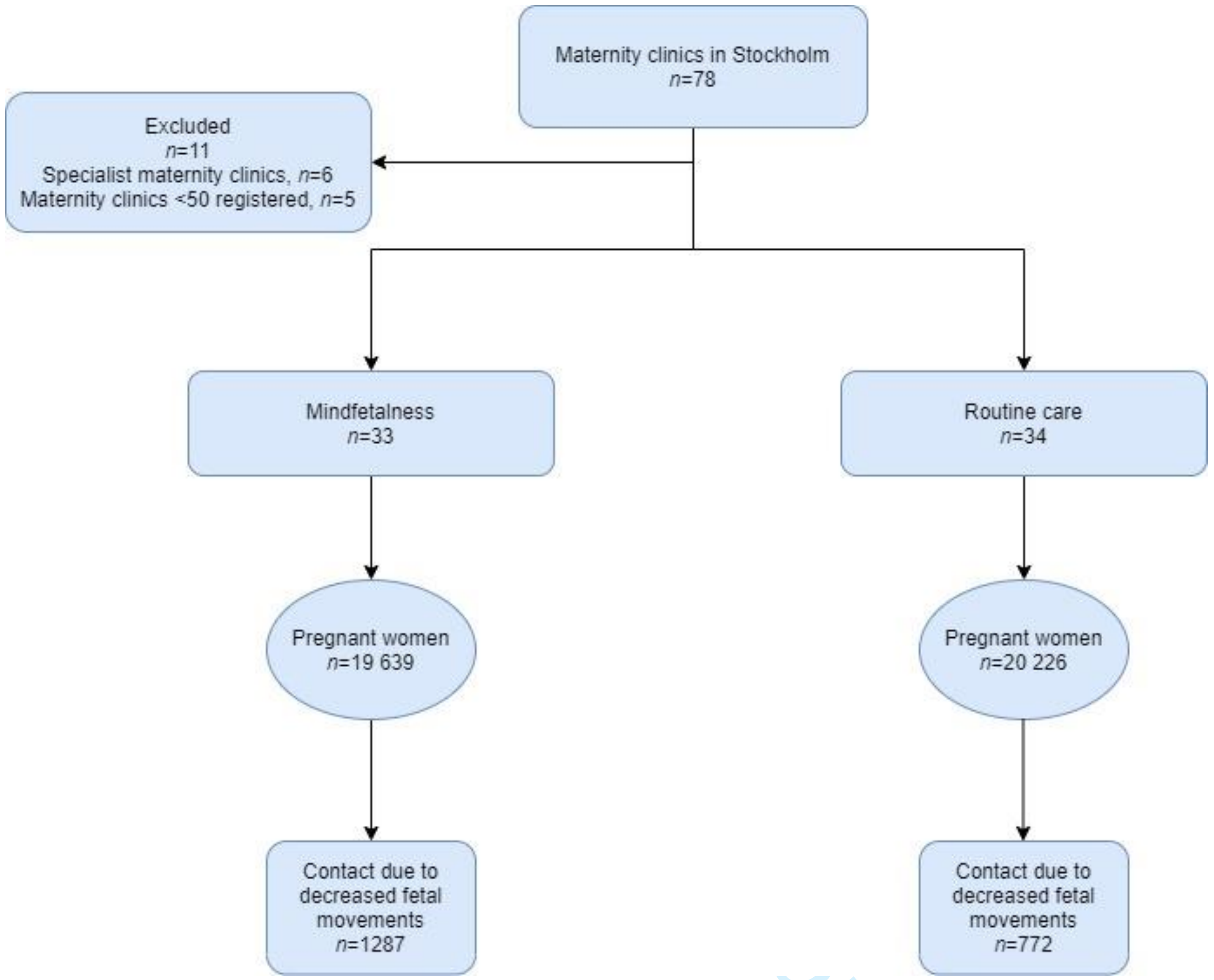

Figure 2a. Birth outcome among women who contacted healthcare due to decreased fetal movements from 32 weeks' gestation, where the examination did not indicate that the pregnancy needed to be terminated, at the time the women sought care.

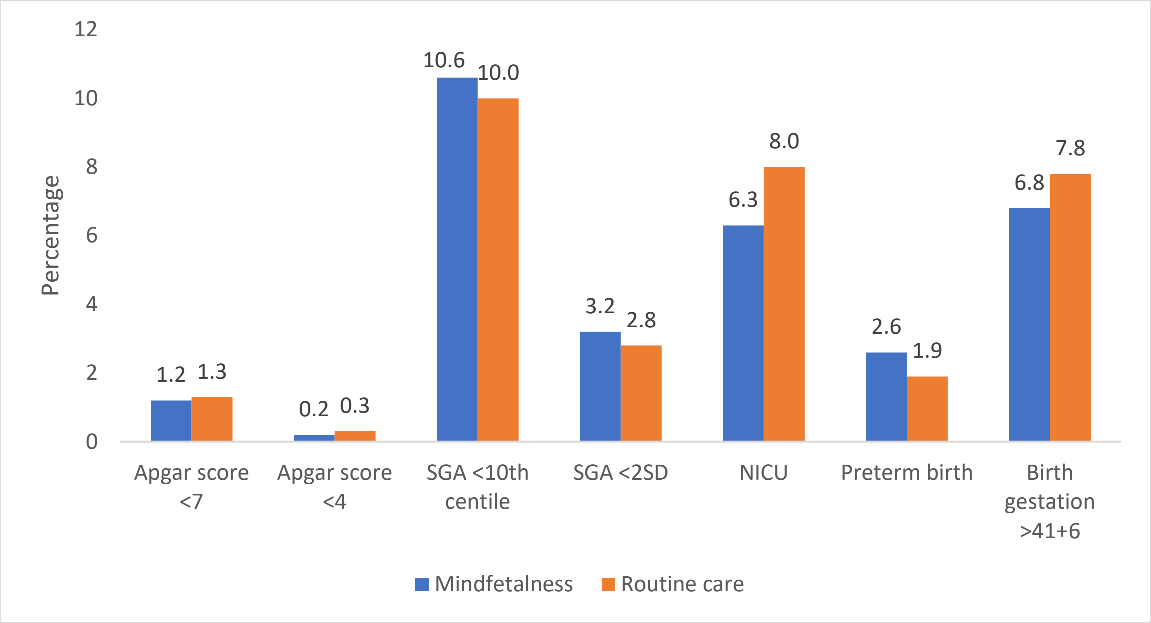

Figure 2b. Start of labor among women who contacted healthcare due to decreased fetal movements from 32 weeks' gestation, where the examination did not indicate that the pregnancy needed to be terminated, at the time the women sought care.

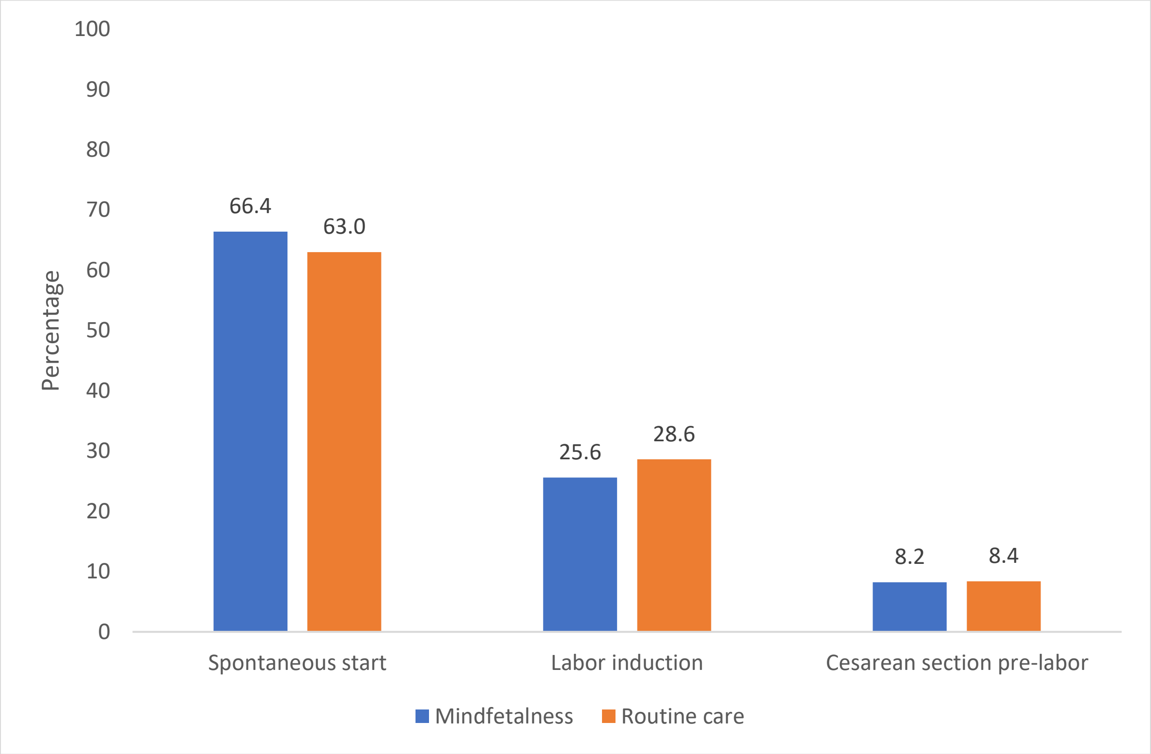

For peer review only - http://bmjopen.bmj.com/site/about/guidelines.xhtml

Supplementary Table 1. Percentage of women who sought care due to decreased fetal movements in Mindfetalness (*n*=19 639) versus routine care (*n*=20 226), divided into characteristics and a calculation of absolute differences between the groups.

**Characteristics**	**Mindfetalness** *n*=1287	**Percentage sought care**	**Routine care** *n*=772	**Percentage sought care**	**Absolute difference**
**Age**					
≤24 years	122/1483	8.2%	65/1322	4.9%	3.3%
25–34 years	859/12 715	6.8%	502/12 783	3.9%	2.9%
≥35 years	306/5441	5.6%	205/6111	3.4%	2.2%
**Country of birth**					
Sweden	1011/13 029	7.8%	584/13 456	4.3%	3.5%
Europe (except Sweden)	82/2026	4.0%	55/1891	2.9%	1.1%
Africa	28/1063	2.6%	23/1438	1.6%	1.0%
Asia	134/2989	4.5%	90/2898	3.1%	1.4%
North America	9/166	5.4%	7/154	4.5%	0.9%
South America	19/328	5.8%	12/325	3.7%	2.1%
Other	4/38	10.5%	1/64	1.6%	8.9%
**Education level¶**					
Shorter than 9 years	4/203	2.0%	7/359	1.9%	0.1%
Elementary school	48/916	5.2%	21/887	2.4%	2.8%
High school	339/5192	6.5%	207/5071	4.1%	2.4%
University	829/11 916	7.0%	461/12 088	3.8%	3.2%
**Parity §**					
Primipara	686/8544	8.0%	441/8927	4.9%	3.1%
Multipara	600/11 012	5.4%	330/11 242	2.9%	2.5%
**Assisted reproduction§**	79/1078	7.3%	50/1166	4.3%	3.0%
**Previous stillbirth**	9/116	7.8%	0/104	0%	7.8%
**Tobacco user at registration at the maternity clinic§**	56/737	7.6%	23/598	3.8%	3.8%
**Body Mass Index∫**					
<18.5 kg/m²	38/568	6.7%	16/507	3.2%	3.5%
18.5–24.9 kg/m²	771/11 828	6.5%	457/12 024	3.8%	2.7%
25.0–29.9 kg/m²	285/4336	6.6%	179/4739	3.8%	2.8%
30.0–34.9 kg/m²	107/1488	7.2%	60/1568	3.8%	3.4%
≥35.0 kg/m²	38/533	7.1%	25/530	4.7%	2.4%
**Maternal diseases***					
Psychiatric care	225/2468	9.1%	129/2630	4.9%	4.2%
Medication or psychologic treatment for mental illness	82/1160	7.1%	47/935	5.0%	2.1%

¶ Data are missing for 3,344 women (1,412 in Mindfetalness group and 1,932 in Routine care group)
§ Data are missing for 140 women (83 in Mindfetalness group and 57 in Routine care group)
∫ Data are missing for 1,744 women (886 in Mindfetalness group and 858 in Routine care group)
*Selected common diseases that can be associated with search behavior

For peer review only

Supplementary Table 2. Obstetric outcome from gestation week 32+0 among women with singleton pregnancy who have contacted healthcare due to decreased fetal movements; 1287 registered at a maternity clinic randomized to Mindfetalness, and 772 registered at a maternity clinic randomized to routine care.

**Outcome**	**Mindfetalness** ***n* (%)**	**Routine care** ***n* (%)**	**Rate Ratio (95% CI)**	***p*-value**
Apgar Score <10 at 5 minutes†	147 (11.4)	88 (11.4)	1.00 (0.79–1.29)	0.97
Apgar Score <7 at 5 minutes†	15 (1.2)	10 (1.3)	0.90 (0.41–2.06)	0.80
Apgar Score < 4 at 5 minutes†	3 (0.2)	2 (0.3)	0.90 (0.15–6.84)	0.91
Birthweight <10th centile*‡	136 (10.6)	77 (10.0)	1.06 (0.82–1.39)	0.67
Birthweight <2SD**‡	41 (3.2)	22 (2.8)	1.12 (0.68–1.89)	0.67
Admitted to NICU	81 (6.3)	62 (8.0)	0.78 (0.57–1.08)	0.14
Death within 27 days after birth	0 (0.0)	0 (0.0)	NA	NA
Preterm delivery (<37+0)	34 (2.6)	15 (1.9)	1.36 (0.76–2.55)	0.31
Birth gestation >41+6	87 (6.8)	60 (7.8)	0.87 (0.64–1.20)	0.39
Spontaneous start of labor	854 (66.4)	486 (63.0)	1.05 (0.99–1.13)	0.12
Induction of labor	329 (25.6)	221 (28.6)	0.89 (0.77–1.03)	0.13
Cesarean section (total)	251 (19.5)	154 (19.9)	0.98 (0.82–1.17)	0.81
Pre–labor	106 (8.2)	65 (8.4)	0.98 (0.73–1.32)	0.88
In labor	145 (11.3)	89 (11.5)	0.98 (0.76–1.26)	0.86

Stillborn routine care *n*=1 (0.1%), Mindfetalness *n*=0 (0.0)
†Data are missing for 3 women (3 in Mindfetalness group and 0 in Routine care group)
‡Data are missing for 2 women (1 in Mindfetalness group and 1 in Routine care group)
*for the gestational age (International definition of Small for Gestational Age)
**from the national reference mean (Swedish definition of Small for Gestational Age)
NICU: Neonatal intensive care unit

For peer review only

Mindfetalness – a method for focusing upon fetal movements

The first perception of fetal movements is sometimes described as a gentle tickling. As the pregnancy proceeds and the fetus develops the movements become more distinct. Just as new-born babies vary, there are differences between fetuses. Some fetuses are very active during their time in the uterus whilst others are calm, but all fetuses move up until birth. Each unborn baby has its own pattern of movements and at the end of the pregnancy the pattern can be recognised. The fetus moves between periods of wakefulness with much movement and calmer periods of rest. Movement frequency usually peaks around week 32 of pregnancy and remains for the most part at that level until delivery. The movements increase in strength as the fetus grows but at the end of the pregnancy can be experienced differently to the movements felt earlier.

That the fetus moves is a good sign and many pregnant women describe how they notice their unborn baby's movements every day. One systematic way of observing the movements is the method of *Mindfetalness* that can be used to get to know the movement pattern. An appropriate time to begin with *Mindfetalness* is in gestational week 28.

How to put Mindfetalness into practice

Mindfetalness is practiced ideally on a daily basis. **Choose a time of day that suits you best but also wait until you feel that your unborn baby is having a period of wakefulness.** Lie down or sit comfortably when you engage in Mindfetalness. If you lie down, then preferably on your left side. The movements are felt more distinctly when you lie down, and the blood flow is at its best in the uterus on the left side, which is good for the fetus. Concentrate on your unborn baby's movements for approximately 15 minutes. You will feel yourself if you need a longer or shorter time to perceive the movements. For some women it is enough just to get an idea of how the movements feel, others prefer to write notes about what they experience. At the end of this information there is space in which you can write down how you experience the movements. If you have a smartphone or computer you can write your impressions at www.mindfetalness.com

During Mindfetalness you focus upon:
The intensity of the movements
The way in which the baby moves
How much the baby moves

The questions to be answered are:
Can the movements be felt distinctly?
Are the movements of the same intensity as usual?
Does the fetus move as much as usual?

For peer review only - http://bmjopen.bmj.com/site/about/guidelines.xhtml

Fetal movements at the end of the pregnancy

The unborn baby's movements can be divided into two main groups: large movements and small movements. The large movements are felt distinctly; this can be when the fetus kicks or stretches out its body. The small movements that the fetus makes, but which are not felt, are gripping movements with fingers and toes as well as breathing movements. In approximately weeks 25 to 30 the movements begin to become organized and the unborn baby has periods of wakefulness interspersed with periods of rest lasting approximately 40 minutes up to an hour.

The movements change as the pregnancy proceeds and can feel different due to the fact that the space the fetus has at its disposal becomes smaller, although this does not affect the frequency of the movements. Women at full term pregnancy often describe how the movements feel powerful, pushing, stretching, large, from side to side, slow and light.

If you focus upon the fetal movements for a while every day when the fetus has a period of wakefulness, you can gain a good understanding of your unborn baby's movement pattern. It is important the observation occurs when the fetus is awake (the fetus is less active during a rest period). There can be wide variations from fetus to fetus regarding the frequency and intensity of the movements.

Women who have tried *Mindfetalness* describe how they felt calm, present and focussed while using the method. They also describe the period as a communication with their unborn baby and that they experienced a powerful bonding with their baby. Only you can decide whether the method suits you.

For peer review

Summary

The movements become organised during pregnancy weeks 25 to 30 and the fetus has periods of wakefulness interspersed with periods of rest, approximately 40 minutes up to an hour. Most fetuses have, at the end of the pregnancy, a daily rhythm and are active in the evening.

In pregnancy week 32 a plateau phase is reported regarding the frequency of movements but there is nothing to indicate that the movements decrease at the end of pregnancy.

There can be a wide variation between fetuses in the frequency and intensity of their movements.

Get to know your unborn baby's movement pattern during pregnancy. Trust your intuition.

If you are concerned that the fetus is moving less or that the movements are weaker, you should contact health care.

What does the fetus do in the uterus?
As the fetus grows, the movements become more distinct and successively regular. Small movements are not felt, e.g. when the fetus sucks its thumb or flexes its toes. Kicks, and when the child stretches out, can usually be felt clearly and many also feel when the child hiccups (small rhythmic jerks) during the latter part of pregnancy.

During the final months of the pregnancy the movements are distinct and powerful, but may be experienced as being of a different character compared with when the fetus had a larger space at its disposal. Some women describe how the unborn baby stretches out, as if the fetus is trying to stretch as the space begins to be tight. Others describe the movements as large, that they involve the unborn baby's whole body and can be described as slow. The larger the fetus the more distinct the movement.

Is it true that the fetus moves less towards the end of pregnancy?
No, this is not true. Fetal movements increase up until pregnancy week 32, thereafter and up until delivery, the frequency of movements generally remains the same. It is important to remember that the fetus should continue to be active throughout the pregnancy.

Does the fetus move the whole time?
The fetus does not move the whole time. All unborn babies are calm and sleep for short periods. There can be wide variations from fetus to fetus regarding the frequency and intensity of the movements.

Can it be more difficult for some women to feel the movements?
It is probable that it is easier to feel the movements if the woman lies on her left side and concentrates on them. Some women describe how, in spite of doing so, they have great difficulty in feeling their unborn baby move. Extreme overweight, amongst other things, can make it more difficult. If one is much stressed it may be more difficult to feel the movements.

What should I do if I feel that the movements become fewer towards the end of the pregnancy?
If the movements decrease in intensity or frequency, and deviate from the fetus' normal way of moving, it can be a sign that the child is not doing so well in the uterus. Most pregnant women who experience fewer and weaker movements give birth to a healthy child, but there is an increased risk that the fetus is not fit. If you experience that the movements have become fewer and weaker, and you feel that there is a difference compared with earlier in the pregnancy it should *not* be interpreted as something normal until the child has been examined.

For peer review only - http://bmjopen.bmj.com/site/about/guidelines.xhtml

For peer review only

Diary of my unborn baby's activities

Pregnancy week	How my unborn baby moved
28 +	
Pregnancy week 29 +	How my unborn baby moved
Pregnancy week 30 +	How my unborn baby moved
Pregnancy week 31 +	How my unborn baby moved

For peer review only

For peer review only - http://bmjopen.bmj.com/site/about/guidelines.xhtml

Diary of my unborn baby's activities

Pregnancy week	How my unborn baby moved
32 +	
**Pregnancy week** 33 +	**How my unborn baby moved**
**Pregnancy week** 34 +	**How my unborn baby moved**
**Pregnancy week** 35 +	**How my unborn baby moved**

For peer review only

For peer review only - http://bmjopen.bmj.com/site/about/guidelines.xhtml

Diary of my unborn baby's activities

Week of pregnancy	How my unborn baby moved
36 +	
37 +	
38 +	
39 +	

For peer review only

For peer review only - http://bmjopen.bmj.com/site/about/guidelines.xhtml

Diary of my unborn baby’s activities

Week of pregnancy 40 +	How my unborn baby moved
**Week of pregnancy 41 +**	**How my unborn baby moved**
**Week of pregnancy 42 +**	**How my unborn baby moved**

For peer review only

For peer review only - http://bmjopen.bmj.com/site/about/guidelines.xhtml

STROBE 2007 (v4) checklist of items to be included in reports of observational studies in epidemiology*

Checklist for cohort, case-control, and cross-sectional studies (combined)

Section/Topic	Item #	Recommendation	Reported on page #
**Title and abstract**	1	(*a*) Indicate the study's design with a commonly used term in the title or the abstract	1-2
		(*b*) Provide in the abstract an informative and balanced summary of what was done and what was found	2
**Introduction**			
Background/rationale	2	Explain the scientific background and rationale for the investigation being reported	4
Objectives	3	State specific objectives, including any pre-specified hypotheses	4
**Methods**			
Study design	4	Present key elements of study design early in the paper	5
Setting	5	Describe the setting, locations, and relevant dates, including periods of recruitment, exposure, follow-up, and data collection	5 and in previous papers
Participants	6	(*a*) *Cohort study*—Give the eligibility criteria, and the sources and methods of selection of participants. Describe methods of follow-up<br>*Case-control study*—Give the eligibility criteria, and the sources and methods of case ascertainment and control selection. Give the rationale for the choice of cases and controls<br>*Cross-sectional study*—Give the eligibility criteria, and the sources and methods of selection of participants	5
		(*b*) *Cohort study*—For matched studies, give matching criteria and number of exposed and unexposed<br>*Case-control study*—For matched studies, give matching criteria and the number of controls per case	
Variables	7	Clearly define all outcomes, exposures, predictors, potential confounders, and effect modifiers. Give diagnostic criteria, if applicable	5-6
Data sources/ measurement	8*	For each variable of interest, give sources of data and details of methods of assessment (measurement). Describe comparability of assessment methods if there is more than one group	*5-6*
Bias	9	Describe any efforts to address potential sources of bias	5-6 and in previous papers
Study size	10	Explain how the study size was arrived at	5 and in previous papers
Quantitative variables	11	Explain how quantitative variables were handled in the analyses. If applicable, describe which groupings were chosen and why	6
Statistical methods	12	(*a*) Describe all statistical methods, including those used to control for confounding	6
		(*b*) Describe any methods used to examine subgroups and interactions	6

For peer review only

		(*c*) Explain how missing data were addressed	Negligible due to unique population-based register
		(*d*) *Cohort study*—If applicable, explain how loss to follow-up was addressed<br>*Case-control study*—If applicable, explain how matching of cases and controls was addressed<br>*Cross-sectional study*—If applicable, describe analytical methods taking account of sampling strategy	6
		(*e*) Describe any sensitivity analyses	
**Results**			
Participants	13*	(a) Report numbers of individuals at each stage of study—eg numbers potentially eligible, examined for eligibility, confirmed eligible, included in the study, completing follow-up, and analysed	6-9
		(b) Give reasons for non-participation at each stage	NA
		(c) Consider use of a flow diagram	Yes, included
Descriptive data	14*	(a) Give characteristics of study participants (eg demographic, clinical, social) and information on exposures and potential confounders	6-7 and Suppl. Table 1
		(b) Indicate number of participants with missing data for each variable of interest	6-9
		(c) *Cohort study*—Summarise follow-up time (eg, average and total amount)	
Outcome data	15*	*Cohort study*—Report numbers of outcome events or summary measures over time	6-9
		Case-control study—Report numbers in each exposure category, or summary measures of exposure	
		Cross-sectional study—Report numbers of outcome events or summary measures	
Main results	16	(*a*) Give unadjusted estimates and, if applicable, confounder-adjusted estimates and their precision (eg, 95% confidence interval). Make clear which confounders were adjusted for and why they were included	8
		(*b*) Report category boundaries when continuous variables were categorized	Yes, 6-9
		(*c*) If relevant, consider translating estimates of relative risk into absolute risk for a meaningful time period	Yes, 6-9
Other analyses	17	Report other analyses done—eg analyses of subgroups and interactions, and sensitivity analyses	Suppl. Table 1
**Discussion**			
Key results	18	Summarise key results with reference to study objectives	9-10
Limitations	19	Discuss limitations of the study, taking into account sources of potential bias or imprecision. Discuss both direction and magnitude of any potential bias	11-13
Interpretation	20	Give a cautious overall interpretation of results considering objectives, limitations, multiplicity of analyses, results from similar studies, and other relevant evidence	13
Generalisability	21	Discuss the generalisability (external validity) of the study results	13
**Other information**			
Funding	22	Give the source of funding and the role of the funders for the present study and, if applicable, for the original study on which the present article is based	17

For peer review only

*Give information separately for cases and controls in case-control studies and, if applicable, for exposed and unexposed groups in cohort and cross-sectional studies.
Note: An Explanation and Elaboration article discusses each checklist item and gives methodological background and published examples of transparent reporting. The STROBE checklist is best used in conjunction with this article (freely available on the Web sites of PLoS Medicine at http://www.plosmedicine.org/, Annals of Internal Medicine at http://www.annals.org/, and Epidemiology at http://www.epidem.com/). Information on the STROBE Initiative is available at www.strobe-statement.org.

For peer review only
